# Computation and Structure‐Guided Arginine Scanning Engineers a Hyperactive AP Endonuclease for Multiplex Viral RNA Sensing

**DOI:** 10.1002/advs.76769

**Published:** 2026-07-24

**Authors:** Junlan Wang, Ting Wu, Feizuo Wang, Boyang Wang, Xin Yu Wong, Senfeng Zhang, Shengsheng Ma, Yao Liu, Fangyao Yao, Zhifeng Zeng, Siyuan Yang, Kunming Liu, Qi Wang, Yilong Wang, Yunxiang Zang, Xiaolong Wang, Xingxu Huang, Shao Li, Quanquan Ji, Tao Hu, Chunyi Hu

**Affiliations:** ^1^ Department of Biological Sciences Faculty of Science National University of Singapore Singapore Singapore; ^2^ National Clinical Research Center for Child Health Children's Hospital Zhejiang University School of Medicine Hangzhou China; ^3^ Institute for TCM‐X MOE Key Laboratory of Bioinformatics Bioinformatics Division BNRist Department of Automation Tsinghua University Beijing China; ^4^ International Joint Agriculture Research Center for Animal Bio‐Breeding of Ministry of Agriculture and Rural Affairs College of Animal Science and Technology Northwest A&F University Yangling Shaanxi China; ^5^ Department of Biochemistry Yong Loo Lin School of Medicine National University of Singapore Singapore Singapore; ^6^ The Research Center of Chiral Drugs Innovation Research Institute of Traditional Chinese Medicine Shanghai University of Traditional Chinese Medicine Shanghai China; ^7^ The Key Laboratory of Pancreatic Diseases of Zhejiang Province The First Affiliated Hospital Zhejiang University School of Medicine Hangzhou China; ^8^ Cancer Science Institute of Singapore National University of Singapore Singapore Singapore; ^9^ Precision Medicine Translational Research Programme (TRP) Yong Loo Lin School of Medicine National University of Singapore Singapore Singapore

**Keywords:** AI‐guided Arg scanning, AP endonuclease, APE1 engineering, dengue and influenza viruses, nucleic acid detection, viral RNA sensing

## Abstract

DNA‐binding proteins such as AP endonucleases are powerful scaffolds for biotechnology, but there is no general way to rewire their DNA‐contact surfaces. Here we report ARGENT (AI‐guided Arginine scanning Engine for Nucleic‐acid Tuning), an interpretable framework that uses protein–DNA structures and homologous sequences to propose beneficial Arg substitutions and multi‐residue charge patches. Applied to the APE1–DNA complex, ARGENT combines structural and evolutionary features into a residue‐wise hotspot score, compressing 276 candidate positions into 9 sites for testing. Six single mutants increase AP‐site cleavage, and the top‐ranked triple mutant, APE1‐Evo, connects three DNA‐contact patches, boosts the single‐turnover rate constant ∼4‐fold relative to wild‐type APE1, and preserves mismatch discrimination at the AP‐opposite position. We embed APE1‐Evo into an upgraded NAPTUNE‐V2.0 architecture that couples AP‐site cleavage to a multilayer *Pf*Ago cascade, enabling multiplex detection of dengue virus pseudotypes and direct typing of influenza A and B viruses in clinical RNA with high concordance to qPCR. ARGENT also transfers to other DNA‐binding proteins, including *MG34‐1* Cas9d, highlighting its broader relevance to genome‐editing enzymes. These results establish AI‐guided Arg scanning as a practical route to engineer hyperactive, high‐fidelity AP endonucleases and to tune DNA‐binding interfaces for next‐generation nucleic‐acid technologies and viral RNA sensing.

## Graphical abstract

Computational and structure‐guided arginine scanning rewires the DNA‐binding interface of APE1 to create APE1‐Evo, a hyperactive yet specific AP endonuclease. Integrated into the NAPTUNE‐V2.0 cascade, APE1‐Evo enables amplification‐free, multiplex viral RNA sensing for dengue virus and influenza A/B, highlighting a general strategy for engineering nucleic‐acid‐interacting enzymes.

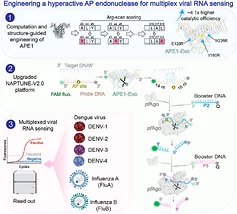

## 1 Introduction

DNA‐binding proteins are core components of genome maintenance, gene regulation and nucleic‐acid technologies [1, 2, 3, 4, 5, 6, 7, 8, 9]. Their engineering has largely relied on structure‐guided mutagenesis of a few selected residues or on large random and semi‐rational libraries coupled to screening or selection [10, 11, 12, 13]. These strategies have been powerful, but they do not provide a systematic way to remodel DNA‐contact surfaces: rational design samples only a tiny fraction of sequence space, whereas large libraries are experimentally intensive and hard to couple to clean read‐outs when the aim is to fine‐tune activity [10]. A widely used intuitive tactic is to enrich DNA‐facing patches in basic residues by converting neutral or acidic side chains into arginine (R) or lysine (K), thereby strengthening interactions with the negatively charged phosphate backbone [14, 15, 16, 17] (Figure S1A). Yet such “charge‐enrichment” designs are typically implemented ad hoc by visual inspection, with little guidance on which positions are structurally and evolutionarily permissible, how many substitutions a patch can tolerate, or how sites should be combined. Despite their ubiquity, these efforts have lacked a general AI‐assisted framework for systematic Arg scanning of DNA‐binding interfaces.

Here we introduce ARGENT (AI‐guided Arginine‐scanning Engine for Nucleic‐acid Tuning), an interpretable AI‐guided Arg‐scanning framework that addresses this gap. ARGENT uses only a protein–DNA structure and homologous sequences as input and assigns to each residue a “virtual Arg” score estimating the benefit of an arginine substitution. We focused on arginine because Arg residues are frequently enriched at protein–nucleic acid interfaces and, through their guanidinium group, can form stronger electrostatic and hydrogen‐bonding interactions with DNA. This feature may provide advantages over lysine for strengthening productive protein–DNA engagement. This score integrates basic structural descriptors—such as distance to DNA phosphates [18], solvent exposure [19], and local environment—with simple sequence‐derived features, including residue‐wise conservation [20, 21]. Nearby high‐scoring residues are then grouped into DNA‐contact patches, and multi‐residue constellations are evaluated with a lightweight combination model that favours balanced distributions of positive charge across distinct patches while penalising overloading of any single region. The result is a compact, ranked set of single positions and multi‐site designs predicted to enhance DNA engagement with minimal collateral cost.

We selected human AP endonuclease 1 (APE1) as a first test case for ARGENT. APE1 is the major apurinic/apyrimidinic (AP) site endonuclease in base excision repair, where it incises the DNA backbone at AP lesions and licenses downstream repair steps [22, 23, 24, 25]. In our previously reported NAPTUNE platform, an AP‐containing DNA probe hybridises to complementary RNA or single‐stranded DNA, is cleaved by APE1, and releases short DNA fragments that program a secondary nuclease such as *Pf*Ago [8, 26, 27], thereby amplifying the signal from an initial binding event [28]. Although this architecture already enables sensitive detection, wild‐type APE1 leaves substantial room for improvement in reaction speed and signal amplitude, particularly in multi‐layer cascades and challenging sample matrices. Applying ARGENT to the APE1‐DNA complex [24], we virtually mutate each residue from 43 to 318 to arginine, compute a geometry‐ and context‐based hotspot score for every site and filter these scores with conservation information to avoid highly constrained positions. This procedure collapses 276 candidate residues to a small panel with favourable DNA geometry and acceptable evolutionary flexibility [21]. From this panel, we select only 9 positions for experimental testing and find that 6 corresponding single mutants display substantially enhanced AP‐site cleavage. Patch‐level analysis of multi‐site designs on the DNA‐binding surface then identifies a triple mutant, E126R+V180R+N226R (hereafter APE1‐Evo), that bridges three distinct DNA‐contact patches. Under identical condition, APE1‐Evo exhibits an over 4‐fold increase in apparent rate constant relative to wild‐type APE1 while maintaining strict discrimination against mismatches opposite the AP site, demonstrating that catalytic output can be boosted without compromising fidelity.

To demonstrate functional impact, we embed APE1‐Evo into an upgraded NAPTUNE‐V2.0 architecture, in which AP‐site cleavage is coupled to a multi‐layer *Pf*Ago amplification cascade. This configuration yields stronger signal gain than the original platform and enables multiplex detection of dengue virus pseudotypes [29, 30, 31, 32], as well as direct typing of influenza A (Flu A) [33, 34, 35] and B (Flu B) [36, 37] viruses in clinical RNA samples with high concordance to RT–qPCR. Finally, we show that the ARGENT workflow can be readily applied to other DNA‐binding proteins, including the gene‐editing nuclease Cas9 [13, 38, 39, 40], highlighting its potential as a general design principle for tuning DNA affinity and specificity. Together, these results establish AI‐guided Arg scanning as a practical route to engineer hyperactive yet high‐fidelity AP endonucleases for multiplex viral RNA sensing and, more broadly, to rationally reprogram DNA‐binding interfaces in repair nucleases and genome‐editing enzymes for next‐generation nucleic‐acid technologies.

## 2 Results

### 2.1 ARGENT Computational Framework‐Guided Arginine Scanning Maps APE1 DNA‐Contact Hotspots

To enable rapid in silico evolution of DNA‐binding proteins, we sought a general framework that could systematically redesign DNA‐contact surfaces rather than relying on ad hoc mutations. We developed ARGENT (AI‐guided Arginine‐scanning Engine for Nucleic‐acid Tuning) and used human APE1, a central enzyme in base‐excision repair and a versatile AP‐site sensor [24], as a testbed (Figure S1B). Starting from the APE1–DNA co‐crystal structure, ARGENT virtually substituted each residue from 43 to 318 with arginine and computed an interpretable “virtual Arg” hotspot score that integrates distance to DNA phosphates, surface exposure and multi‐scale neighbourhood statistics, followed by filtering with residue‐wise conservation and a simple solubility predictor (Figure 1A).

**FIGURE 1:**
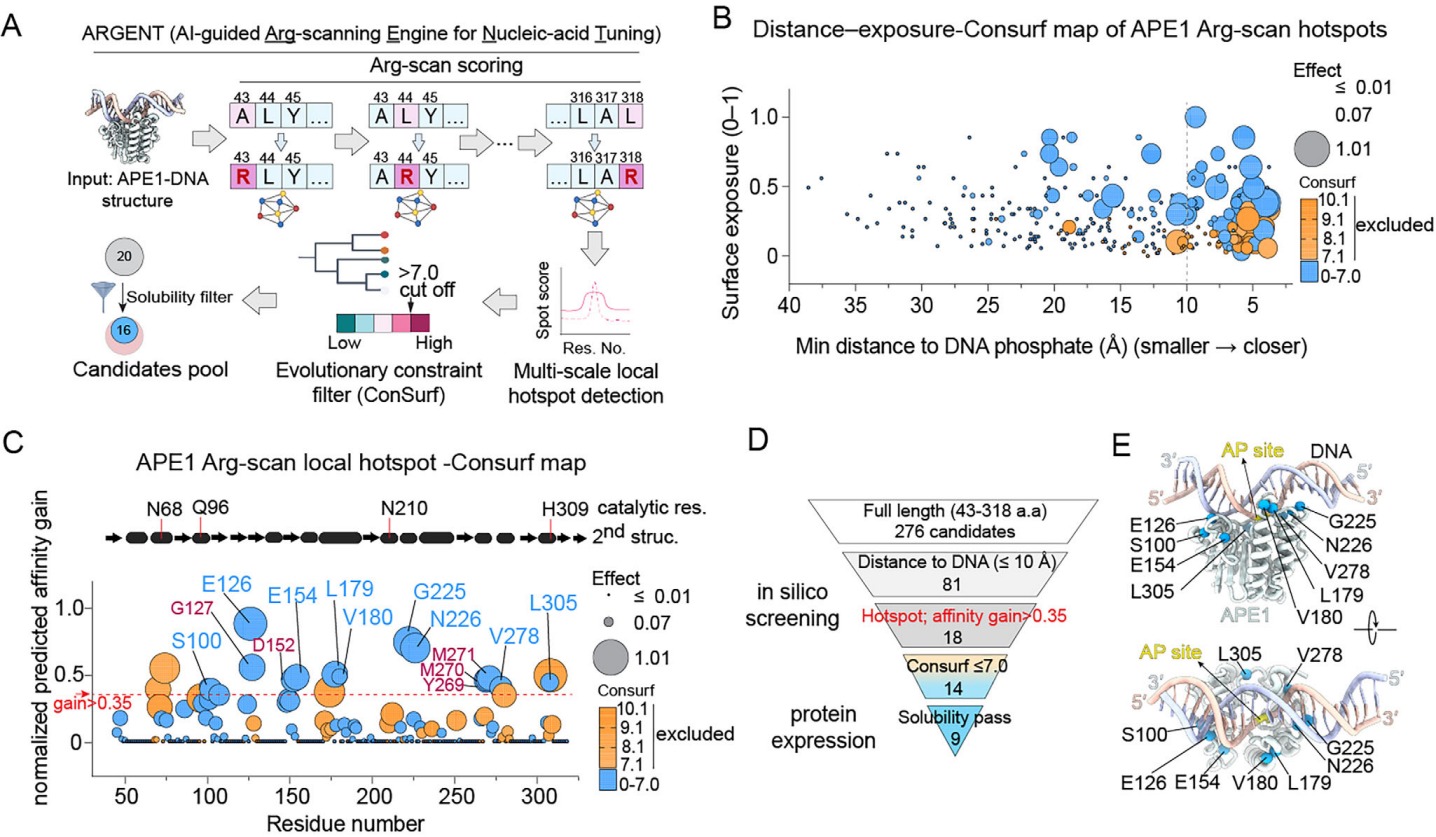
ARGENT AI‐guided Arg scanning maps tunable DNA‐contact hotspots in APE1. (A) Schematic of the ARGENT pipeline. The APE1–DNA complex is used as input, and every residue from 43 to 318 is virtually mutated to Arg, yielding 276 single‐site “virtual Arg” variants. For each position, ARGENT computes an interpretable hotspot score that integrates distance to DNA phosphates, surface exposure, and local neighbourhood statistics. Residue‐wise evolutionary constraints from ConSurf are then applied as a filter (excluding highly conserved sites with grade >7.0), and a multi‐scale local hotspot detector (window sizes w8 and w18) highlights residues embedded in favorable local environments. Positions that pass these steps constitute a candidate pool, which is further pruned by a simple solubility filter to define experimentally testable mutants. (B) Distance–exposure–ConSurf map of ARGENT hotspots. Each bubble corresponds to one residue in APE1. The *x*‐axis shows the minimum distance from the residue to any DNA phosphate (smaller values indicate closer approach), and the *y*‐axis shows normalized surface exposure. Bubble size encodes the predicted effect of Arg substitution on DNA engagement, and color indicates ConSurf conservation grade. Highly conserved residues (grade >7.0; gray/orange) are excluded from downstream design. (C) ARGENT local hotspot–ConSurf map along the APE1 primary sequence. The *x*‐axis shows residue number, and the *y*‐axis shows normalized predicted affinity gain upon Arg substitution, combining multi‐scale hotspot scores (w8 and w18). Bubble size reflects the predicted effect, and color reports ConSurf conservation. Catalytic residues and secondary‐structure elements are indicated schematically above. Blue labels mark sites selected for biochemical testing; magenta labels denote top‐scoring but highly conserved residues that were excluded. (D) In silico screening funnel implemented by ARGENT. From 276 residues (43–318), 81 positions within 10 Å of any DNA phosphate are retained. Applying an affinity‐gain threshold (hotspot score ≥0.35) yields 18 positions, of which 14 satisfy the ConSurf cutoff (grade ≤7.0); 9 of these are additionally predicted to be compatible with solubility and are taken forward for experimental validation. (E) Structural localization of ARGENT‐selected positions in APE1. Upper view: APE1–DNA complex with the AP site highlighted in yellow and the nine selected sites shown as spheres around the DNA‐binding surface. Lower view: rotated close‐up illustrating how E126, S100, E154, L179, V180, G225, N226, V278, and L305 encircle the AP site and duplex, forming a designed positive‐charge shell poised to enhance DNA engagement.

We first visualized the structural landscape of these virtual Arg substitutions in a distance‐exposure map, where each residue is plotted by its minimum distance to any DNA phosphate and its normalized surface exposure, with bubble size and color reporting predicted effect and ConSurf grade, respectively (Figure 1B; Figure S1C). High‐scoring sites concentrate in solvent‐exposed regions within ∼10 Å of the DNA backbone, whereas deeply buried or highly conserved residues contribute little to the candidate pool (Figure 1B). Plotting the same hotspot scores along the APE1 sequence revealed discrete clusters of Arg‐tunable residues across the DNA‐binding surface; the multi‐scale local hotspot detector (windows w8 and w18) highlighted neighbourhoods enriched in high‐scoring positions, while conservation cleanly separated structurally attractive but highly conserved sites (orange) from permissive candidates (blue) (Figure 1C). The ARGENT funnel efficiently compressed the design space: of 276 residues, 81 lay within 10 Å of DNA, 18 passed an affinity‐gain threshold, 14 satisfied the ConSurf cut‐off, and 9 additionally met solubility criteria and were taken forward for biochemical testing (Figure 1D). When mapped back onto the structure, these nine sites‐S100, E126, E154, L179, V180, G225, N226, V278 and L305—form three contiguous DNA‐contact patches that wrap around the AP site and along the duplex, outlining a rationally designed positive‐charge shell poised to enhance DNA engagement (Figure 1E; Figure S2). These analyses establish ARGENT as a practical AI‐guided Arg‐scanning framework that can rapidly nominate a small, spatially coherent set of Arg‐tunable hotspots for engineering APE1 and, by extension, other DNA‐binding proteins.

### 2.2 Biochemical Validation of ARGENT‐Selected APE1 Mutants Reveals Three Productive Patches

Having narrowed the APE1–DNA interface to nine ARGENT‐selected positions and purified them successfully (Figure S3), we next asked whether Arg substitution at these sites improves AP‐site cleavage in a probe‐based assay. We used a FAM‐labeled duplex [41] in which an AP is embedded in the probe strand opposite a defined target strand; cleavage by APE1 converts the 30‐nt probe into a 14‐nt product that can be quantified by denaturing PAGE [42] (Figure 2A). Under identical conditions (50 nm APE1 variant, 50 nm DNA duplex), all nine single mutants produced the expected 14‐nt band, indicating that Arg substitution at these sites does not abolish catalysis (Figure 2B). Quantification across multiple replicates revealed that 6 of 9 mutants display clear activity gains relative to wild‐type APE1 (Figure 2C). E126R showed the strongest enhancement, reaching ∼150% of wild‐type cleavage efficiency, followed by N226R, V180R, G225N and L179R, which all clustered around ∼120%–145%. L305R exhibited a more modest but reproducible increase, whereas S100R and V278R were impaired, yielding ∼60% and ∼50% of wild‐type activity, respectively (Figure 2C). This is likely because local steric constraints, native side‐chain packing, or substrate positioning effects are not fully captured by the current scoring function. Future refinement incorporating local structural relaxation and energetic filtering may help reduce such false positives. Overall, despite being selected purely in silico, two‐thirds of the ARGENT‐design positions convert to beneficial single mutants, validating the predictive power of the virtual Arg scan.

**FIGURE 2:**
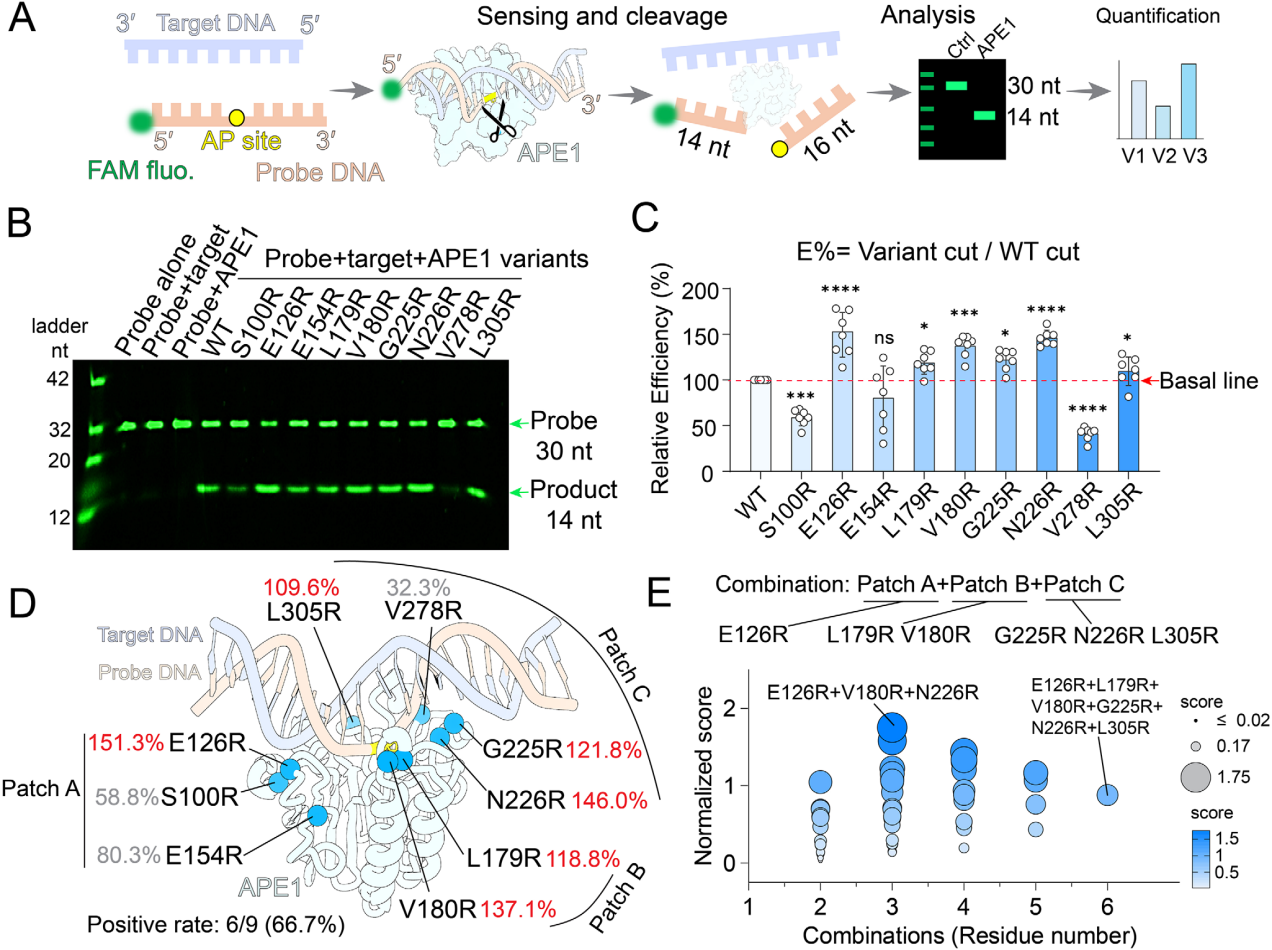
Experimental validation of AI‐guided APE1 variants and identification of an optimal triple mutant. (A) Schematic of the fluorescence probe assay used to monitor APE1 activity. A FAM‐labeled probe DNA containing a single AP site is hybridized to the complementary target DNA. Upon binding of APE1, the AP site is cleaved, generating a 14‐nt product that is separated by denaturing PAGE and quantified relative to the 30‐nt probe band. (B) Representative gel image showing probe cleavage by APE1 WT and the nine Arg‐scan variants. Reaction mixtures containing probe, target, and the indicated APE1 variant were incubated at 42°C for 30 min. The 30‐nt probe and 14‐nt product bands are indicated on the right, and a fluorescence ladder (12–42 nt) is shown on the left. (C) Relative cleavage efficiencies of APE1 variants compared to WT from 9 independent experiments. Bar graph shows E% = (variant cut / WT cut) × 100 at 30 min. The dashed red line indicates the WT level (100%) as the basal line. Data are presented as mean ± s.d. with individual data points overlaid. Statistical significance versus WT is indicated as ^****^ *p* <0.0001, ^***^ *p* <0.001, ^**^ *p* <0.01, ^*^ *p* <0.05, ns not significant (statistical tests described in Methods). (D) Structural mapping of single‐site variants on the APE1–DNA complex. The nine engineered positions are shown as spheres and colored by relative activity; the positive corresponding E% values are labeled in red. Patches A–C denote three spatially distinct DNA‐contact regions defined by the structural analysis. Six of nine variants (E126R, L179R, V180R, G225N, N226R and L305R) show ≥110% activity, yielding a positive rate of 6/9 (66.7%). (E) AI‐guided evaluation of multi‐site combinations across patches. Bubble plot shows normalized combination scores for all possible 2–6‐site combinations built from the six positive single mutants, grouped along the *x*‐axis by the number of residues in each combination. Bubble size encodes the predicted combination score (integrating single‐site hotspot scores, patch coverage, and a penalty for excessive mutation number), and color indicates normalized score. The triple mutant E126R+V180R+N226R, spanning patches A–C, exhibits the highest predicted score among all combinations and was selected as the lead APE1‐Evo variant for further characterization.

Mapping these activities back onto the structure showed that the six positive mutations segregate into three spatial patches around the AP site (Figure 2D). Patch A comprises E126R on one face of the lesion, Patch B contains L179R and V180R on the opposite helix, and Patch C encompasses G225N, N226R, and L305R further along the duplex. The most strongly activating residues—E126R, V180R and N226R—sit at the centres of these three patches (Figure 2D), suggesting that balanced reinforcement of multiple, distinct DNA‐contact regions may be more effective than overloading a single area with positive charge.

To guide higher‐order designs, we then used the ARGENT combination model to rank all Arg‐containing multi‐mutant constellations drawn from the nine sites, scoring each combination by its average hotspot strength, patch coverage and penalization for excessive substitutions. When plotted as a bubble map over combination size and residue identity (Figure 2E), this analysis highlighted E126R+V180R+N226R—linking the cores of Patches A, B and C—as one of the highest‐scoring triple mutants, with predicted performance exceeding any pairwise combination and approaching that of larger, more heavily charged designs. This provided a rational basis to focus subsequent biochemical characterization on combinations built from these three “core” residues.

### 2.3 AI‐Guided Patch Recombination Yields a Hyperactive APE1‐Evo

The single‐mutant analysis suggested that the most productive sites sit at the centres of three spatially separated DNA‐contact patches around the AP site‐E126 in Patch A, V180 in Patch B and N226 in Patch C (Figure 3A). We therefore used the ARGENT combination module to explore how best to recombine these three “core” residues. The model scores each multi‐mutant by averaging the single‐site hotspot scores, rewarding coverage of multiple patches and penalizing excessive local charge. This procedure highlighted four top‐ranked designs: three double mutants connecting pairs of patches (E126R+V180R, E126R+N226R, and V180R+N226R, denoted C1–C3) and one triple mutant linking all three patches (E126R+V180R+N226R, C4) (Figure 3B).

**FIGURE 3:**
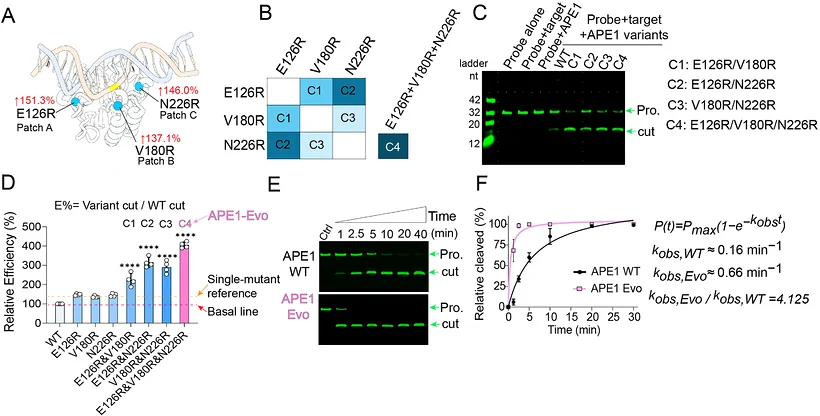
Patch‐based combination of hotspot mutations yields a hyperactive APE1‐Evo. (A) Spatial organization of the three key hotspot residues around the AP site. E126R (patch A), V180R (patch B), and N226R (patch C) are mapped onto the APE1–DNA complex and represented as cyan spheres. Their single‐mutant activities relative to WT are indicated (E126R, 151.3%; V180R, 137.1%; N226R, 146.0%), illustrating three distinct DNA‐contact patches surrounding the AP site. (B) Patch–combination matrix of double and triple mutants. The grid summarizes how the three hotspot residues are combined: C1 (E126R+V180R) links patches A and B, C2 (E126R+N226R) links patches A and C, C3 (V180R+N226R) links patches B and C, and C4 (E126R+V180R+N226R) spans all three patches simultaneously. (C) Representative fluorescence gel showing probe cleavage by APE1 WT and the four multi‐site variants. The 30‐nt probe (Pro.) and 14‐nt product (cut) bands are indicated on the right, with a fluorescence ladder on the left. (D) Quantification of cleavage efficiency for single and combined mutants. Relative efficiency is expressed as E% = (variant cut / WT cut) × 100 after 30 min. The dashed red line marks the WT basal line (100%), and the orange dashed line indicates the single‐mutant reference level. Bars show mean ± s.d. with individual data points overlaid. Statistical significance versus WT is shown as ^****^ *p* <0.0001, ^***^ *p* <0.001, ^**^ *p* <0.01, ^*^ *p* <0.05. The triple mutant E126R+V180R+N226R (APE1‐Evo, C4) reaches ∼4‐fold higher activity than WT. (E) Time‐course gel of probe cleavage under single‐turnover conditions. APE1 WT and APE1‐Evo (50 nm each) were incubated with 50 nm probe–target duplex at 42°C, and aliquots were quenched at the indicated time points. The progressive loss of probe and accumulation of product bands are shown for WT (black) and Evo (magenta). (F) Kinetic analysis of APE1 WT and APE1‐Evo. The fraction cleaved was plotted against time and fitted to a single‐exponential model $P\left(t\right)=P_{\max}\;\left(1-e^{-k_{\mathrm{obs}}t}\right)$. Under these conditions, APE1‐WT displays *k*_obs, WT_ ≈ 0.16 min^−1^, whereas APE1‐Evo displays *k*_obs, Evo_ ≈ 0.66 min^−1^, corresponding to a ∼4.1‐fold increase in cleavage rate (*k*_obs, Evo_/*k*_obs, WT_ ≈ 4.1).

We next compared these AI‐prioritized combinations experimentally in the same AP‐probe assay used for single mutants. There is no significant change in protein purity, suggesting that site combinations do not perturb protein folding (Figure S4A,B). All four combinations efficiently converted the 30‐nt probe into the 14‐nt product (Figure 3C). Quantitative analysis showed that each double mutant outperformed its constituent singles, with C1–C3 reaching roughly 200%–320% of wild‐type activity, whereas the triple mutant C4 achieved ∼380%–500% under identical conditions (Figure 3D; Figure S4C). Thus, ARGENT‐guided recombination of three spatial patches produces a strongly synergistic gain in apparent activity rather than simply additive effects. Notably, whereas wild‐type APE1 required approximately 20 min to reach near‐complete probe cleavage, C4 accomplished the same conversion within only ∼2.5 min (Figure 3E). Thus, ARGENT‐guided recombination of three spatial patches produces a strongly synergistic gain in apparent activity rather than simply additive effects.

To further assess kinetic consequences under single‐turnover conditions, we compared wild‐type APE1 and the triple mutant (hereafter APE1‐Evo) in time‐course assays at 50 nm enzyme and 50 nm DNA substrate (Figure 3E). Fitting the cleavage traces to a single‐exponential model $P\left(t\right)=P_{\max}\;\left(1-e^{-k_{\mathrm{obs}}t}\right)$ yielded similar endpoints but markedly different rate constants: *k*_obs, WT_ ≈ 0.16 min^−1^and *k*_obs, Evo_ ≈ 0.66 min^−1^, corresponding to a ∼4.1‐fold acceleration of AP‐site incision (Figure 3F). To further quantify the activity improvement of APE1‐Evo, we performed Michaelis–Menten kinetic analysis using AP‐containing DNA substrates at concentrations ranging from 10 to 400 nm. Initial velocities were calculated from the linear phase of the fluorescence curves for both APE1‐Evo and WT APE1. Compared with WT APE1, APE1‐Evo showed a lower apparent *K_m_* value and a slightly higher *V_max_*, resulting in an approximately 4‐fold increase in *V_max_*/*K_m_*. These results indicate that APE1‐Evo has enhanced apparent catalytic efficiency toward AP‐probe cleavage while retaining substrate‐dependent activity (Figure S5).

Consistent with this kinetic, APE1‐Evo also improved the analytical limit of detection by more than an order of magnitude in the AP‐probe cleavage readout, reducing the LOD from ∼5 fm for wild‐type APE1 to <500 am for APE1‐Evo (Figure S6). Together, these data show that AI‐guided patch‐level combination of ARGENT hotspots can convert a repair nuclease into a hyperactive variant, APE1‐Evo, while preserving robust catalytic behavior.

### 2.4 APE1‐Evo Retains High Specificity and Robust Activity Under Stringent Assay Conditions

Because APE1‐Evo shows increased activity, we next examined whether this acceleration compromises positional fidelity. To challenge this constraint, we designed four related target–probe pairs (a–d) that differ only at the AP‐opposite position, while keeping all other nucleotides constant (Figure 4A,B). Each probe strand carries a single abasic site and a 5′ FAM label, allowing cleavage to be monitored as conversion of the full‐length probe into a shorter product.

**FIGURE 4:**
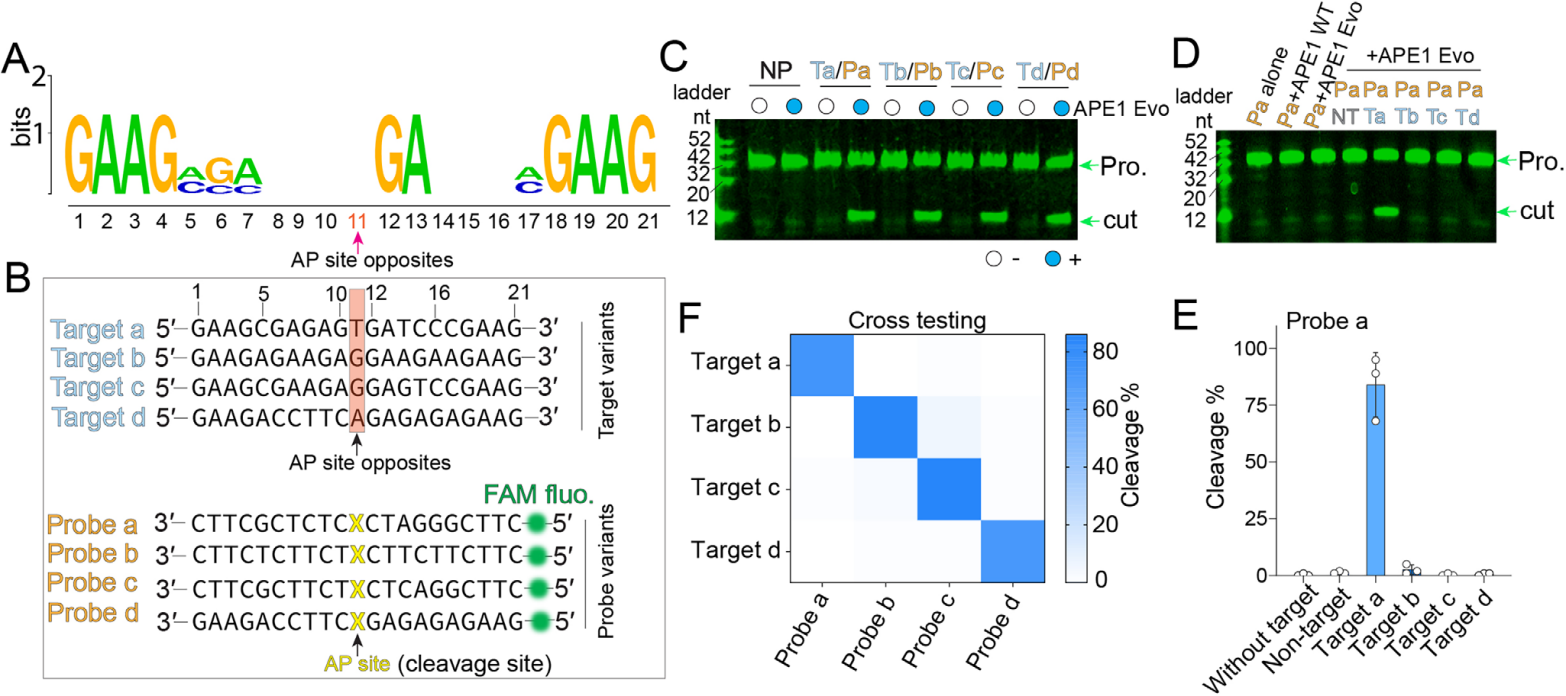
APE1‐Evo retains high mismatch discrimination at the AP site. (A) Sequence logo of designed nucleotides opposite the AP site derived from high‐throughput profiling. The AP‐opposite position (position 11) is indicated by the arrow, showing a strong preference for G opposite the lesion in the selected context. (B) Design of cognate and mismatched target–probe pairs. Four 21‐nt target strands (Targets a–d, blue) carry different bases at positions flanking the AP‐opposite site. Corresponding probe strands (Probes a–d, orange) contain a single AP site (X) at the cleavage position and are labeled with a 5′ FAM fluorophore. Probe a is fully matched to Target a, whereas the other combinations introduce defined mismatches around the AP site. (C) Cross‐pairing assay of APE1‐Evo with the different target–probe combinations. Denaturing PAGE shows cleavage of probe strands after incubation with APE1‐Evo and the indicated target strand. NP, non‐related probe control. Ta/Pa, Tb/Pb, Tc/Pc and Td/Pd denote cognate target–probe pairs. All matched pairs yield robust conversion of the 30‐nt probe (Pro.) to the 14‐nt product (cut), whereas non‐cognate combinations are barely cleaved. (D) Comparison of APE1 WT and APE1‐Evo on matched versus mismatched duplexes. Probe a (Pa) was annealed either to the cognate Target a or to non‐cognate targets (Tb–Td). Both APE1 WT and APE1‐Evo cleave efficiently only the perfectly matched Pa–Ta duplex, while duplexes containing mismatches at the AP‐opposite position or its neighbours show minimal product formation. (E) Quantification of cleavage specificity. Bar graph summarizes cleavage percentages for APE1‐Evo with Pa in the absence of target, in the presence of a non‐target strand, or annealed to each of the four targets. Efficient cleavage is observed only for the cognate Pa–Ta pair, confirming strict mismatch intolerance at the AP‐opposite site. Data are mean ± s.d. with individual points overlaid. (F) Heatmap of cross‐testing between all target and probe variants. Rows represent Targets a–d and columns represent Probes a–d. Color indicates the percentage of probe cleavage. A strong diagonal signal and near‐zero off‐diagonal values demonstrate that APE1‐Evo selectively cleaves only perfectly matched target–probe pairs, maintaining high sequence fidelity despite its enhanced catalytic activity.

Using APE1‐Evo, we first assayed all 4 target/probe combinations in a matrix format. Robust cleavage was observed only when each probe was paired with its perfectly matched target (Ta/Pa, Tb/Pb, Tc/Pc and Td/Pd), whereas all mismatched pairs showed minimal or no product band (Figure 4C). We then compared APE1‐Evo more directly using probe a. Both enzymes cleaved efficiently in the presence of the cognate target (Target a) but showed negligible activity with non‐target DNA or with the three mismatched targets (Targets b–d) (Figure 4D,E). The complete 4 × 4 cross‐testing summary confirms this behavior: strong signals are strictly confined to the diagonal, demonstrating that even in its hyperactive form APE1‐Evo is only activated by the correctly paired target–probe combination and retains highly stringent discrimination at the AP‐opposite position (Figure 4F). Thus, despite its ∼4‐fold higher apparent rate constant, APE1‐Evo retains the strict requirement for a correctly paired base opposite the AP site, indicating that AI‐guided Arg scanning can boost catalytic output without eroding sequence discrimination at the critical sensing position for APE1.

To further test whether APE1‐Evo maintains specificity in a more complex nucleic‐acid background, we purified *E. coli* genomic DNA and added it to the reaction at a final concentration of 50 ng/µL (Figure S7A). In the presence of target, APE1‐Evo produced robust fluorescence activation with or without *E. coli* genomic DNA, whereas control reactions lacking APE1‐Evo or target remained at background levels (Figure S7B). Although the addition of genomic DNA caused a slight delay in signal accumulation, the endpoint signal‐to‐noise ratio at 60 min was not significantly affected (Figure S7C). These results indicate that excess bacterial genomic DNA does not induce detectable nonspecific probe cleavage or compromise target‐dependent activation, further supporting the high fidelity of APE1‐Evo under complex nucleic‐acid background conditions.

### 2.5 APE1‐Evo Enables a Robust Multi‐Layer NAPTUNE‐V2.0 Cascade

Having established that APE1‐Evo is both hyperactive and sequence‐faithful, we next embedded it into an upgraded NAPTUNE architecture [28]. In NAPTUNE‐V2.0, a sensor probe P1 containing an internal AP site hybridizes to the target nucleic acid and is cleaved by APE1‐Evo to release a 14‐nt product; this product then programs *Pf*Ago to cut a quencher‐labeled booster duplex P2 carrying a second AP site, whose product in turn seeds a third booster probe P3, resulting in stepwise amplification of fluorescence (Figure 5A). Thus, APE1‐Evo operates at the entry point of a three‐layer APE1–*Pf*Ago cascade.

**FIGURE 5:**
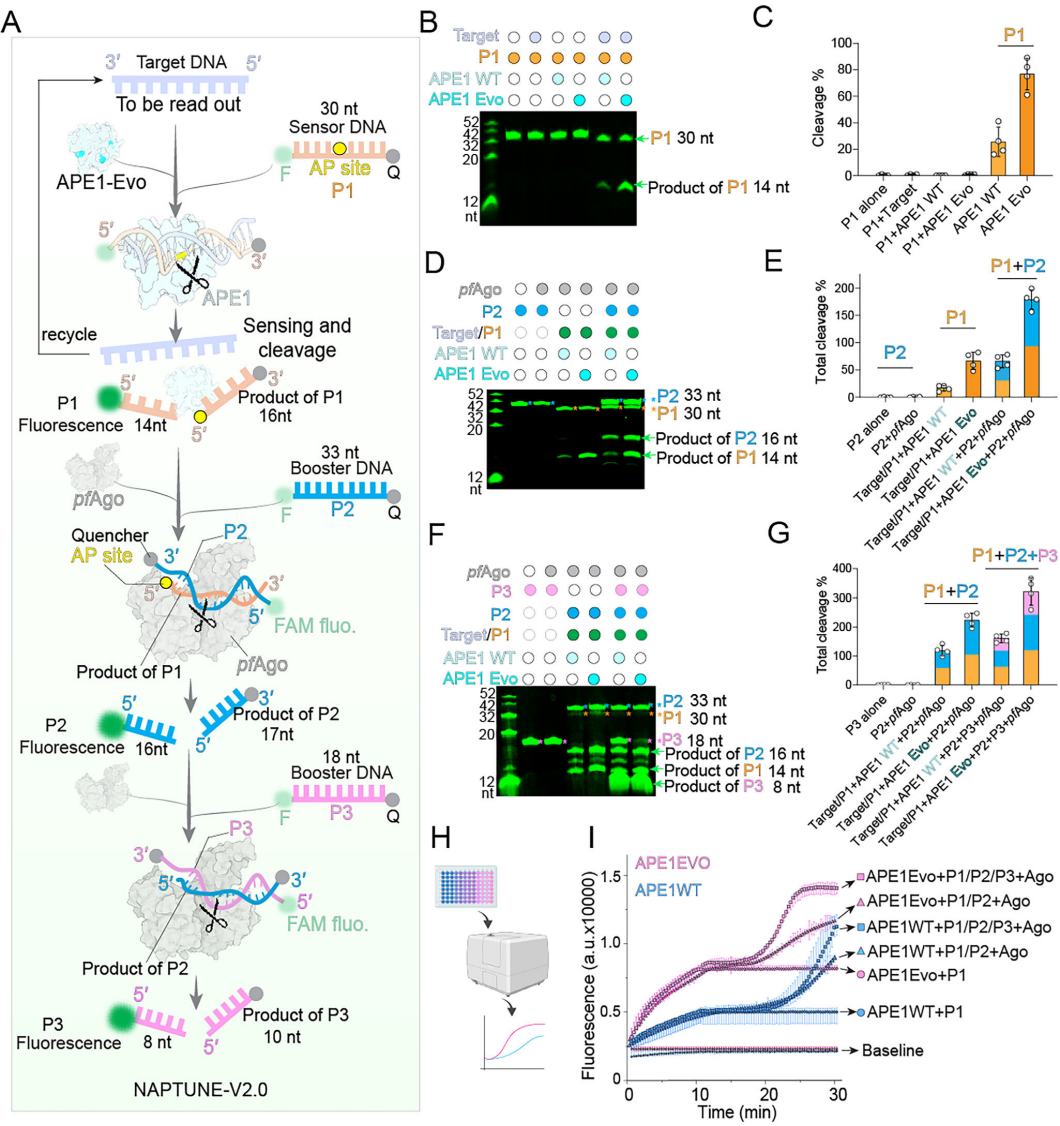
Upgraded NAPTUNE‐v2.0 platform using APE1‐Evo and multi‐layer *Pf*Ago coupling. (A) Schematic of NAPTUNE‐V2.0. Target DNA is first recognized by a 30‐nt sensor strand (P1) containing a single AP site and a fluorophore–quencher (F‐Q) pair. APE1‐Evo binds the probe–target duplex and cleaves at the AP site, generating a 14‐nt fluorescent product of P1 and a 16‐nt fragment that serves as a guide DNA for the second‐layer booster probe P2. In the second layer, *Pf*Ago cuts an AP‐containing P2 duplex to produce a fluorescent P2 fragment. A third APE1‐Evo/*Pf*Ago cycle further cleaves P3, yielding an 8–10‐nt fluorescent product. Iterative cleavage thus converts a single AP event into multiple fluorescence outputs (P1, P2, and P3). (B) Denaturing PAGE analysis of the first‐layer reaction. The 30‐nt P1 probe was incubated with or without target DNA and with APE1 WT or APE1‐Evo as indicated. Only in the presence of both target and APE1 is the 30‐nt probe converted to the 14‐nt product, with APE1‐Evo showing stronger cleavage than WT. (C) Quantification of P1 cleavage efficiency. Bar graph shows percentage of P1 cleaved under the conditions in (B). APE1‐Evo markedly enhances first‐layer signal compared with APE1 WT. Data are mean ± s.d. with individual points overlaid. (D) Second‐layer booster reaction combining APE1 and *Pf*Ago. P2 probes and their 33‐nt booster strands were added to the first‐layer reaction to generate an AP‐containing P2 duplex. Cleavage by APE1 and *Pf*Ago produces a 16‐nt P2 product and a 14‐nt P1 product. Gel shows that inclusion of APE1‐Evo and *Pf*Ago results in efficient conversion of both P1 and P2. (E) Quantification of total cleavage in the two‐layer system. Cleavage of P2 alone (blue) and combined P1+P2 products (orange) are plotted for the indicated enzyme combinations. The APE1‐Evo+*Pf*Ago cascade achieves the highest amplification. (F) Third‐layer booster reaction. Introduction of P3 probes and their booster strands onto the P1/P2 reaction enables a third round of AP‐site generation and cleavage. Gel analysis reveals accumulation of P1, P2, and P3 products in the presence of APE1‐Evo and *Pf*Ago, whereas control conditions show minimal signal propagation. (G) Quantification of three‐layer amplification. Bars depict total cleavage for P2 alone (blue) and combined P1+P2+P3 products (orange) across different enzyme conditions. The full cascade with APE1‐Evo yields the strongest multi‐step amplification. (H) Conceptual illustration of high‐throughput detection using NAPTUNE‐V2.0 in multi‐well format, where each well reports target DNA presence through cumulative fluorescence from P1 to P3. (I) Real‐time fluorescence readout of the NAPTUNE‐V2.0 system. Time‐course curves show fluorescence increase for APE1 WT or APE1‐Evo with P1 only, or with the full P1/P2/P3/*Pf*Ago cascade. APE1‐Evo combined with the three‐layer booster system produces the fastest and highest fluorescence signal, demonstrating that the engineered enzyme substantially enhances sensitivity and dynamic range of NAPTUNE‐based DNA detection.

We first examined the primary sensing step. In the presence of the cognate target, APE1‐Evo converted the 30‐nt P1 probe almost quantitatively to the 14‐nt product, whereas wild‐type APE1 gave substantially weaker cleavage under identical conditions, and no product was detected in the absence of APE1 or target (Figure 5B). Quantification showed that P1 cleavage by APE1‐Evo approached ∼90%–100%, roughly doubling the efficiency achieved by the wild‐type enzyme in the same time window (Figure 5C). Introducing *Pf*Ago and the P2 booster duplex revealed the second amplification layer: only reactions containing both target and APE1 produced the expected 16‐nt P2 product and its *PfA*go‐generated fragments, with APE1‐Evo again yielding markedly higher total P2 cleavage than wild‐type APE1 (Figure 5D, E).

Finally, we assembled the full NAPTUNE‐V2.0 cascade with P1, P2 and P3. In gels, the complete system produced sequential disappearance of all three full‐length probes and accumulation of their shorter products only when target, APE1 and *Pf*Ago were present (Figure 5F), and total cleavage across P1+P2+P3 was highest in reactions driven by APE1‐Evo (Figure 5G). In the NAPTUNE‐V2.0 cascade, this improvement is further amplified downstream, because a more efficient P1 cleavage step generates more *Pf*Ago‐programming products, thereby promoting stronger P2 and P3 cleavage. Real‐time fluorescence measurements in a plate‐reader format showed that the combination of APE1‐Evo with the three‐layer booster cascade generated the steepest and largest signal increase, whereas systems lacking *Pf*Ago, using wild‐type APE1, or omitting target remained at baseline (Figure 5H,I). Together, these data demonstrate that ARGENT‐engineered APE1‐Evo not only accelerates the primary AP‐probe cleavage step but also substantially amplifies the performance of the entire NAPTUNE‐V2.0 detection pipeline.

In addition to signal amplification, we further evaluated the operational robustness of the NAPTUNE‐V2.0 enzymatic components. In a prolonged 10‐h reaction, APE1‐Evo rapidly generated a strong fluorescence signal in the presence of the cognate target, whereas mismatched or no‐target controls remained close to background throughout the extended monitoring period (Figure S8A). This result indicates that APE1‐Evo does not accumulate substantial nonspecific signals even under reaction times far longer than the intended diagnostic window. We also tested the tolerance of both APE1‐Evo and PfAgo to repeated freeze–thaw handling. APE1‐Evo retained target‐dependent probe‐cleavage activity after 5 or 10 freeze–thaw cycles, and PfAgo similarly maintained robust target‐dependent activity without obvious background increase after repeated freeze–thaw treatment (Figure S8B, C). These results suggest that the key enzymatic components of NAPTUNE‐V2.0 preserve both activity and specificity under prolonged reaction and repeated handling conditions.

### 2.6 NAPTUNE‐V2.0 Enables Serotype‐Resolved and Pan‐Dengue Detection Using APE1‐Evo

To test whether the APE1‐Evo–driven NAPTUNE‐V2.0 platform can be programmed for real viral targets, we turned to dengue virus (DENV), whose ∼11 kb positive‐strand RNA genome encodes structural proteins (C, prM, E) and non‐structural proteins NS1–NS5 [43, 44] (Figure 6A). We selected DENV because dengue is one of the major tropical infectious diseases of strategic public‐health concern in Singapore and across Southeast Asia. Its high regional relevance also provides a meaningful diagnostic context for evaluating the practical utility of NAPTUNE‐V2.0. Using alignments of representative strains from all four serotypes, we mapped regions within NS5 and the capsid (C) gene that either harbour serotype‐specific signatures or remain highly conserved. Sequence logos [45] of these windows revealed clear patterns: positions suitable for serotype calling showed distinctive base preferences for each serotype, whereas pan‐reactive windows displayed near‐complete conservation (Figure 6B–E, top).

**FIGURE 6:**
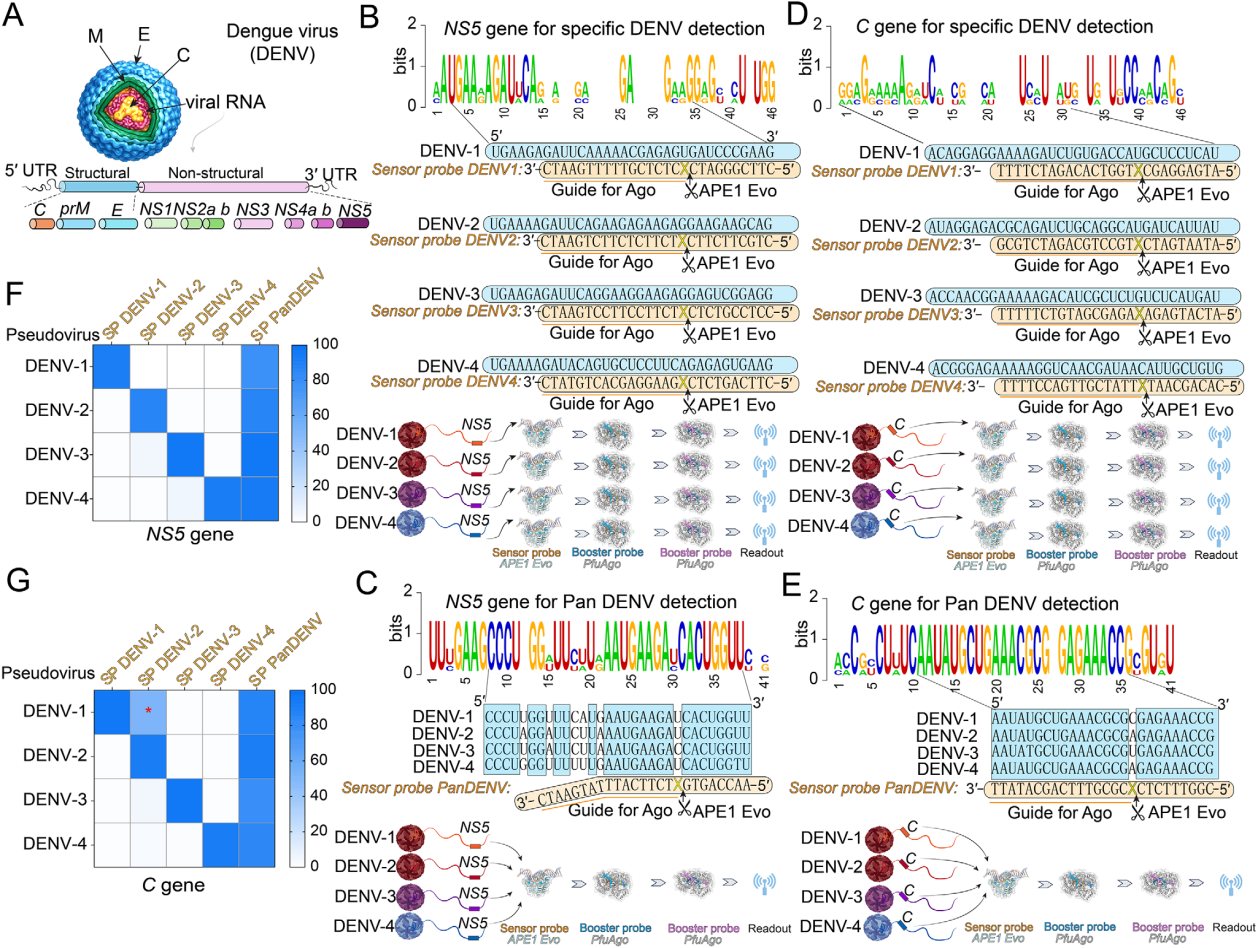
Application of NAPTUNE‐V2.0 to serotype‐specific and pan‐DENV detection using pseudoviruses. (A) Schematic illustration of dengue virus (DENV) virion and genome organization. The ∼11 kb positive‐strand RNA genome encodes structural proteins (C, prM, E) and non‐structural proteins (NS1–NS5). For nucleic‐acid sensing, conserved and serotype‐discriminating regions within the NS5 and C genes were selected as target sites for sensor design. (B) Design of serotype‐specific NS5 probes. Sequence logos (top) show nucleotide conservation across multiple DENV strains around the selected NS5 region. Alignments of DENV‐1 to DENV‐4 sequences (middle) highlight serotype‐specific signatures. For each serotype, a 30‐nt sensor probe (orange) containing a single AP site (X) is paired with a complementary NS5 fragment from the corresponding DENV pseudovirus. The 3′ portion of the sensor also serves as a guide for *Pf*Ago in the NAPTUNE‐V2.0 cascade (bottom). (C) Design of a pan‐DENV NS5 probe. A distinct NS5 segment with high conservation across all four serotypes was chosen (sequence logo, top). A single “PanDENV” sensor probe (orange) is complementary to this conserved region (middle) and functions as both APE1‐Evo substrate and *Pf*Ago guide (bottom), enabling detection of DENV‐1 to DENV‐4 with one probe. (D) Serotype‐specific probe design targeting the C gene. Sequence logos and alignments identify variable sites across the four DENV serotypes in the C coding region. Four C‐gene sensor probes (orange) are designed analogously to the NS5 probes in (B), each incorporating one AP site for APE1‐Evo and a PfAgo guide segment for downstream amplification. (E) Pan‐DENV C‐gene probe. A highly conserved stretch within the C gene is used to design a single pan‐DENV sensor probe (orange), which can detect all four serotypes while maintaining compatibility with the NAPTUNE‐V2.0 readout scheme. (F) Serotype specificity of NS5‐based detection using DENV pseudoviruses. Heatmap shows cleavage or fluorescence readout (0%–100%) obtained when each NS5 sensor probe (SP DENV‐1 to SP DENV‐4, and the pan‐DENV NS5 probe) is tested against RNA extracted from DENV‐1 to DENV‐4 pseudoviruses. Strong signal is observed predominantly on the diagonal, indicating high serotype specificity, whereas the pan‐DENV NS5 probe responds to all four serotypes. (G) Serotype specificity and pan‐reactivity of C‐gene‐based detection. Heatmap summarizes NAPTUNE‐V2.0 signals for C‐gene serotype‐specific probes (SP DENV‐1 to SP DENV‐4) and the pan‐DENV C probe across the four pseudoviruses. As with NS5, C‐gene probes exhibit minimal cross‐reactivity, and the pan‐DENV C probe generates robust signals for all serotypes, demonstrating that APE1‐Evo–powered NAPTUNE‐V2.0 can distinguish individual DENV serotypes while also enabling broad pan‐DENV detection.

For each serotype‐discriminating NS5 region, we designed a 30‐nt sensor probe (SP) containing a single AP site and a 3′ tail that doubles as a *Pf*Ago guide, yielding four “NS5‐SP DENV‐1–4” probes (Figure 6B). A separate conserved NS5 window was used to create one “PanDENV‐NS5” sensor capable of recognising all serotypes (Figure 6C). We repeated this design logic on the C gene to generate four serotype‐specific C probes and one pan‐DENV C probe (Figure 6D, E). In all cases, the AP site is positioned at the centre of the duplex to be cleaved by APE1‐Evo, while the 3′ segment feeds directly into the downstream *Pf*Ago amplification layer, keeping the detection chemistry uniform. Using synthetic RNA mimics of the NS5 and C target gene fragments, we first validated that APE1‐Evo can faithfully execute the intended readout logic for both the serotype‐specific and pan‐DENV probe sets. This step establishes a clean, enzyme‐level verification of probe performance before integrating the probes into the full NAPTUNE‐V2.0 cascade for pseudovirus‐based assays (Figures S9 and S10).

We next evaluated these probes using RNA extracted from DENV‐1 to DENV‐4 pseudoviruses. With NS5‐based sensors, each serotype‐specific probe produced a strong NAPTUNE‐V2.0 signal only with its cognate pseudovirus, whereas off‐diagonal combinations gave minimal responses (Figure 6F). This behavior is captured in the NS5 heatmap, where high signals are tightly confined to the diagonal for SP DENV‐1–4, demonstrating excellent serotype specificity. In contrast, the PanDENV‐NS5 probe responded robustly to all four pseudoviruses, confirming that a single AP sensor can support pan‐DENV surveillance (Figure 6F).

The C‐gene probes behaved similarly. Serotype‐specific C sensors again showed strong diagonal signals with negligible cross‐reactivity, while the pan‐DENV C probe yielded consistently high readouts for every pseudovirus tested (Figure 6G). Thus, without altering the core APE1‐Evo/*Pf*Ago chemistry, NAPTUNE‐V2.0 can be reprogrammed simply by changing the AP‐site sensor sequence to operate either in a serotype‐resolved mode or a broad pan‐DENV mode. These results establish that ARGENT‐engineered APE1‐Evo is compatible with complex viral genomes and that the NAPTUNE‐V2.0 framework readily generalizes from model targets to clinically relevant flaviviruses.

### 2.7 APE1‐Evo–Powered NAPTUNE‐V2.0 Enables Direct Detection of Influenza A and B Viruses in Clinical Samples

Having established performance on pseudoviruses, we next sought whether APE1‐Evo–driven NAPTUNE‐V2.0 can be directly applied to clinical respiratory specimens. Guided by multiple‐sequence alignments, we first identified short windows that are conserved within, but distinct between, major influenza A and B lineages [46, 47]. For influenza A (FluA), we selected a region in the matrix protein M1 gene that is preserved across H3N2, H5N1 and H7N9 strains, and designed a 30‐nt sensor probe “FluA” bearing a central AP site and a 3′ tail that serves as the *Pf*Ago guide (Figure 7A). For influenza B (FluB), an analogous design was built on a conserved stretch of the NS1 gene, yielding the “FluB” sensor probe (Figure 7B). In both cases, the AP position is placed opposite a well‐conserved base in the viral RNA so that APE1‐Evo reads out sequence identity at a single, information‐rich register.

**FIGURE 7:**
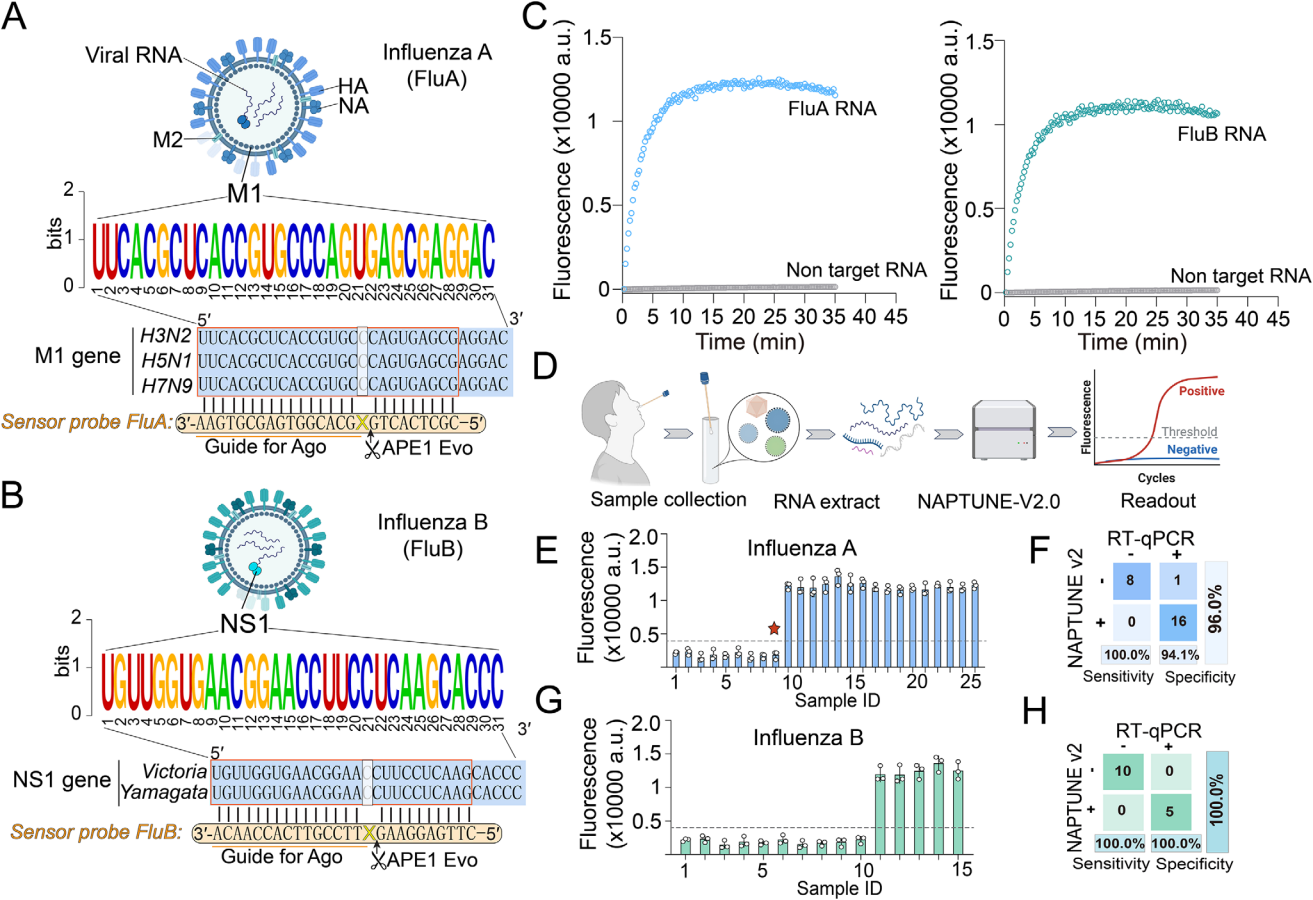
NAPTUNE‐V2.0 enables direct detection of influenza A and B viruses in clinical samples. (A) Design of an FluAsensor probe. Top, schematic of FluA virion and genome organization. A conserved region within the M1 gene was identified by sequence logo analysis (middle) across representative H3N2, H5N1, and H7N9 strains. Bottom, alignment of the M1 target sequences and the corresponding FluA sensor probe containing a single AP site (X) for APE1‐Evo and a 3′ segment that serves as a guide for *Pf*Ago in the NAPTUNE‐V2.0 cascade. (B) Design of a FluB sensor probe. A conserved stretch in the NS1 gene was selected based on multiple‐sequence alignment of Victoria and Yamagata lineages (sequence logo and alignment). The FluB sensor probe carries an AP site at the APE1‐Evo cleavage position and a *Pf*Ago guide region analogous to FluA. (C) Real‐time fluorescence detection of viral RNA by NAPTUNE‐V2.0. Left, fluorescence kinetics obtained when the FluA sensor probe is incubated with mimic FluA viral target RNA fragment (blue) or non‐target RNA. Right, equivalent assay for the FluB sensor probe with mimic FluB viral target RNA fragment or non‐target RNA. Robust and rapid fluorescence increases are observed only with cognate viral RNA, demonstrating high specificity. (D) Workflow for clinical testing. Nasopharyngeal swab samples are collected, viral RNA is extracted, and aliquots are subjected to NAPTUNE‐V2.0 reactions with the FluA or FluB sensor. Fluorescence is monitored over time and compared to a preset threshold to call samples positive or negative, in parallel with standard RT‐qPCR. (E) NAPTUNE‐V2.0 detection of FluA in clinical samples. End‐point fluorescence values for individual RNA extracts are shown, with the dashed line indicating the positivity threshold derived from healthy controls. Bars above the threshold are scored as FluA‐positive by NAPTUNE‐V2.0. The red star marks one sample that was RT‐qPCR‐positive but fell below the NAPTUNE‐V2.0 threshold, representing a discordant (false‐negative) call. (F) Performance summary of FluA detection. Contingency table comparing NAPTUNE‐V2.0 with RT‐qPCR for FluA clinical samples, with the corresponding sensitivity and specificity values indicated (see Methods for sample numbers and criteria). (G) NAPTUNE‐V2.0 detection of FluB in clinical samples. Fluorescence readouts for individual RNA extracts tested with the FluB sensor probe are plotted as in (E). All RT‐qPCR‐positive samples are clearly above the threshold, whereas RT‐qPCR‐negative samples remain at baseline. (H) Performance summary of FluB detection. Contingency table comparing NAPTUNE‐V2.0 calls with RT‐qPCR for FluB, demonstrating high sensitivity and specificity of the assay on real clinical specimens.

In purified RNA tests, both probes behaved as intended. When incubated with in vitro–transcribed viral mimic RNA at 42°C, the APE1‐Evo / *Pf*Ago cascade produced rapid fluorescence increases that reached plateaus within 35 min, whereas reactions containing non‐target RNA remained at baseline (Figure 7C). This confirms that the clinical FluA and FluB probe sets preserve the stringent target–non‐target discrimination previously observed for synthetic templates. We then established a workflow compatible with routine diagnostics (Figure 7D). Nasopharyngeal swabs from patients were processed with a standard extraction kit to obtain total RNA, which was split for parallel RT–qPCR (reference method) and NAPTUNE‐V2.0 analysis. For NAPTUNE‐V2.0, extracted RNA was directly added to a one‐pot reaction containing APE1‐Evo, *Pf*Ago, the corresponding AP sensor and booster probes, and fluorescence was monitored isothermally in a plate reader. A fixed threshold, defined from no‐template and RT–qPCR‐negative controls, was used to call positive versus negative samples.

In a cohort of FluA samples, NAPTUNE‐V2.0 generated clear binary signals (Figure 7E, Table S1). 16 RT–qPCR‐positive samples rose well above threshold, whereas 8 RT–qPCR‐negative samples stayed near baseline, yielding 16 true positives, 1 false negative (marked by the red star in Figure 7E), and no false positives (Figure 7F). Thus, for FluA our assay correctly identified 16 of 17 qPCR‐positive samples and all qPCR‐negative specimens, indicating high specificity and sensitivity that is mainly limited by a single borderline sample. For influenza B, performance was even closer to the RT–qPCR gold standard. All qPCR‐positive samples showed robust fluorescence increases and crossed the threshold, whereas every qPCR‐negative sample remained negative (Figure 7G), resulting in perfect concordance in the confusion matrix (10 true positives, 5 true negatives, no miscalls; Figure 7H). Importantly, these results were obtained without thermal cycling or reverse‐transcription–PCR instrumentation, relying instead on a single APE1‐Evo–*Pf*Ago isothermal reaction.

Together with the DENV pseudovirus data, these clinical tests demonstrate that ARGENT‐engineered APE1‐Evo can be seamlessly integrated into NAPTUNE‐V2.0 to support rapid, multiplexable detection of clinically relevant RNA viruses. The same enzyme and reaction logic can be reprogrammed for different pathogens simply by swapping AP‐site sensor probes, highlighting the practical potential of AI‐guided Arg scanning to convert a DNA repair nuclease into a broadly useful analytical module for nucleic‐acid diagnostics.

### 2.8 ARGENT‐Guided Arginine Scanning Improves the Activity of a Compact Cas9d Nuclease

After establishing APE1‐Evo and demonstrating its improved performance in the NAPTUNE‐V2.0 detection system, we next asked whether the ARGENT framework could be extended to another DNA‐binding nuclease scaffold. To provide an initial test of transferability beyond APE1, we selected *MG34‐1* Cas9d, a compact Cas9‐family nuclease with potential utility for genome engineering because of its reduced protein size compared with canonical Cas9 effectors [48, 49, 50, 51, 52] (Figure 8A).

**FIGURE 8:**
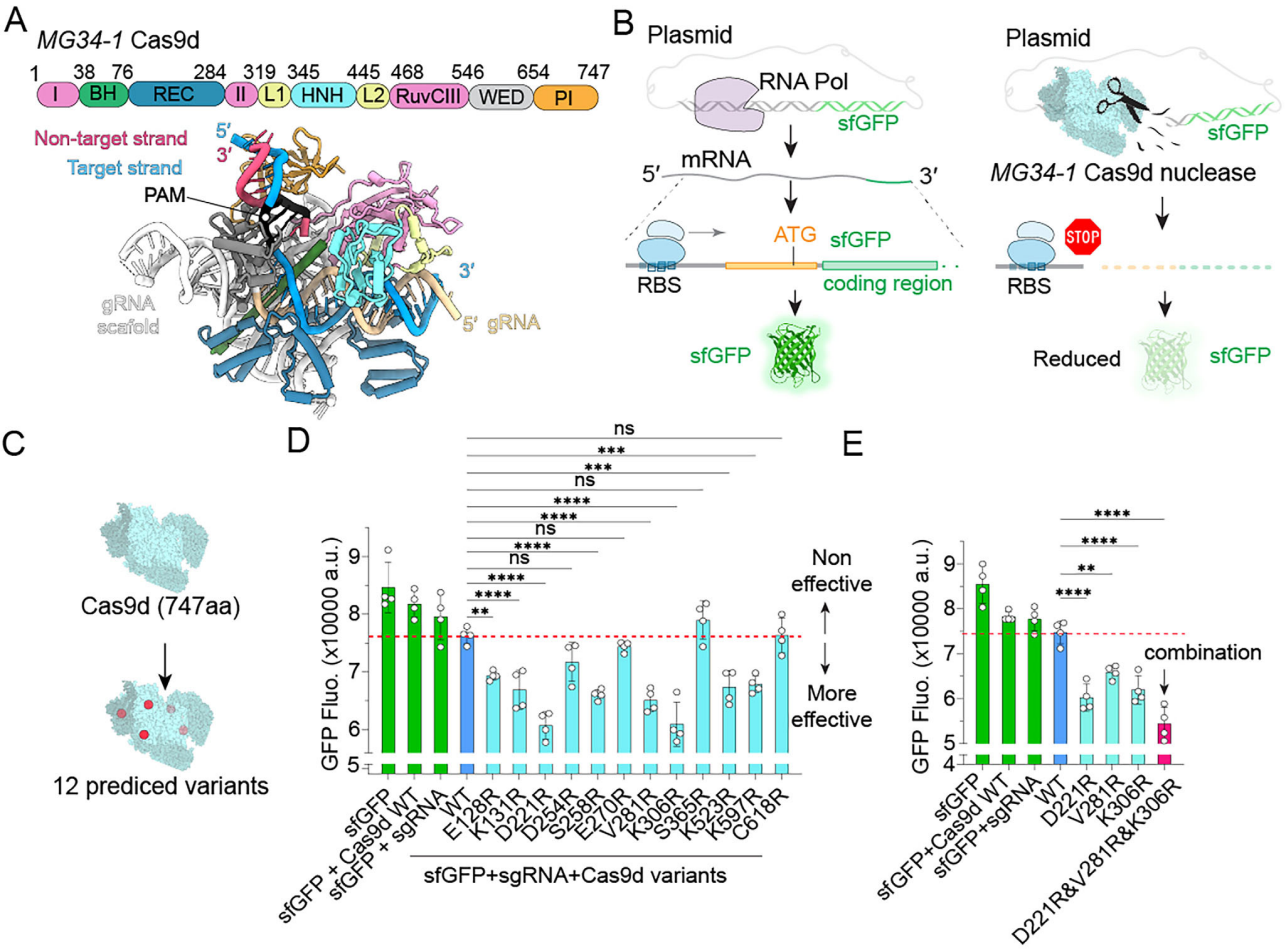
ARGENT‐guided arginine scanning improves Cas9d activity. (A) Domain organization and structural model of MG34‐1 Cas9d bound to guide RNA and target DNA. (B) Schematic of the sfGFP reporter assay used to evaluate *MG34‐1* Cas9d‐mediated DNA targeting. Active Cas9d targeting disrupts sfGFP expression, resulting in reduced fluorescence. (C) ARGENT‐guided selection of 12 predicted arginine‐substitution variants in Cas9d. (D) Screening of single Cas9d variants using the sfGFP reporter assay. Several ARGENT‐nominated variants showed reduced GFP fluorescence compared with parental Cas9d, indicating improved targeting activity. (E) Validation of top single mutants and the combined Cas9d variant. The D221R/V281R/K306R combination showed stronger activity than parental Cas9d, supporting the transferability of ARGENT‐guided arginine scanning to a distinct DNA‐binding nuclease. Data are shown as mean ± SD; ns, not significant; ^**^ *p* < 0.01; ^***^ *p* < 0.001; ^****^ *p* < 0.0001.

We designed a plasmid‐based sfGFP disruption assay to evaluate Cas9d activity. In this assay, the sfGFP expression cassette contains a Cas9d target site downstream of the T7 promoter and RBS. Successful Cas9d targeting of the sfGFP plasmid is expected to disrupt sfGFP expression, resulting in reduced fluorescence; therefore, lower GFP signal indicates stronger Cas9d‐mediated targeting activity (Figure 8B and Figure S11A, B). Using the ARGENT pipeline, we nominated 12 arginine‐substitution candidates distributed across the predicted Cas9d–nucleic‐acid interface for experimental testing (Figure 8C).

Screening of these single mutants revealed that most ARGENT‐selected variants reduced GFP fluorescence compared with parental Cas9d, indicating improved targeting activity (Figure 8D). Among the 12 tested variants, 9 showed enhanced activity relative to WT Cas9d, corresponding to a positive rate of 75%. Several mutations, including D221R, V281R, and K306R, produced clear activity improvements and were selected for further validation. We then combined these three productive substitutions to generate a triple mutant, D221R/V281R/K306R. This combined Cas9d variant showed a stronger reduction in GFP fluorescence than the parental enzyme and individual single mutants, corresponding to an approximately 30% improvement in activity based on the fluorescence assay (Figure 8E).

To further validate the reporter readout at the protein level, we analyzed sfGFP expression by SDS–PAGE. Consistent with the fluorescence measurements, Cas9d variants that showed stronger reporter disruption also led to reduced sfGFP protein abundance. Notably, the D221R/V281R/K306R combination produced a ∼5 folds stronger decrease in sfGFP band intensity than WT Cas9d, supporting enhanced disruption of sfGFP expression at the protein level (Figure S11C). Together, these results provide an initial demonstration that ARGENT‐guided arginine scanning can predict productive mutations in a distinct DNA‐binding nuclease scaffold. Although broader benchmarking will be required to define the full generalizability of the framework, the Cas9d validation supports the potential transferability of this computationally guided interface‐engineering strategy beyond APE1.

## 3 Discussion

In this study, we introduced ARGENT, an AI‐guided Arg‐scanning framework designed to provide a simple and general route for engineering nucleic‐acid binding proteins. Rather than building large, system‐specific models, ARGENT relies on minimal and interpretable inputs—a protein–DNA structure and a set of homologous sequences—to propose “virtual Arg” hotspots and productive charge patches. The same pipeline that we applied to APE1 can be run essentially unchanged on other DNA‐binding factors. As a first test of generality, we used ARGENT to analyse Cas9, identifying Arg‐tunable positions in a concise panel of mutants that showed improved on‐target editing in cells (Figure 8), providing initial evidence for broader applicability. These results indicate that ARGENT can act as a lightweight front‐end filter for many classes of nucleic‐acid‐binding proteins, including repair nucleases, restriction enzymes, recombinases, base editors and synthetic transcription factors, where traditional library‐based engineering may be costly or impractical.

APE1 provides a stringent testbed for ARGENT because it is a central base‐excision repair enzyme with a defined DNA‐binding interface, yet its substrate‐engagement surface had not been systematically optimized. ARGENT reduced 276 candidate surface positions to nine experimentally tractable sites, of which six Arg substitutions enhanced AP‐site cleavage. The final triple mutant, APE1‐Evo, achieved an approximately 4‐fold increase in apparent catalytic efficiency while retaining strict mismatch discrimination at the AP‐opposite position. Notably, a few predicted sites, such as S100R and V278R, reduced activity, indicating that interface proximity alone is insufficient and that local packing, steric compatibility, or substrate positioning can still generate false positives. Nevertheless, by prioritizing structurally plausible and evolutionarily tolerated positions, ARGENT produced a compact set of high‐value variants and supports controlled electrostatic reshaping as a practical strategy for improving DNA‐binding enzymes without broadly compromising fidelity.

The functional impact of this engineering is exemplified by the NAPTUNE‐V2.0 platform. By incorporating APE1‐Evo into a multi‐layer APE1–*Pf*Ago cascade, the enhanced AP‐probe cleavage activity is converted into stronger signal amplification for viral RNA detection. This upgraded architecture enables both serotype‐specific and pan‐reactive detection of dengue pseudoviruses, as well as accurate typing of influenza A and B from clinical nasopharyngeal RNA. Compared with classical CRISPR‐based detection platforms such as SHERLOCK and DETECTR, NAPTUNE‐V2.0 achieves sub‐femtomolar sensitivity without target amplification, highlighting its potential for simplified nucleic acid detection workflows (Table S2).

At the same time, both the method and the application have clear limitations. ARGENT, in its current form, is not a trained predictive model but a hand‐crafted scoring scheme: it uses static structures, simple geometric descriptors and heuristic weighting, without being fitted to any large mutational dataset. As such, it cannot explicitly account for conformational dynamics, DNA bending, long‐range electrostatics or subtle allosteric coupling, and it implicitly assumes that many beneficial variants can be approximated by Arg substitutions. Proteins whose specificity depends on finely tuned charge patterns or delicate hydrogen‐bond networks will therefore require more cautious interpretation and additional experimental vetting. Conservation constraints are likewise derived from conventional multiple‐sequence alignments and may be unreliable for proteins with sparse or biased homology profiles. In the future, high‐throughput mutational data across diverse DNA‐binding proteins could be used to train more expressive machine‐learning layers on top of ARGENT, automatically recalibrating feature weights, learning non‐Arg alternatives and system‐specifically correcting its biases. On the NAPTUNE side, our present implementation still relies on chemically synthesized AP probes, purified enzymes and fluorescence plate readers, which may limit immediate adoption in low‐resource settings, and our influenza cohort is modest and skewed toward relatively clear‐cut positives; larger, prospective studies will be required to fully define analytical sensitivity and robustness in complex clinical contexts. Finally, we have not yet evaluated APE1‐Evo in vivo, where increased DNA affinity could perturb endogenous base‐excision repair and will need to be carefully assessed before any cellular or therapeutic applications are considered.

These caveats point to clear directions for future work. Methodologically, ARGENT could be evolved from a purely heuristic score into a hybrid framework in which simple, interpretable features are retained but their weights and functional forms are refined by deep mutational scanning and larger training sets, and by incorporating richer structural descriptors and moving beyond Arg‐only designs. On the application side, NAPTUNE‐V2.0 could be coupled to simpler readouts, extended to broader multiplex pathogen panels and evaluated in larger prospective clinical cohorts. Overall, our results suggest that even a lightweight AI‐guided Arg‐scanning step can provide a practical entry point for evolving nucleic‐acid binding proteins and for turning housekeeping repair enzymes into modular catalytic units for next‐generation nucleic‐acid technologies.

## 4 Experimental Section

### 4.1 Materials

DNA oligos and fluorophore‐labeled probes were synthesized from General Biol (Anhui) Co., Ltd (Chuzhou, China), and sequences are listed in Supplementary Data. Plasmids for wild‐type, mutant APE1, and *Pf*Ago expression were supplied by Gene Universal Inc., Ltd (Newark, USA). ChamQ Universal SYBR qPCR Master Mix were obtained from Vazyme (Nanjing, China). FMR3 Real‐Time Quantitative Thermal Cycler was purchased from VAZYME BIOTECHNOLOGY SINGAPORE PTE LTD, Singapore. Ultrapure water was obtained from a Millipore Synergy system (MA, U.S.A.). All other chemicals and reagents were purchased from Sigma–Aldrich (MO, USA), Macklin Biochemical Co., Ltd (Shanghai, China), and Beyotime Biotechnology (Shanghai, China).

### 4.2 ARGENT Pipeline Overview

We developed ARGENT as an interpretable framework to score “virtual” Arg substitutions across DNA‐binding interfaces. The pipeline takes as input (i) a protein–DNA complex structure and (ii) residue‐wise conservation scores from a multiple sequence alignment. For each residue *i* in the protein, ARGENT computes a scalar virtual Arg hotspot score $H_{i}\in \left[0,1\right]$ that estimates how beneficial an Arg substitution at that position would be for DNA engagement, and then uses these scores to rank both single positions and multi‐site combinations. For APE1, we applied ARGENT to residues 43–318 of the APE1–DNA complex (total *L*  =  276 positions), treating the DNA phosphates as the primary electrostatic targets.

### 4.3 Structural Feature Calculation for Virtual Arg Substitutions

For every residue *i* (with Cα index i∈{1,…,L} in the 43–318 segment), we first compute a set of simple structural features that describe how a hypothetical Arg side chain at that position would be positioned relative to the DNA backbone.

### 4.4 Distance to DNA Phosphates

We define the distance feature *d_i_* as the minimum heavy‐atom distance between any atom of residue *i*and any phosphate atom in the DNA:

$$\;d_{i}=\min_{a\in i}\;\min_{p\in \mathrm{DNA}\;P}∥r_{a}-r_{p}∥$$

Distances are converted to a proximity score $D_{i}\in \left[0,1\right]$ using a soft cutoff *d*_cut_ (typically 10 Å):

$$D_{i}=\max\left(0,\;\frac{d_{\mathrm{cut}}-d_{i}}{d_{\mathrm{cut}}}\right)$$

Thus, residues closer to DNA phosphates (small *d_i_*) obtain higher *D_i_*, while residues beyond *d*_cut_get *D_i_* =  0.

### 4.5 Solvent Exposure and Local Packing

To approximate whether an Arg side chain could be accommodated at residue *i*, we compute a per‐residue solvent‐accessible surface area (SASA) *A_i_* using a rolling‐sphere algorithm with a 1.4 Å probe. The resulting areas are normalized to obtain an exposure score *E_i_*:

$$E_{i}=\frac{A_{i}}{\max_{j}A_{j}}$$

Residues with *E_i_* ≈ 1 are strongly surface‐exposed; buried residues have *E_i_* ≈ 0. This term disfavors introducing Arg at highly buried positions.

Optionally, a local packing descriptor *P_i_* can be defined as the number of Cα atoms within a radius *r*_pack_ (e.g. 8 Å) of residue *i*:

$$P_{i}=\left|\left\{j\left|j\neq i\right.,∥r_{\alpha ,i}-r_{\alpha ,j}∥\leq r_{pack}\right\}\right|$$

We then convert *P_i_* to a “crowding” factor $C_{i}\in \left[0,1\right]$ by

$$C_{i}=1-\frac{P_{i}}{\max_{j}P_{j}},$$

so that densely packed regions receive lower *C_i_*.

### 4.6 Raw Structural Score

We combine these structural terms into a raw per‐residue structural score $S_{i}^{\left(\mathrm{struct}\right)}$ as a weighted sum:

$$S_{i}^{\left(\mathrm{struct}\right)}=w_{D}\;D_{i}+w_{E}E_{i}+w_{C}C_{i},$$

where *w_D_*,*w_E_*,*w_C_* are scalar weights (for APE1 we set *w_D_* =  0.5, *w_E_* =  0.3, *w_C_* =  0.2). $S_{i}^{\left(\mathrm{struct}\right)}$ is then linearly rescaled to [0, 1]across all residues:

$${\tilde{S}}_{i}^{\left(\mathrm{struct}\right)}=\frac{S_{i}^{\left(\mathrm{struct}\right)}-\min_{k}S_{k}^{\left(\mathrm{struct}\right)}}{\max_{k}S_{k}^{\left(\mathrm{struct}\right)}-\min_{k}S_{k}^{\left(\mathrm{struct}\right)}}$$

### 4.7 Multi‐Scale Local Context Scoring

To capture the fact that residues act within local structural/sequence neighbourhoods, ARGENT computes a multi‐scale local context score around each position. For a given window half‐width *w*(in residues), we define the local mean of the raw structural score around residue *i* as

$${\bar{S}}_{i}^{\left(\mathrm{struct},w\right)}=\frac{1}{N_{i}^{\left(w\right)}}\;\sum_{j:\;|j-i|\leq w}{\tilde{S}}_{j}^{\left(\mathrm{struct}\right)}$$

where $N_{i}^{\left(w\right)}$ is the number of residues within the window that fall inside the 43–318 range.

We then normalize this local mean by the global average across the entire 43–318 segment:

$$\mu^{\left(\mathrm{struct}\right)}=\frac{1}{L}\;\sum_{k\;=\;1}^{L}{\tilde{S}}_{k}^{\left(\mathrm{struct}\right)}$$

$$L_{i}^{\left(w\right)}=\frac{{\bar{S}}_{i}^{\left(\mathrm{struct},w\right)}}{\mu^{\left(\mathrm{struct}\right)}}$$

A value $L_{i}^{\left(w\right)}>1$ indicates that residue *i*lies in a “hotter‐than‐average” region at scale *w*. For APE1, we used multiple window sizes (e.g. w∈{5,8,10,12,15,18}) to obtain a multi‐scale context vector

$$L_{i}=\left(L_{i}^{\left(5\right)},L_{i}^{\left(8\right)},L_{i}^{\left(10\right)},L_{i}^{\left(12\right)},L_{i}^{\left(15\right)},L_{i}^{\left(18\right)}\right).$$

We then condense this vector into a single context score $S_{i}^{\left(\mathrm{ctx}\right)}$ by taking a weighted average or maximum. In the APE1 application, we used a simple average:

$$S_{i}^{\left(\mathrm{ctx}\right)}=\frac{1}{M}\;\sum_{w}L_{i}^{\left(w\right)},$$

where *M*is the number of window sizes. $S_{i}^{\left(\mathrm{ctx}\right)}$ is again min–max normalized to [0, 1]over all residues.

### 4.8 Incorporation of Evolutionary Conservation

To reduce the risk of perturbing catalytic residues or structurally critical positions, ARGENT incorporates residue‐wise conservation scores *g_i_* from ConSurf‐like analyses, where *g_i_* ranges from 1 (variable) to 9 (highly conserved). We transform *g_i_* into a penalty factor $F_{i}\in \left[0,1\right]$ using a piecewise function:

$$F_{i}=\{\begin{array}{cc} 1, & g_{i}\leq g_{0},\\ \exp\left[-\alpha \left(g_{i}-g_{0}\right)\right], & g_{i}>g_{0}, \end{array}$$

where *g*_0_is a tolerance threshold and α > 0controls the steepness of the penalty. For APE1 we typically set *g*_0_ =  7and α  =  0.5, so that mildly conserved residues (*g_i_* ≤ 7) are effectively unpenalized, whereas highly conserved positions (*g_i_* =  8, 9) are progressively down‐weighted.

### 4.9 Virtual Arg Hotspot Score

The per‐residue virtual Arg hotspot score *H_i_* combines the normalized structural score, local context and conservation penalty:

$$H_{i}^{\left(\mathrm{raw}\right)}=\beta_{\mathrm{struct}}\;{\tilde{S}}_{i}^{\left(\mathrm{struct}\right)}+\beta_{\mathrm{ctx}}S_{i}^{\left(\mathrm{ctx}\right)}$$

$$H_{i}=F_{i}\;\cdot H_{i}^{\left(\mathrm{raw}\right)}$$

where β_struct_and β_ctx_are weights that balance local geometry versus neighbourhood information (for APE1 we used β_struct_ =  0.5, β_ctx_ =  0.5). *H_i_* is finally rescaled to [0, 1]by min–max normalization. Positions with higher *H_i_* are predicted to be better candidates for Arg substitution.

For visualizations (e.g. the “ARGENT landscape”), *H_i_* was plotted against residue index and/or mapped back onto the APE1 structure.

### 4.10 Patch Identification on the DNA‐Binding Surface

To extend from individual residues to spatial charge patches, ARGENT clusters high‐scoring positions on the protein surface.

We first define a seed set

$$S=\left\{i|H_{i}\geq H_{\mathrm{thr}}\right\},$$

where *H*_thr_ is a percentile‐based threshold (e.g. top 15%–20% of scores). Residues in S are then clustered in 3D space using single‐linkage clustering on Cα–Cα distances. Two residues *i*and *j*are assigned to the same patch if there exists a path $i=k_{0}\;,k_{1},\text{…},\;k_{n}=j$ such that

$$∥r_{\alpha ,k_{m}}-r_{\alpha ,k_{m+1}}∥\leq r_{\mathrm{patch}}$$

for all *m*, with a patch radius *r*_patch_typically set to 10 Å. This yields a set of patches $\left\{P_{1},P_{2},\text{…},P_{K}\right\}$, where each patch *P_k_* is a subset of residue indices.

For each patch *P_k_*, we compute a patch‐level score

$$\Phi_{k}=\frac{1}{|P_{k}|}\;\sum_{i\in P_{k}}H_{i}$$

Patches with larger Φ_*k*_represent more promising regions for introducing local positive charge.

### 4.11 Multi‐Site Combination Scoring

To prioritize multi‐residue designs, ARGENT ranks candidate combinations C⊆{1,…,L} using a simple scoring function that balances per‐residue quality, patch coverage and the number of substitutions.

Let ∣*C*∣be the number of residues in combination *C*, and let K(C) denote the set of distinct patches represented in *C*:

$$K\left(C\right)=\left\{k|P_{k}\cap C\neq \emptyset \right\}$$

We define:

- The average hotspot strength:
$$\bar{H}\left(C\right)=\frac{1}{|C|}\;\sum_{i\in C}H_{i}$$
- The normalized patch coverage:
$$\mathrm{cov}\left(C\right)=\frac{|K\left(C\right)|}{\min\left(|C|,K\right)}\;,$$

which is maximized when residues in *C* are drawn from as many different patches as possible.

- A substitution penalty that grows with combination size:
$$\mathrm{pe}n_{\mathrm{size}}\left(C\right)=|C|-1,$$

discouraging very large multi‐mutant designs.

The **combination score** is then

$$\mathrm{Score}\;\left(C\right)=\gamma_{H}\;\;\bar{H}\left(C\right)+\gamma_{\mathrm{cov}}\;\mathrm{cov}\left(C\right)-\gamma_{\mathrm{size}}\;\mathrm{pe}n_{\mathrm{size}}\left(C\right),$$

where γ_*H*_,γ_cov_,γ_size_ are scalar weights (e.g. γ_*H*_ =  1.0, γ_cov_ =  0.5, γ_size_ =  0.3 in the APE1 runs). Optionally, an intra‐patch crowding penalty can be added if multiple residues in *C* lie within a short distance cutoff on the same patch.

For APE1, we exhaustively enumerated all pairwise, triple, and (when computationally feasible) higher‐order combinations drawn from the top‐scoring residues, computed Score(*C*) for each, and ranked combinations accordingly. This analysis highlighted the triple mutant E126R+V180R+N226R (APE1‐Evo) as a high‐scoring design that spans three distinct patches with strong per‐residue hotspot scores and moderate combination size.

### 4.12 APE1 And PfAgo Proteins Expression and Purification

All APE1 proteins were expressed in Escherichia coli BL21 cells and purified with the following procedures. Briefly, Escherichia coli BL21(DE3) cells were transformed with the desired plasmid by heat shock. Then the transformant was inoculated in 10 mL Lysogeny Broth with appropriate antibiotics (50 µg/mL Kanamycin) and incubated at 37°C, 220 rpm overnight. After that, the small‐scale culture was added to 1.5 L Lysogeny Broth with antibiotics and incubated at 37°C until the OD_600_ reached 0.6‐0.8. Protein expression was then induced with 0.2 mm of isopropyl β‐D‐1 thiogalactopyranoside (IPTG) at 18°C overnight. The cells were collected by centrifugation (3500 × g, 15 min at 4°C) and resuspended at 4°C in lysis buffer (500 mm NaCl, 50 mm Tris pH 8.0, 10% Glycerol, 1 mm DTT) with 1 mm PMSF and 10 mm imidazole, followed by disruption with Ultrasonic Homogenizer. The crude cell lysate was centrifuged (16 000 × g, 40 min at 4°C) to remove the cell debris. The supernatant was then incubated with Ni‐NTA affinity beads for 30 min at 4°C. After that, wash the beads with 2CV Wash‐1 Buffer (20 mm imidazole in Lysis buffer) to remove the unwanted proteins and with 2CV Wash‐2 Buffer (1000 mm NaCl, 20 mm imidazole in Lysis buffer) to remove the unwanted nucleic acids. Finally, elute the beads with 1–2CV Elution Buffer (250 mm imidazole in Lysis buffer). The eluted sample was concentrated to around 1–2 mL using an Amicon Ultra filter unit (Millipore, 30 kDa). The sample was then loaded onto a Superdex 200 16/60 size‐exclusion column (Cytiva) equilibrated with SEC Running Buffer (200 mm NaCl, 25 mm Tris pH 8.0, 1 mm DTT) and were subsequently snap‐frozen and stored at −80°C.

*Pf*Ago was expressed in Escherichia coli BL21 cells and purified with the following procedures. Briefly, Escherichia coli BL21(DE3) cells were transformed with the desired plasmid by heat shock. Then the transformant was inoculated in 10 mL Lysogeny Broth with appropriate antibiotics (30 µg/mL chloramphenicol) and incubated at 37°C, 220 rpm overnight. After that, the small‐scale culture was added to 1.5 L Lysogeny Broth with antibiotics and incubated at 37°C until the OD_600_ reached 0.6–0.8. Protein expression was then induced with 0.2 mm of isopropyl β‐D‐1 thiogalactopyranoside (IPTG) at 18°C overnight. The cells were collected by centrifugation (3500×g, 15 min at 4°C) and resuspended at 4°C in lysis buffer (500 mm NaCl, 20 mm Tris pH 8.0, 1 mm DTT) with 1 mm PMSF and 10 mm imidazole, followed by disruption with an ultrasonic homogenizer. The supernatant of *Pf*Ago was collected, heated at 80°C for 15 min, and was centrifuged (16 000×g, 40 min at 4°C) to remove the denatured protein and cell debris. The supernatant was then incubated with Ni‐NTA affinity beads for 30–60 min at 4°C. After that, wash the beads with 2CV Wash‐1 Buffer (300 mm NaCl, 20 mm Tris pH 8.0, 1 mm DTT, 2 mm MnCl_2_, 50 mm imidazole) to remove the unwanted proteins and with 2CV Wash‐2 (1000 mm NaCl, 20 mm Tris pH 8.0, 1 mm DTT, 2 mm MnCl_2_) Buffer to remove the unwanted nucleic acids. Finally, elute the beads with 1–2CV Elution Buffer (1000 mm NaCl, 20 mm Tris pH 8.0, 200 mm imidazole, 1 mm DTT). The eluted sample was concentrated to around 1–2 mL using an Amicon Ultra filter unit (Millipore, 30 kDa). The sample was then loaded onto a Superdex 200 16/60 size‐exclusion column (Cytiva) equilibrated with SEC Running Buffer (150 mm NaCl, 20 mm HEPES pH 7.5, 1 mm DTT) and were subsequently snap‐frozen and stored at −80°C.

### 4.13 Denaturing Polyacrylamide Gel Electrophoresis

It was performed to analyze the cleavage of nucleic acids by APE1 and *Pf*Ago, as well as the evaluation of cleavage efficiency of NAPTUNE 2.0 platform. Reaction samples were mixed with formamide loading dye at a 1:3 ratio, heat‐denatured at 95°C for 5 min, and immediately loaded onto a 25% urea–polyacrylamide gel. Electrophoresis was performed at 160 V for 60 min in 0.5× TBE running buffer. Gels were imaged using a ChemiDoc MP imaging system (Bio‐Rad, Richmond, CA, USA).

### 4.14 APE1 Cleavage Assay and Cleavage Efficiency Comparison Between WT and Variants

Probes bearing a 5′‐FAM fluorophore and a 3′‐BHQ1 quencher and containing an AP site were annealed with their complementary target DNA strands to generate double‐stranded DNA substrates. Annealing was carried out by heating the mixture to 95°C for 3 min followed by gradual cooling to room temperature. Wild‐type APE1 and each mutant variant were diluted to a working concentration of 1 µM prior to use.

Cleavage reactions were assembled in a final volume of 10 µL containing probe 1 (50 nm), target DNA (50 nm), and either wild‐type or mutant APE1 (50 nm) in 1× APE1 cleavage buffer (20 mm Tris, pH 8.0; 525 mm NaCl; 5 mm MgCl_2_; 2 mm ATP; 0.1 mg/mL BSA). Reaction mixtures were incubated at 42°C for 30 min. Then, samples were quenched by addition of formamide loading dye at a 1:3 ratio, followed by heat denaturation at 95°C for 10 min. Cleavage products were subsequently resolved by denaturing polyacrylamide gel electrophoresis.

Fluorescent gel images were quantified using Fiji (ImageJ, version 1.54p; NIH, USA). Quantified values were analyzed in GraphPad Prism version 9.5 (GraphPad Software). For comparisons involving more than two groups, statistical significance was assessed using one‐way ANOVA followed by Dunnett's post‐hoc test. A *p*‐value < 0.05 was considered statistically significant.

### 4.15 Limit of Detection Analysis of Wild‐Type APE1 and Evo

Probes bearing a 5′‐FAM fluorophore and a 3′‐BHQ1 quencher and containing an abasic (AP) site were annealed with serially diluted complementary target strands to generate double‐stranded DNA substrates, as described above. Cleavage reactions were assembled in a total volume of 50 µL in 1× APE1 cleavage buffer (20 mm Tris, pH 8.0; 525 mm NaCl; 5 mm MgCl_2_; 2 mm ATP; 0.1 mg/mL BSA) containing probe (50 nm), wild‐type APE1 or Evo variant (50 nm), and target DNA at final concentrations of 500 am, 5, 50, 500 fm, 5, 50, 500 pm, 5, and 50 nm. Reaction mixtures were transferred into 96‐well plates and incubated on a CFX Opus 96 Real‐Time PCR System (Bio‐Rad) at 42°C for 90 min. Fluorescence was recorded at 0 and 90 min by monitoring FAM emission. Then, statistical analyses were performed in GraphPad Prism (version 9.5, GraphPad Software). Two‐way ANOVA with Dunnett's post‐hoc multiple‐comparison test was applied to evaluate differences across concentrations and enzyme variants. A threshold of *p* < 0.05 was considered statistically significant.

### 4.16 Tandem Cleavage Escalation Assays of APE1 and PfAgo in NAPTUNE 2.0

Prior to the cleavage reaction, the single‐stranded target and probe 1 were mixed at the appropriate ratio and annealed at 95°C for 3 min followed by slow cooling to room temperature to generate the double‐stranded substrate for APE1. A total reaction volume of 20 µL was prepared in 1× APE1 cleavage buffer (20 mm Tris, pH 8.0; 525 mm NaCl; 5 mm MgCl_2_; 2 mm ATP; 0.1 mg/mL BSA) containing probe 1 (200 nm), APE1 (100 nm), and the indicated concentrations of single‐stranded RNA or DNA targets. Reactions were incubated at 42°C for 30 min. The duration of this first step determines the amount of APE1‐derived DNA guides available for the subsequent PfAgo reaction.

Following APE1 reaction, *Pf*Ago (500 nm), probe 2, probe 3 (each 200 nm), and Mn^2+^ (5 mm) were added directly to the mixture. The reaction was then incubated at 88°C for 30 min to initiate *Pf*Ago‐mediated second‐ and third‐stage cleavage. Reaction products were analyzed by denaturing polyacrylamide gel electrophoresis.

Concentrations of individual components and incubation conditions can be adjusted depending on experimental requirements. *Pf*Ago exhibited robust activity under the described tandem‐cleavage conditions. For experiments assessing *Pf*Ago activity independently, reactions were performed in *Pf*Ago‐specific buffer (20 mm HEPES, pH 7.5; 250 mm NaCl; 5 mm MnCl_2_).

### 4.17 Real‐Time Fluorescence Measurements

Quantitative real‐time fluorescence measurements were used to monitor signal generation in the Tandem Cleavage Escalation assays of APE1 and *Pf*Ago within the NAPTUNE 2.0 platform, enabling a comparison of signal amplification between wild‐type APE1 and the Evo.4 variant. Reaction mixtures (50 µL; prepared as described in tandem cleavage escalation assays of APE1 and *Pf*Ago in NAPTUNE 2.0) were dispensed into 96‐well plates and analyzed on a CFX Opus 96 Real‐Time PCR System. Reactions were first incubated at 42°C for 10 min (20 s data‐acquisition interval). *Pf*Ago together with probe 2 and probe 3 were then added, followed by heating to 88°C for 20 min with continuous fluorescence recording at 20 s intervals. All RT‐qPCR experiments were carried out on an FMR3 Real‐Time Quantitative Thermal Cycler purchased from VAZYME.

### 4.18 Freeze‐Thaw Stability Assays of APE1‐Evo and PfAgo

To evaluate the handling stability of the enzymatic components used in NAPTUNE 2.0, purified APE1‐Evo and *Pf*Ago proteins were subjected to repeated freeze‐thaw treatment before activity measurement. For each freeze‐thaw cycle, protein were snap‐frozen in liquid nitrogen and then slowly thawed to room temperature. This procedure was repeated for 5 or 10 cycles. The treated proteins were then immediately used for real‐time fluorescence cleavage assays and compared with untreated controls.

For the APE1‐Evo freeze‐thaw stability assay, reactions were assembled in a final volume of 50 µL containing probe 1 (20 nm), target DNA (20 nm), and APE1‐Evo (20 nm) in 1× APE1 cleavage buffer (20 mm Tris, pH 8.0; 525 mm NaCl; 5 mm MgCl2; 2 mm ATP; 0.1 mg/mL BSA). Reaction mixtures were incubated at 37°C for 30 min, and FAM fluorescence was recorded in real time using a CFX Opus 96 Real‐Time PCR System.

For the *Pf*Ago freeze‐thaw stability assay, reactions were assembled in a final volume of 50 µL in 1× *Pf*Ago‐specific buffer (20 mm HEPES, pH 7.5; 250 mm NaCl; 5 mm MnCl2). Each reaction contained *Pf*Ago (100 nm), P2 probe (50 nm), and a synthetic DNA guide (50 nm) corresponding to the P2 guide sequence used in the cascade assay. The synthetic guide was phosphorylated at the 5′ end by T4 polynucleotide kinase before use. Reactions were incubated at 88°C for 30 min, and FAM fluorescence was monitored in real time.

Fluorescence data were analyzed using GraphPad Prism version 9.5. Signals from freeze‐thaw‐treated enzymes were compared with untreated enzyme controls to evaluate retained activity after repeated handling stress.

### 4.19 Long‐Duration Cross‐Reactivity Assay of APE1‐Evo

To evaluate whether non‐cognate target sequences could support sustained low‐level APE1‐Evo cleavage, a long‐duration cross‐reactivity assay was performed using one representative AP‐containing probe and four related target variants. Probe a was tested against its cognate Target a and non‐cognate Targets b, c, and d. Reaction mixtures were assembled in a final volume of 50 µL containing probe a (50 nm), the indicated target DNA (50 nm), and APE1‐Evo (50 nm) in 1× APE1 cleavage buffer (20 mm Tris, pH 8.0; 525 mm NaCl; 5 mm MgCl2; 2 mm ATP; 0.1 mg/mL BSA). Probe‐only and probe + APE1‐Evo reactions without target DNA were included as background controls.

Reactions were incubated at 37°C for 10 h, and FAM fluorescence was monitored in real time using a CFX Opus 96 Real‐Time PCR System. Fluorescence traces were used to compare cognate target‐dependent cleavage with non‐cognate target‐induced background signals. Data were analyzed using GraphPad Prism version 9.5 and plotted as mean ± s.d. from independent replicates.

### 4.20 E coli. Genomic DNA Background‐Challenge Assay

To assess whether nonspecific genomic DNA background affects APE1‐Evo‐mediated probe cleavage, an *E. coli* genomic DNA background‐challenge assay was performed. *E. coli* genomic DNA was extracted using the FastPure Bacteria DNA Isolation Mini Kit (Vazyme, Cat. DC103‐01) according to the manufacturer's instructions and added to the reaction at a final concentration of 50 ng/µL as a complex nonspecific DNA background.

Reaction mixtures were assembled in a final volume of 50 µL containing probe a (50 nm), target DNA where indicated (50 nm), and APE1‐Evo (50 nm) in 1× APE1 cleavage buffer (20 mm Tris, pH 8.0; 525 mm NaCl; 5 mm MgCl2; 2 mm ATP; 0.1 mg/mL BSA). *E. coli* genomic DNA was added where indicated.

Reactions were incubated at 37°C for 60 min, and FAM fluorescence was monitored in real time using a CFX Opus 96 Real‐Time PCR System. Endpoint signal‐to‐noise ratios were calculated from the 60‐min fluorescence values. The S/N ratio without genomic DNA was defined as F(probe + APE1‐Evo + target DNA) / F(probe + APE1‐Evo), and the S/N ratio with genomic DNA was defined as F(probe + *E. coli* gDNA + APE1‐Evo + target DNA) / F(probe + *E. coli* gDNA + APE1‐Evo). Data were analyzed using GraphPad Prism version 9.5 and plotted as mean ± s.d. from independent replicates.

### 4.21 Clinical Samples and Pseudovirus Information

Flu A‐ and Flu B‐positive and ‐negative respiratory specimens were previously collected and stored by The Children's Hospital of Zhejiang University School of Medicine during routine outpatient clinical care. These archived specimens were not specifically collected for the present study and were subsequently provided to us solely for ex vivo evaluation of the NAPTUNE‐V2.0 assay. Their use was approved by the Ethical Committee of The Children's Hospital of Zhejiang University School of Medicine (**2025‐IRB‐0420‐P‐01**). The sample identities, including their positive or negative status, were known in advance during assay arrangement; therefore, the clinical sample evaluation was not performed as a fully blinded study. However, the personnel conducting the NAPTUNE‐V2.0 assay were not informed of the sample identities during testing.

Due to biosafety restrictions, dengue virus (DENV) samples were prepared using pseudo typed viral particles as surrogates for assay evaluation. Commercial DENV pseudo virus particles (General Biol Co., Ltd., China) were thawed on ice and spiked into viral transport medium.

### 4.22 Viral Nucleic Acid Extraction

Total viral RNA was extracted from clinical samples (Flu A, Flu B) using the RaPure Viral RNA/DNA Kit (Magen, China) according to the manufacturer's protocol. Briefly, 200 µL of each sample was lysed and subjected to column‐based purification. Viral RNA was eluted in 40 µL of RNase‐free water and stored at −80°C prior to downstream analysis.

### 4.23 RT‐qPCR

The RNA templates of Flu A, Flu B, and DENV were extracted by RaPure Viral RNA/DNA kit and were converted into cDNA via TransScript Uni All‐in‐One First‐Strand cDNA Synthesis SuperMix for qPCR according to the manufacturer's instructions. qPCR was conducted using specific primers with the TB Green Fast qPCR Mix and was performed on StepOnePlus Real‐Time PCR System (Applied Biosystems, Foster City, CA, USA). The specificity of the primers was verified by melting curve analysis.

### 4.24 Detection of Viruses in Clinical Samples

Two microliters of extracted sample were first added into a pink tube containing 8 uL of APE1 reaction solution (20 mm Tris, pH 8.0; 525 mm NaCl; 5 mm MgCl_2_; 2 mm ATP; 0.1 mg/mL BSA, 100 nm of probe 1). 10 µL of *Pf*Ago reaction mixture (250 nm *Pf*Ago, 200 nm probe 2, 200 nm probe 3, 20 mm HEPES pH7.5, 250 mm NaCl, 2.5 mm MnCl_2_) was loaded into the mini tube. The tube was incubated at 42°C for 20 min for APE1 reaction and then heated to 90°C. After that, the *Pf*Ago reaction solution was dropped into the lower chamber by a brief spin. The mixed solution was incubated together at 90°C for 25 min. The fluorescence intensity was recorded by a microplate reader (Tecan Spark GmbH, Austria). The real‐time fluorescence signal was detected by the qPCR device (Bio‐Rad, CFX Connect Real‐Time System).

### 4.25 MG34‐1 Cas9d Screening Assay

Cas9d variants were evaluated using a cell‐free sfGFP interference assay. TXTL transcription‐translation reactions were performed using the GenScript CFXpress Cell‐free *E. coli* Protein Synthesis Kit (Cat. No. RP‐D00005‐1), a T7 promoter‐driven *E. coli* cell‐free protein synthesis system, according to the manufacturer's instructions with minor modifications. SUMO‐Cas9d variants were expressed from pET28_Cas9d plasmids under the control of a T7 promoter. The corresponding sgRNA was expressed from an IDT‐synthesized double‐stranded DNA fragment containing a T7 promoter.

For each reaction, a 20 µL cell‐free expression mixture was assembled by combining 6.6 µL Cell Extract, 10.4 µL Protein Synthesis Buffer, pET28_Cas9d plasmid template (10 ng/ul), and sgRNA‐expression dsDNA template (1 ng/ul). Reactions were incubated at 37°C for 2 h to allow expression of Cas9d and sgRNA. After this pre‐expression step, an sfGFP reporter plasmid encoding sfGFP under the control of a T7 promoter was added at a final amount of 3 ng. The reactions were then incubated at 37°C for an additional 90 min to allow Cas9d‐sgRNA‐mediated interference of sfGFP expression.

After the interference step, 5 µL of freshly mixed cell‐free reaction supplement, prepared by combining Cell Extract and Protein Synthesis Buffer at the same ratio used in the initial reaction, was added to support continued sfGFP expression and fluorescence development. FAM‐channel fluorescence was monitored at 37°C using an FMR3 Real‐Time Quantitative Thermal Cycler until the signal reached a steady plateau. Endpoint fluorescence values from the plateau phase were used for quantitative analysis. Each condition was tested with four parallel replicates.

Normalized interference efficiency was calculated from endpoint sfGFP fluorescence values using parental Cas9d WT + sgRNA as the baseline control. For each variant, the normalized interference efficiency was defined as: Normalized interference efficiency (%) = [F(WT Cas9d + sgRNA)—F(Cas9d variant + sgRNA)] / F(WT Cas9d + sgRNA) × 100, where F represents the endpoint FAM‐channel fluorescence signal from the plateau phase. Thus, positive values indicate enhanced sfGFP interference relative to parental Cas9d WT, whereas negative values indicate reduced interference activity. Data were analyzed using GraphPad Prism version 9.5.

### 4.26 Cell Culture and Transfection

HEK293T cells were cultured in DMEM (Sigma) supplemented with 10% fetal bovine serum (FBS; Gibco), 1% penicillin–streptomycin–glutamine (Gibco), and 1% minimum essential medium nonessential amino acids (Gibco) in a humidified incubator maintained at 37 °C with 5% CO_2_. For the detection of SpCas9 nuclease activities, as well as for the screening of their variants, HEK293T cells were seeded into 24‐well plates at 70%–80% confluence. After 12 h of incubation, 1.6 µg of plasmids were co‐transfected into the cells using polyethylenimine (PEI) according to the manufacturer's instructions. The plasmids included those encoding BFP–T2A–GFxxFP and the Cas9 systems in a molar ratio of 1:1. For genome editing assays, 1.6 µg of all‐in‐one plasmids encoding both the gRNA and nuclease were transfected. After 48 h, cells were analyzed by fluorescence‐activated cell sorting (FACS).

### 4.27 FACS Analysis

Before FACS analysis, cells were dissociated using 0.25% trypsin‐EDTA (Gibco) and resuspended in DMEM supplemented with FBS. To evaluate the activity of SpCas9 nuclease and screen their variants using the fluorescence reporter system, cells were analyzed for EGFP, mCherry, and BFP fluorescence. A total of 25 000 single‐cell events were recorded 48 h post‐transfection using a CytoFlex flow cytometer. Data analysis was performed with FlowJo X software (version 10.0.7).

### 4.28 Statistics and Reproducibility

All statistical analyses were performed using GraphPad Prism (version 9.5, GraphPad Software). For comparisons involving more than two groups, one‐way ANOVA followed by Dunnett's post‐hoc test was applied. Statistical significance was defined as *p* < 0.05. Information regarding data display and sample size is provided in the corresponding figure legends. For analyses involving clinical samples, sample sizes were not predetermined by statistical methods and experiments were not randomized.

## Author Contributions

C.H., T.H., Q.J., and S.L. conceived the project and overall strategy. J.W. designed and optimized the APE1 Evo and NAPTUNE assay workflows. J.W., T.W., F.W., X.Y.W., S.Z., S.M., Y.L., Z.Z., S.Y., K.L., Q.W., and Y. Wa. performed biochemical and detection experiments. T.W., Y. Wa., Y.Z., Q.J., and T.H. coordinated and carried out clinical sample collection and assays. B.W., F.Y., X. Wa., X.H., S.L., and C.H. performed computational, structural, and data analyses. J.W. and C.H. wrote the manuscript with input from all authors. Q.J., T.H., and C.H. supervised the research.

## Funding

This work was supported by the Singapore Ministry of Health NCID Programme for Research in Epidemic Preparedness and REsponse (PREPARE) [A‐8002743]; the National University of Singapore Presidential Young Professorship (PYP) [A‐8001195]; the Singapore Ministry of Education MOE Tier 1 grants [A‐8002032 and A‐8003977]; and MOE Tier 2 grants [A‐8003002 and A‐8003612]. T.H. is supported by the Natural Science Foundation of Zhejiang Province of China (Grant Nos. LQ23H200005 and LTGC24H200001) and the National Natural Science Foundation of China (Grant No. 22304157). National Natural Science Foundation of China (NSFC 32201059) for Y.Z., Q.J., supported for the structure analysis and clinical samples acquisition and transportation.

## Consent for Publication

No written consent for publication was obtained because no patient‐identifiable information or individual case details are included in this study. Only anonymized clinical specimens and general, non‐identifiable sample information were used for evaluation of the influenza virus nucleic acid detection assay.

## Conflicts of Interest

The authors declare no conflicts of interest. All relevant patent applications have been filed or otherwise addressed.

## Supporting information




**Supporting File 1**: advs76769‐sup‐0001‐SuppMat.pdf.

**Supporting File 2**: advs76769‐sup‐0002‐TableS1.xlsx.

**Supporting File 3**: advs76769‐sup‐0003‐TableS2.xlsx.

**Supporting File 4**: advs76769‐sup‐0004‐Data.zip.

## Data Availability

The data that support the findings of this study are available on request from the corresponding author. The data are not publicly available due to privacy or ethical restrictions.

## References

[1] T. Siggers and R. Gordan , “Protein–DNA Binding: Complexities and Multi‐Protein Codes,” Nucleic Acids Research 42, no. 4 (2014): 2099–2111, 10.1093/nar/gkt1112. PMC3936734 24243859

[2] H. C. Nelson , “Structure and Function of DNA‐Binding Proteins,” Current Opinion in Genetics & Development 5, no. 2 (1995): 180–189, 10.1016/0959-437X(95)80006-9. 7613087

[3] A. M. Edwards , A. Bochkarev , and L. Frappier , “Origin DNA‐binding Proteins,” Current Opinion in Structural Biology 8, no. 1 (1998): 49–53, 10.1016/S0959-440X(98)80009-2. 9519296

[4] G. Liu , Q. Lin , S. Jin , and C. Gao , “The CRISPR‐Cas Toolbox and Gene Editing Technologies,” Molecular Cell 82, no. 2 (2022): 333–347, 10.1016/j.molcel.2021.12.002. 34968414

[5] J. Y. Wang and J. A. Doudna , “CRISPR Technology: A Decade of Genome Editing Is Only the Beginning,” Science 379, no. 6629 (2023): add8643, 10.1126/science.add8643. 36656942

[6] P. Russomanno , P. Zizza , L. Cerofolini , et al., “Expanding the Functions of KHSRP Protein: Insights Into DNA G‐Quadruplex Binding,” Advanced Science 12, no. 8 (2025): 2410086, 10.1002/advs.202410086. PMC11848572 39763191

[7] C. Hu , H. Inoue , W. Sun , et al., “The Inner Nuclear Membrane Protein Bqt4 in Fission Yeast Contains a DNA‐Binding Domain Essential for Telomere Association With the Nuclear Envelope,” Structure 27, no. 2 (2019): 335–343.e3, 10.1016/j.str.2018.10.010. 30503780

[8] T. Hu , H. Feng , X. Ke , et al., “Self‐Phosphorylating DNAzyme DK1 Enables Programmable Multi‐Analyte Readout via PfAgo,” Biosensors and Bioelectronics 290 (2025): 117968, 10.1016/j.bios.2025.117968. 40916246

[9] X. Ke , H. Fan , J. Qu , et al., “PANDA: AND Logic‐gated RNA Sensing Enabled by a Photo‐activated RCA‐Argonaute Cascade,” Chemical Science 17, no. 21 (2026): 10643–10652, 10.1039/D6SC00827E. PMC13090041 42006480

[10] T. S. Wong , D. Zhurina , and U. Schwaneberg , “The Diversity Challenge in Directed Protein Evolution,” Combinatorial Chemistry & High Throughput Screening 9, no. 4 (2006): 271–288, 10.2174/138620706776843192. 16724918

[11] Y. T. Chai , C. M. Fang , Y. S. Lim , H. S. Loh , and C. F. Le , “Directed Evolution for the Discovery of Engineered Proteins and Small Peptides Using Molecular Mutagenesis,” ACS Synthetic Biology 14, no. 8 (2025): 2920–2938, 10.1021/acssynbio.4c00887. 40660468

[12] W. Hao , W. Cui , Z. Cheng , et al., “Development of a Base Editor for Protein Evolution via in Situ Mutation in Vivo,” Nucleic Acids Research 49, no. 16 (2021): 9594–9605, 10.1093/nar/gkab673. PMC8450078 34390349

[13] F. Wang , S. Ma , S. Zhang , Q. Ji , and C. Hu , “CRISPR Beyond: Harnessing Compact RNA‐guided Endonucleases for Enhanced Genome Editing,” Science China Life Sciences 67, no. 12 (2024): 2563–2574, 10.1007/s11427-023-2566-8. 39012436

[14] R. Guo , X. Sun , F. Wang , et al., “Engineered IscB‐ωRNA System With Improved Base Editing Efficiency for Disease Correction Via Single AAV Delivery in Mice,” Cell Reports 43, no. 11 (2024): 114973, 10.1016/j.celrep.2024.114973. 39541214

[15] D. Han , Q. Xiao , Y. Wang , et al., “Development of Miniature Base Editors Using Engineered IscB Nickase,” Nat Methods 20 (2023): 1029–1036. 10.1038/s41592-023-01898-9 37231266

[16] N. Xue , D. Hong , D. Zhang , et al., “Engineering IscB to Develop Highly Efficient Miniature Editing Tools in Mammalian Cells and Embryos,” Molecular Cell 84, no. 16 (2024): 3128–3140.e4, 10.1016/j.molcel.2024.07.007. 39096898

[17] R. Meng , J. Li , W. Wang , et al., “Engineered Cas12j‐8 is a Versatile Platform for Multiplexed Genome Modulation in Mammalian Cells,” Advanced Science 12, no. 33 (2025): 02593, 10.1002/advs.202502593. PMC12412624 40492429

[18] A. A. Anashkina , “Protein‐DNA Recognition Mechanisms and Specificity,” Biophysical Reviews 15, no. 5 (2023): 1007–1014, 10.1007/s12551-023-01137-7. PMC10643805 37974977

[19] R. L. Jernigan and I. Bahar , “Structure‐Derived Potentials and Protein Simulations,” Current Opinion in Structural Biology 6, no. 2 (1996): 195–209, 10.1016/S0959-440X(96)80075-3. 8728652

[20] W. S. Valdar , “Scoring Residue Conservation,” Proteins: Structure, Function, and Bioinformatics 48, no. 2 (2002): 227–241, 10.1002/prot.10146. 12112692

[21] H. Ashkenazy , E. Erez , E. Martz , T. Pupko , and N. Ben‐Tal , “ConSurf 2010: Calculating Evolutionary Conservation in Sequence and Structure of Proteins and Nucleic Acids,” Nucleic Acids Res 38 (2010): W529–533. 10.1093/nar/gkq399 PMC2896094 20478830

[22] N. M. Hoitsma , J. Norris , T. H. Khoang , et al., “Mechanistic Insight Into AP‐endonuclease 1 Cleavage of Abasic Sites at Stalled Replication Fork Mimics,” Nucleic Acids Research 51, no. 13 (2023): 6738–6753, 10.1093/nar/gkad481. PMC10359615 37264933

[23] L. Gros , A. A. Ishchenko , H. Ide , R. H. Elder , and M. K. Saparbaev , “The Major human AP Endonuclease (Ape1) Is Involved in the Nucleotide Incision Repair Pathway,” Nucleic Acids Research 32, no. 1 (2004): 73–81, 10.1093/nar/gkh165. PMC373275 14704345

[24] A. M. Whitaker , T. S. Flynn , and B. D. Freudenthal , “Molecular Snapshots of APE1 Proofreading Mismatches and Removing DNA Damage,” Nature Communications 9, no. 1 (2018): 399, 10.1038/s41467-017-02175-y. PMC5785985 29374164

[25] B. D. Freudenthal , W. A. Beard , M. J. Cuneo , N. S. Dyrkheeva , and S. H. Wilson , “Capturing Snapshots of APE1 Processing DNA Damage,” Nature Structural & Molecular Biology 22, no. 11 (2015): 924–931, 10.1038/nsmb.3105. PMC4654669 26458045

[26] L. Wang , W. Chen , C. Zhang , et al., “Molecular Mechanism for Target Recognition, Dimerization, and Activation of Pyrococcus Furiosus Argonaute,” Molecular Cell 84, no. 4 (2024): 675–686.e4, 10.1016/j.molcel.2024.01.004. 38295801

[27] L. Wang , X. Xie , F. Huang , et al., “An Engineered PfAgo With Wide Catalytic Temperature Range and Substrate Spectrum,” Advanced Science 12, no. 29 (2025): 2416631, 10.1002/advs.202416631. PMC12362761 40364725

[28] T. Hu , X. Ke , Y. Yu , et al., “NAPTUNE: Nucleic Acids and Protein Biomarkers Testing via Ultra‐sensitive Nucleases Escalation,” Nature Communications 16, no. 1 (2025): 1331, 10.1038/s41467-025-56653-9. PMC11790866 39900931

[29] Y. Wang , Z. Zhou , X. Wu , et al., “Pseudotyped Viruses,” Advances in Experimental Medicine and Biology 1407 (2023): 1–27. 10.1007/978-981-99-0113-5_1 36920689

[30] M. G. Guzman and E. Harris , “Dengue,” The Lancet 385, no. 9966 (2015): 453–465, 10.1016/S0140-6736(14)60572-9. 25230594

[31] N. Nanaware , A. Banerjee , S. Mullick Bagchi , P. Bagchi , and A. Mukherjee , “Dengue Virus Infection: A Tale of Viral Exploitations and Host Responses,” Viruses 13 (2021): 1967, 10.3390/v13101967. PMC8541669 34696397

[32] N. G. Iglesias and A. V. Gamarnik , “Dynamic RNA Structures in the dengue Virus Genome,” RNA Biology 8, no. 2 (2011): 249–257, 10.4161/rna.8.2.14992. 21593583

[33] Y. Bi , J. Yang , L. Wang , L. Ran , and G. F. Gao , “Ecology and Evolution of Avian Influenza Viruses,” Current Biology 34, no. 15 (2024): R716–R721, 10.1016/j.cub.2024.05.053. 39106825

[34] D. F. Tough , “Influenza's Signature Move,” Nature Immunology 19, no. 6 (2018): 518–520, 10.1038/s41590-018-0115-1. 29777225

[35] M. Liu , X. Zhao , S. Hua , et al., “Antigenic Patterns and Evolution of the Human Influenza A (H1N1) Virus,” Sci Rep 5 (2015): 14171. 10.1038/srep14171 PMC4585932 26412348

[36] R. E. Dumm and N. S. Heaton , “The Development and Use of Reporter Influenza B Viruses,” Viruses 11 (2019): 736. 10.3390/v11080736 PMC6723853 31404985

[37] M. J. Pekarek and E. A. Weaver , “Existing Evidence for Influenza B Virus Adaptations to Drive Replication in Humans as the Primary Host,” Viruses 15 (2023): 2032. 10.3390/v15102032 PMC10612074 37896807

[38] F. Jiang and J. A. Doudna , “CRISPR–Cas9 Structures and Mechanisms,” Annual Review of Biophysics 46, no. 1 (2017): 505–529, 10.1146/annurev-biophys-062215-010822. 28375731

[39] H. Wang , M. La Russa , and L. S. Qi , “CRISPR/Cas9 in Genome Editing and Beyond,” Annu Rev Biochem 85 (2016): 227–264. 10.1146/annurev-biochem-060815-014607 PMC13384723 27145843

[40] M. Jinek , F. Jiang , D. W. Taylor , et al., “Structures of Cas9 Endonucleases Reveal RNA‐Mediated Conformational Activation,” Science 343, no. 6176 (2014): 1247997, 10.1126/science.1247997. PMC4184034 24505130

[41] X. Cao , W. Fu , X. Li , F. Chen , and Y. Zhao , “DNA‐Encoded Fluorescence Signals for Imaging Analysis,” Small Methods 9, no. 6 (2025): 2401303, 10.1002/smtd.202401303. 40136076

[42] H. Summer , R. Gramer , and P. Droge , “Denaturing Urea Polyacrylamide Gel Electrophoresis (Urea PAGE),” Journal of Visualized Experiments 32 (2009): 1485, 10.3791/1485. PMC3329804 19865070

[43] S. K. Roy and S. Bhattacharjee , “Dengue Virus: Epidemiology, Biology, and Disease Aetiology,” Canadian Journal of Microbiology 67, no. 10 (2021): 687–702, 10.1139/cjm-2020-0572. 34171205

[44] M. Khanra , I. Ghosh , S. Khatun , N. Ghosh , and S. Gayen , “Dengue Virus‐host Interactions: Structural and Mechanistic Insights for Future Therapeutic Strategies,” Journal of Structural Biology 217, no. 2 (2025): 108196, 10.1016/j.jsb.2025.108196. 40090430

[45] G. E. Crooks , G. Hon , J. M. Chandonia , and S. E. Brenner , “WebLogo: A Sequence Logo Generator: Figure 1,” Genome Research 14, no. 6 (2004): 1188–1190, 10.1101/gr.849004. PMC419797 15173120

[46] C. P. Arevalo , M. J. Bolton , V. L. Sage , et al., “A Multivalent Nucleoside‐modified mRNA Vaccine Against all Known Influenza Virus Subtypes,” Science 378, no. 6622 (2022): 899–904, 10.1126/science.abm0271. PMC10790309 36423275

[47] J. Reina , “The Victoria and Yamagata Lineages of Influenza B Viruses, Unknown and Undervalued,” Revista Española de Quimioterapia 35, no. 3 (2022): 231–235, 10.37201/req/159.2021. PMC9134891 35180825

[48] J. Yang , T. Wang , Y. Huang , et al., “Insights Into the Compact CRISPR‐Cas9d System,” Nat Commun 16 (2025): 2462. 10.1038/s41467-025-57455-9 PMC11903963 40075056

[49] R. F. Ocampo , J. P. K. Bravo , T. L. Dangerfield , et al., “DNA Targeting by Compact Cas9d and Its Resurrected Ancestor,” Nat Commun 16 (2025): 457. 10.1038/s41467-024-55573-4 PMC11706934 39774105

[50] D. S. Aliaga Goltsman , L. M. Alexander , J. L. Lin , et al., “Compact Cas9d and HEARO Enzymes for Genome Editing Discovered From Uncultivated Microbes,” Nature Communications 13, no. 1 (2022): 7602, 10.1038/s41467-022-35257-7. PMC9755519 36522342

[51] K. Wang , J. Wang , X. Yang , W. Sun , G. Sheng , and Y. Wang , “Structural Insights Into Type II‐D Cas9 and Its Robust Cleavage Activity,” Nat Commun 16 (2025): 7396. 10.1038/s41467-025-62128-8 PMC12339737 40790018

[52] F. Wang , R. Guo , S. Zhang , et al., “Structural Insight Into IscB's RNA‐lid‐Based Inactivation Mechanism,” Nature Structural & Molecular Biology 33, no. 4 (2026): 603–614, 10.1038/s41594-026-01761-3. PMC13095665 41882346

