# Supplementary material for: Computation and Structure‐Guided Arginine Scanning Engineers a Hyperactive AP Endonuclease for Multiplex Viral RNA Sensing
Source: Adv Sci (Weinh). 2026 Jul 24;13(58):e76769. doi: 10.1002/advs.76769 (PMC13398136; doi:10.1002/advs.76769)
Supplement: Supplementary file 1 [file ADVS-13-e76769-s004.pdf]

Computation and Structure-guided Arginine scanning engineers a hyperactive AP endonuclease for multiplex viral RNA sensing

Running title: AI-Guided APE1-Evo for Multiplex Viral Tests

Junlan Wang et.al.,

This PDF file includes:

Figures S1 to S11

Supplementary Table S1. Samples information

Supplementary Table S2. Comparison of NAPTUNE-V2.0 with representative CRISPR-based diagnostic platforms

Other supporting materials for this manuscript include the following:

Legends for Datasets S1 to S4

Supplementary Dataset S1. ARGENT multi-scale local hotspot scoring and ConSurf-adjusted ranking for APE1.

Supplementary Dataset S2. Distance–exposure and two-window local hotspot scores for APE1 Arg-scanning.

Supplementary Dataset S3. Uncropped gel images.

Supplementary Data S4. Preliminary data.

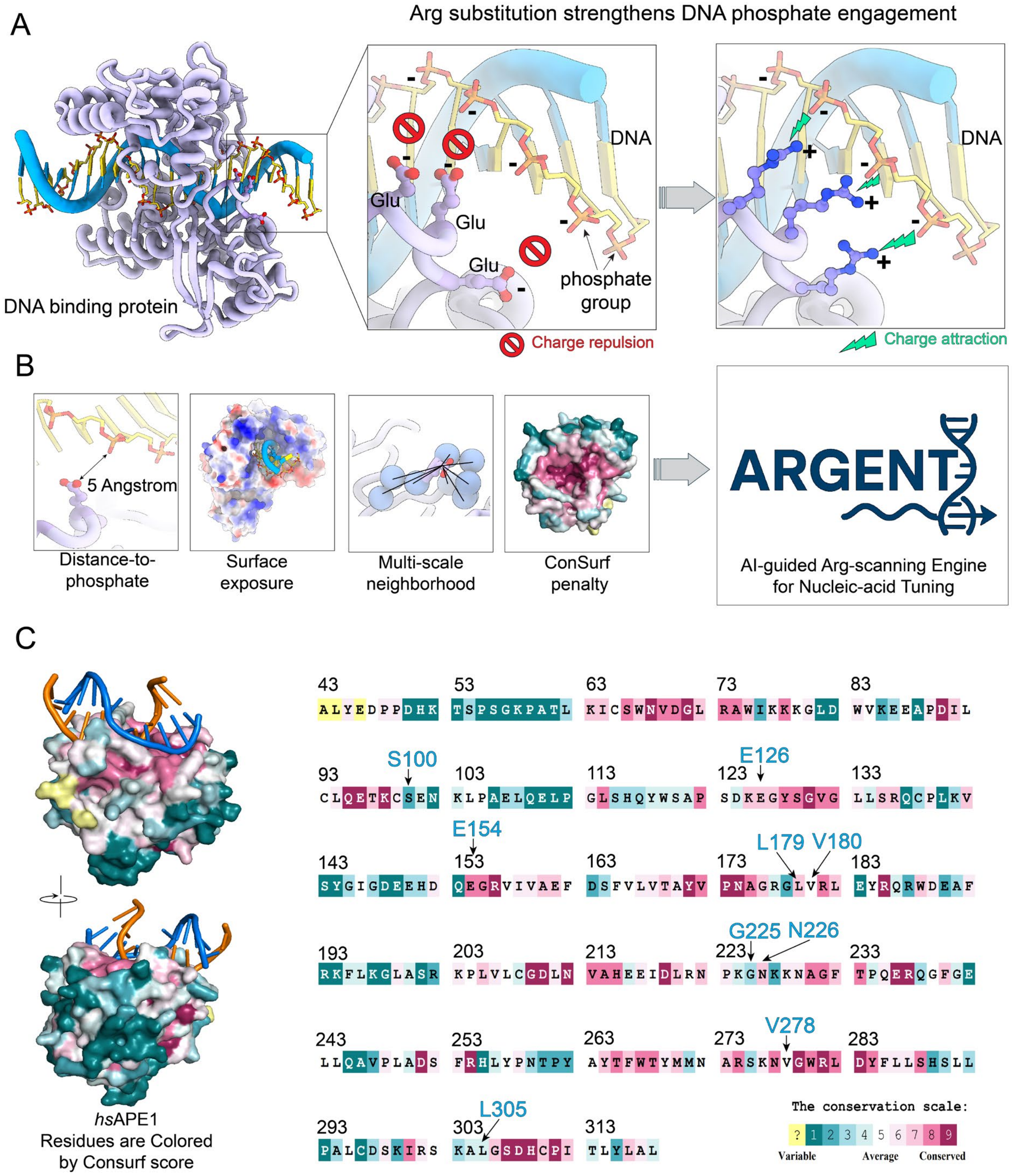

Supplementary Figure S1. Conceptual and feature-level overview of the ARGENT framework and conservation context of hsAPE1. (A) Conceptual illustration of charge-based interface tuning by Arginine substitution. Negative or neutral residues at DNA-contact surfaces can generate unfavourable electrostatic environments near the phosphate backbone(1). Converting selected positions to Arg is expected to introduce locally positive contacts, reduce charge repulsion, and strengthen phosphate engagement, thereby enhancing productive DNA binding and AP-site cleavage. **(B)** Schematic summary of the interpretable feature modules integrated in ARGENT (AI-guided Arginine-scanning Engine for Nucleic-acid Tuning). ARGENT evaluates residue-wise Arg-substitution potential by combining structural proximity to DNA phosphates, solvent-exposed accessibility, multi-scale neighbourhood context (capturing local

charge/geometry enrichment across different window sizes)(2), and evolutionary constraints derived from ConSurf(3). These components jointly define a ranked and filterable design space for DNA-interface optimization. **(C)** ConSurf-based conservation mapping of human APE1. Surface renderings (left) and the corresponding sequence view (right) depict residue-wise conservation grades derived from homologous sequence alignments. The nine experimentally tested ARGENT-selected positions (S100, E126, E154, L179, V180, G225, N226, V278 and L305) are indicated, illustrating that the selected sites are distributed across the DNA-contact surface while avoiding highly constrained residues, consistent with conservation-aware design principles.

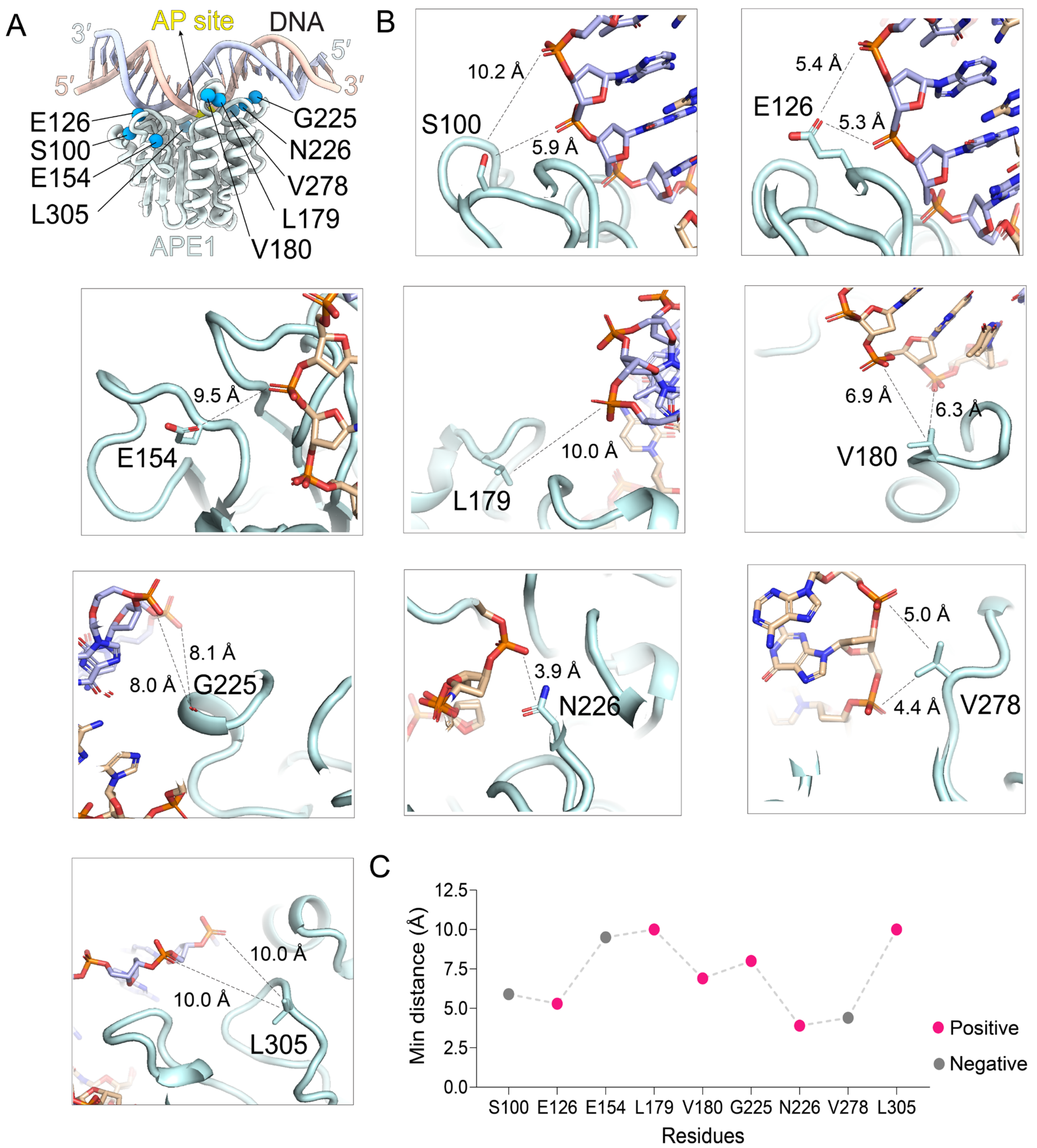

Supplementary Figure S2. Structural context of nine ARGENT-selected Arg-scan candidates on the APE1–DNA complex. (A) This figure provides a residue-level structural rationale for the nine candidate sites prioritized by ARGENT. The overview (upper left) maps S100, E126, E154, L179, V180, G225, N226, V278 and L305 on the APE1–DNA complex with the AP-site–containing duplex. **(B)** Individual zoom-in views highlight the spatial relationship between each side chain and the neighbouring DNA backbone and/or local electrostatic environment. The shortest distances to nearby DNA phosphates or nucleotides are indicated, illustrating that these candidates are positioned within interaction-competent ranges and distributed across multiple DNA-contact patches. Together, these structural snapshots support a plausible molecular basis for Arg substitution at these sites to reinforce phosphate engagement and contribute to enhanced AP-site cleavage, consistent with the ARGENT scoring and subsequent

biochemical validation. **(C)** Summary plot of the minimum distances from the nine ARGENT-selected residues to the nearest DNA phosphate. Residues are color-coded by experimental outcome in the single-mutant screen, illustrating that productive substitutions are enriched at positions with favorable DNA-proximal geometry.

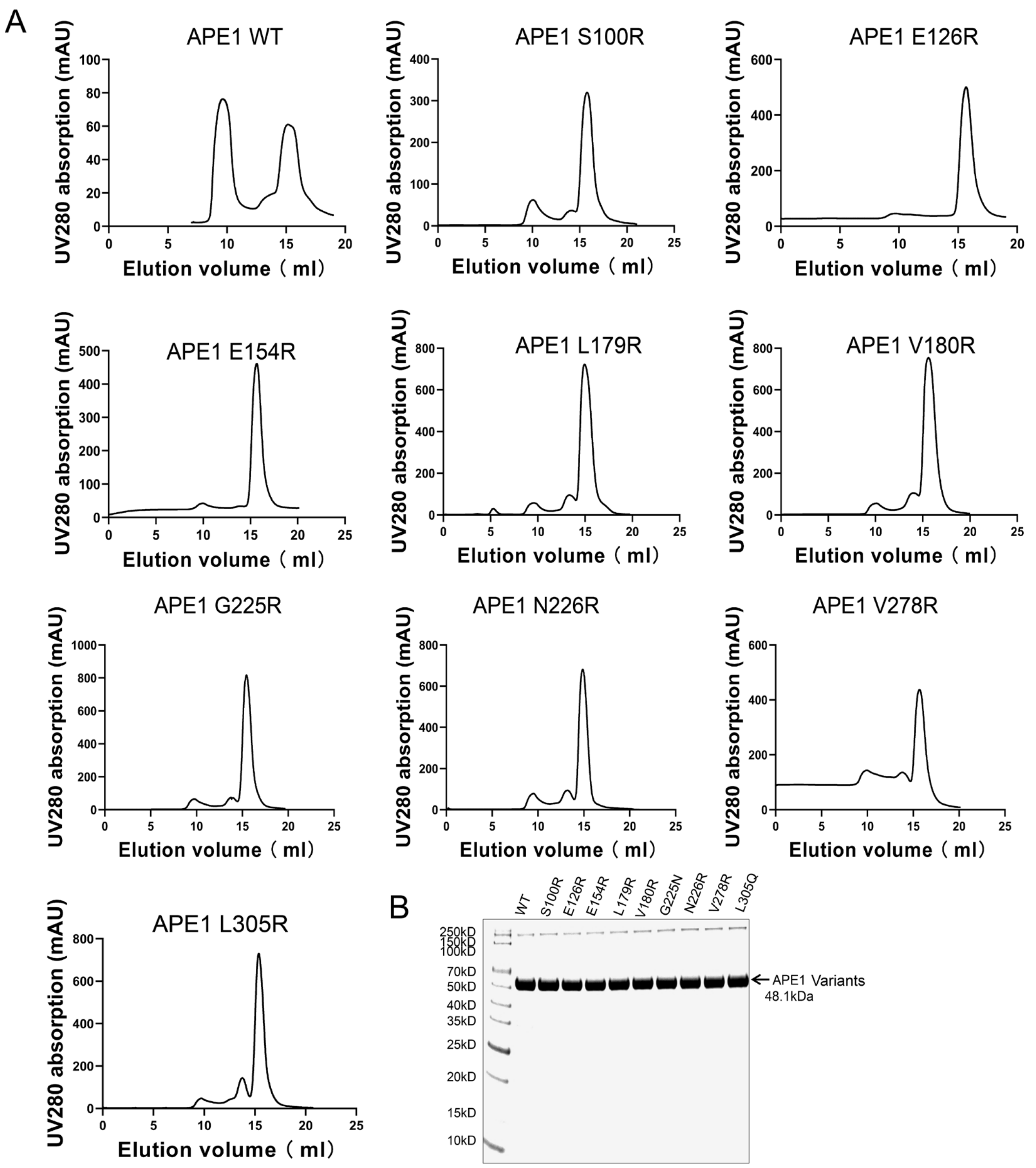

Supplementary Figure S3. Purification and quality assessment of APE1 Arg-scan variants. (A) Size-exclusion chromatography (SEC) profiles of APE1 WT and the nine single-Arg variants (S100R, E126R, E154R, L179R, V180R, G225R, N226R, V278R and L305R), monitored by UV280. The variants display dominant, well-defined elution peaks with broadly similar elution volumes, indicating that Arg substitutions do not grossly perturb protein folding or solution behavior and yield largely monodisperse preparations suitable for downstream biochemical assays. **(B)** SDS–PAGE analysis of purified APE1 WT and variants. All proteins migrate at the expected molecular weight (~48.1 kDa) with

comparable band intensities and minimal contaminating species, supporting consistent expression and purification across the Arg-scan panel.

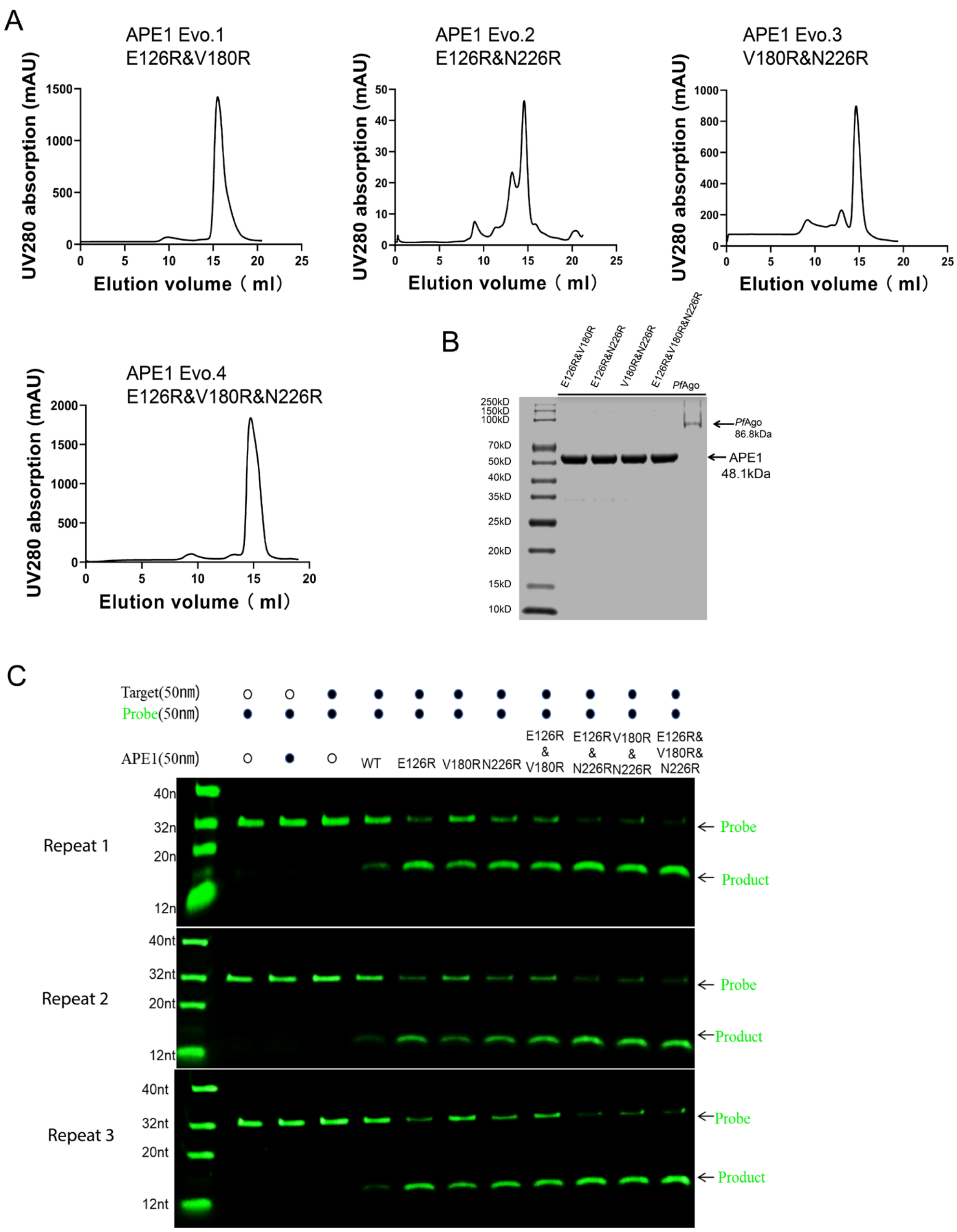

Supplementary Figure S4. Purification and activity validation of APE1 multi-site designs. (A) Size-exclusion chromatography (SEC) profiles of the top-ranked combinatorial variants prioritizing the three strongest single-hit sites. APE1 Evo.1 (E126R+V180R), Evo.2 (E126R+N226R), Evo.3 (V180R+N226R) and Evo.4 (E126R+V180R+N226R) each show a dominant, well-defined elution peak, indicating that the AI-guided combinations remain compatible with stable expression and soluble purification. **(B)** SDS–PAGE analysis of purified APE1 combinatorial variants. All

constructs migrate at the expected molecular weight (~48.1 kDa) with comparable purity. *Pf*Ago is shown as a reference control (~86.8 kDa). **(C)** Representative gel-based cleavage assays of APE1 WT, single mutants and AI-guided combinations under the indicated target/probe/enzyme conditions (50 nM each). Three independent repeats are shown. The appearance of the expected shorter cleavage product and the reduced full-length probe band confirm that multi-site designs retain robust AP-site processing, with the strongest signal enhancement observed for the triple mutant Evo.4 relative to WT and most pairwise combinations.

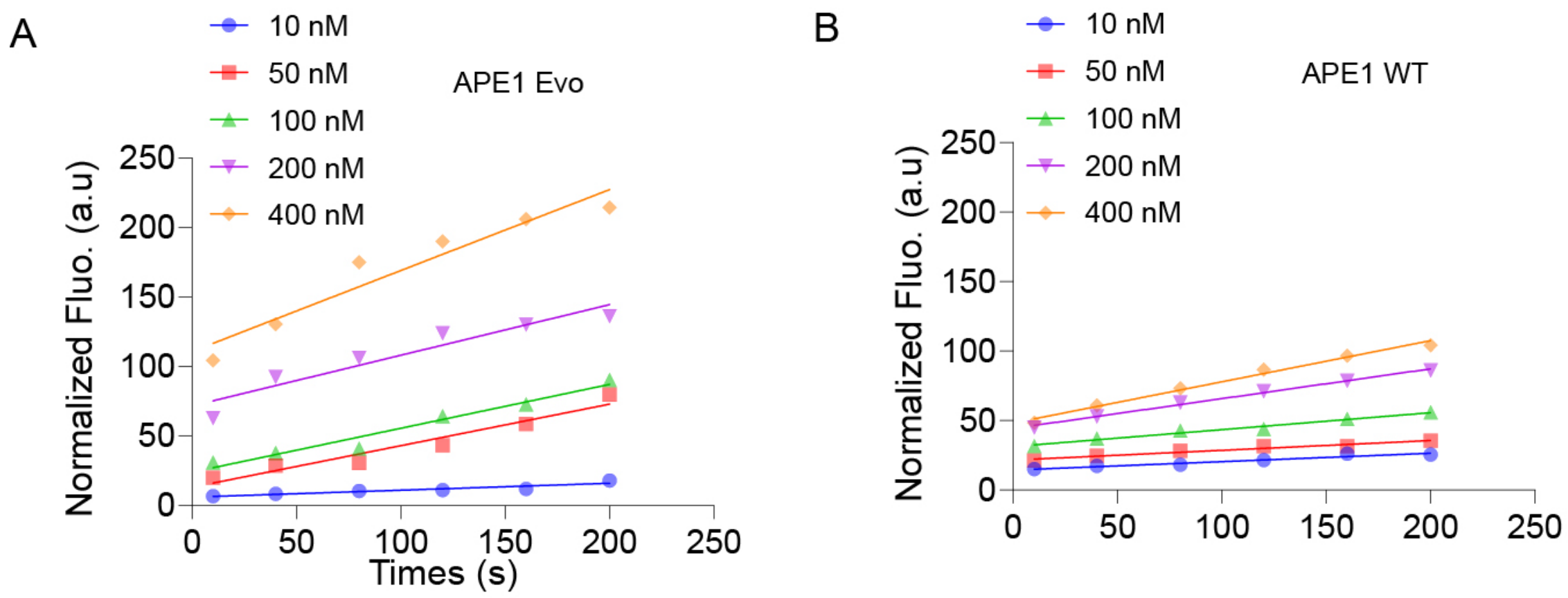

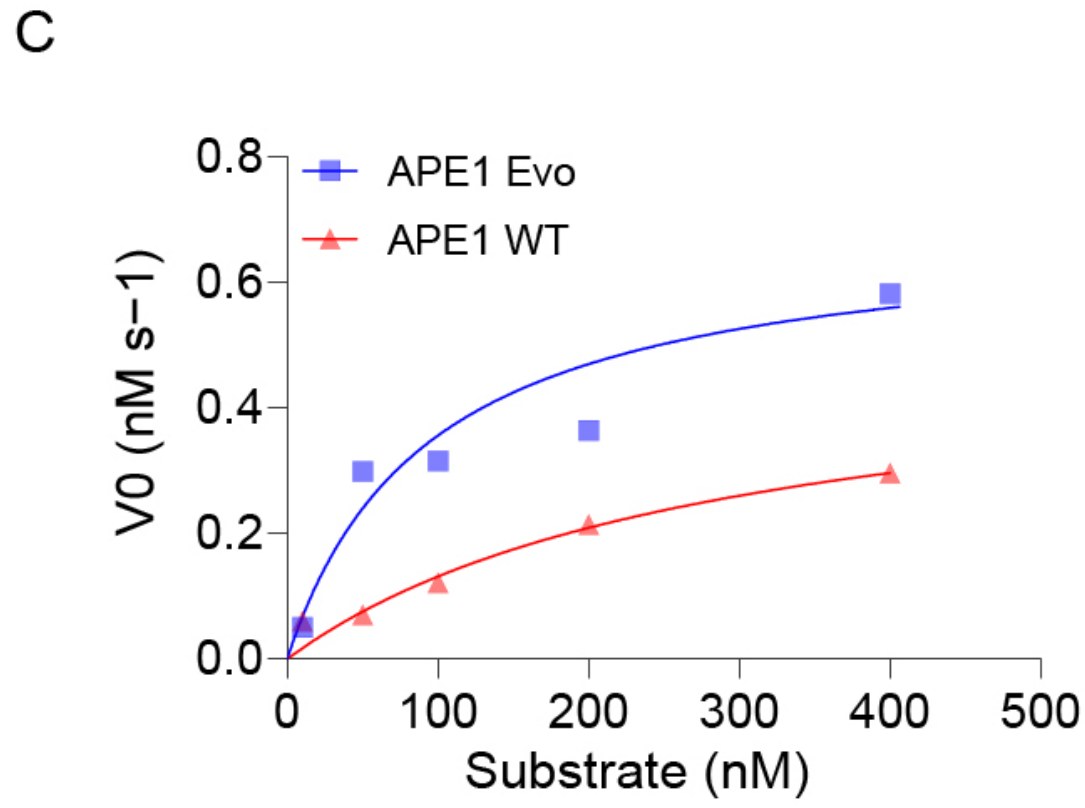

D

APE1	Km	Vmax	Vmax/Km (s−1)	kcat
WT	287.5 nM	0.5100 nM/s	0.00177	0.05100/s
Evo	92.69 nM	0.6515 nM/s	0.00703	0.0652/s

Apparent catalytic efficiency:

Evo/WT=0.00703/0.00177=3.97 folds

Supplementary Figure S5. Evo APE1 exhibits enhanced catalytic activity compared with WT APE1. (**A, B**) Time-dependent fluorescence signals of Evo APE1 (**A**) and WT APE1 (B) measured with increasing substrate concentrations from 10 to 400 nM. Initial reaction rates were obtained from the linear phase of each reaction. (**C**) Michaelis–Menten fitting of the initial velocities for Evo APE1 and WT APE1. (**D**) Summary of kinetic parameters. Evo APE1 showed a lower K_m and higher V_{max}/K_m than WT APE1, corresponding to an approximately 4-fold increase in apparent catalytic efficiency.

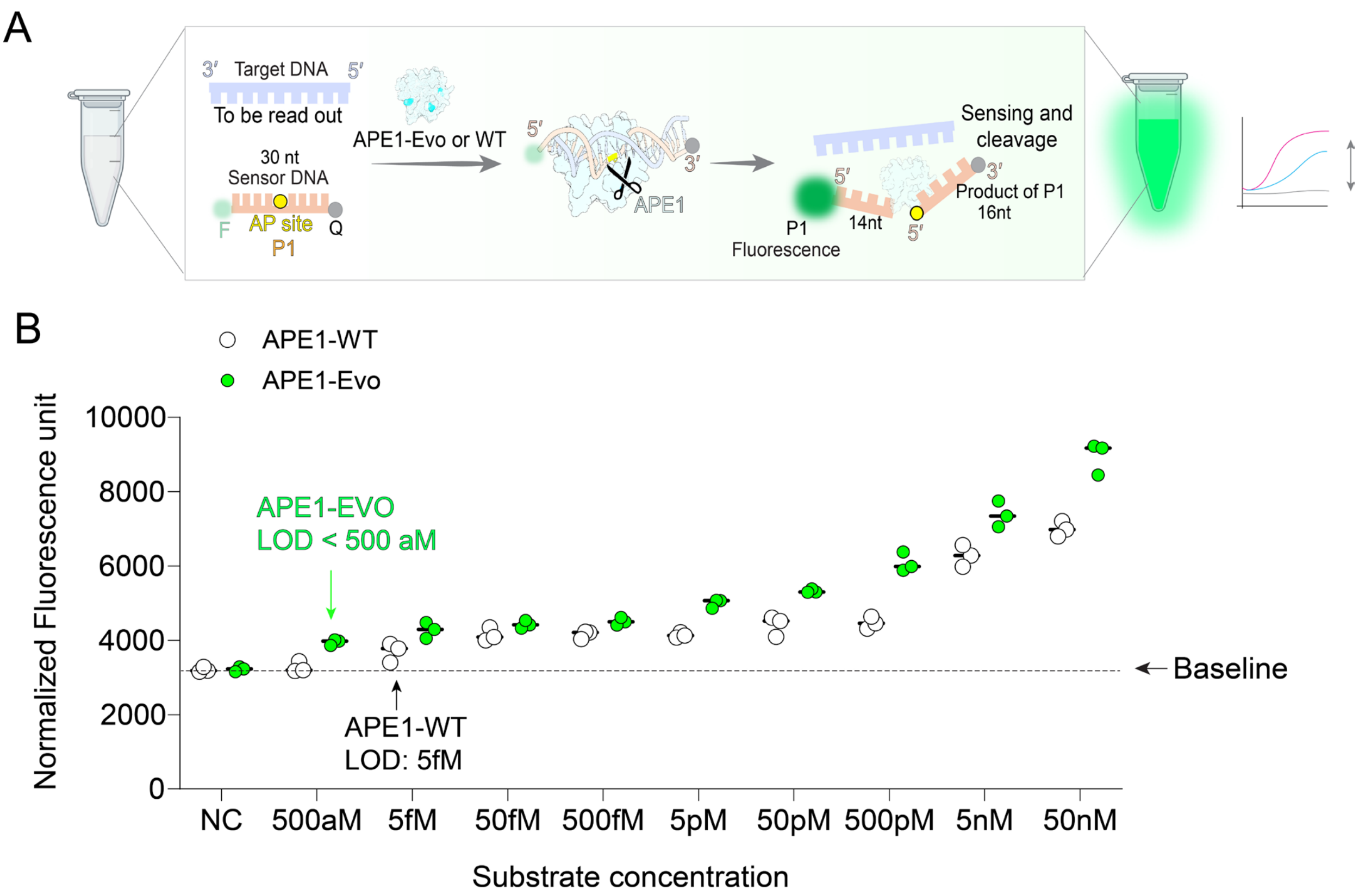

Supplementary Figure S6. APE1-Evo improves the sensing limit of AP-site cleavage. (A) Schematic of the direct APE1-based sensing assay. A 30-nt FAM/quencher-labelled AP-site sensor (P1) hybridizes with the target to form a cleavage-competent duplex. APE1 WT or APE1-Evo cleaves at the AP site to separate fluorophore and quencher, generating a fluorescence increase. This assay reports the intrinsic catalytic/engagement improvement of APE1-Evo without downstream amplification. **(B)** Sensitivity comparison of APE1 WT and APE1-Evo across a titration series of target/substrate concentrations. Fluorescence signals were normalized to the no-target control (NC, dashed baseline). APE1-Evo enables a markedly lower limit of detection than WT, improving the detectable concentration range by approximately one order of magnitude (APE1-Evo LOD < 500 aM versus APE1 WT LOD ~ 5 fM), consistent with enhanced AP-site cleavage efficiency.

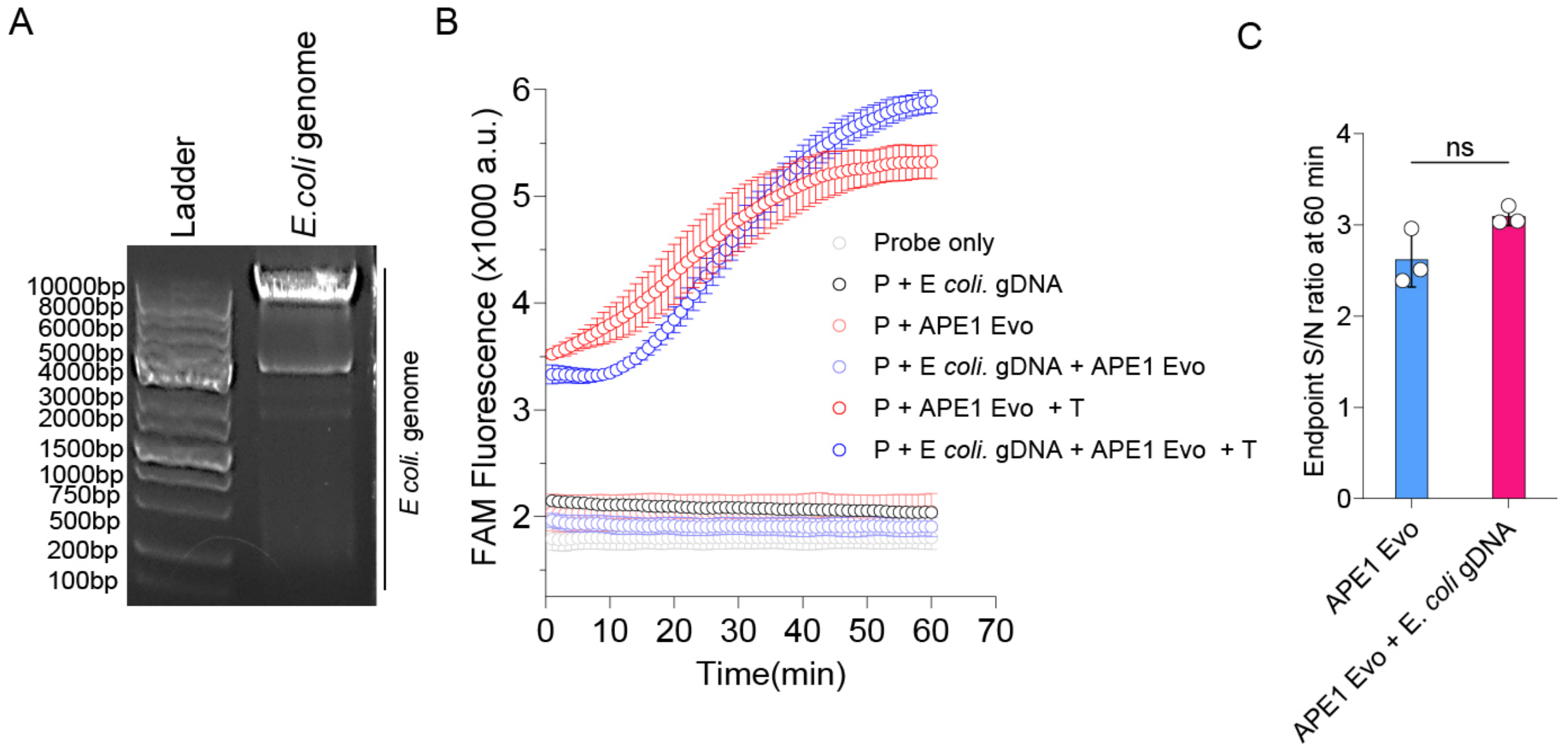

Supplementary Figure S7. Evo APE1 maintains specific and efficient probe cleavage in the presence of bacterial genomic DNA. (A) Agarose gel analysis of purified E. coli genomic DNA used as a complex nucleic-acid background in the assay. (**B**) Real-time fluorescence monitoring of probe cleavage under different reaction conditions. Reactions containing Evo APE1 and target showed robust fluorescence activation, whereas control reactions lacking Evo APE1 or target produced minimal signal. The presence of E. coli genomic DNA did not alter the target-dependent activation pattern. (**C**) Quantification of endpoint signal-to-noise ratios at 60 min. No significant difference was observed between reactions with Evo APE1 alone and those containing both Evo APE1 and E. coli genomic DNA, indicating that bacterial genomic DNA does not compromise the detection performance. Data are shown as mean ± SD; ns, not significant.

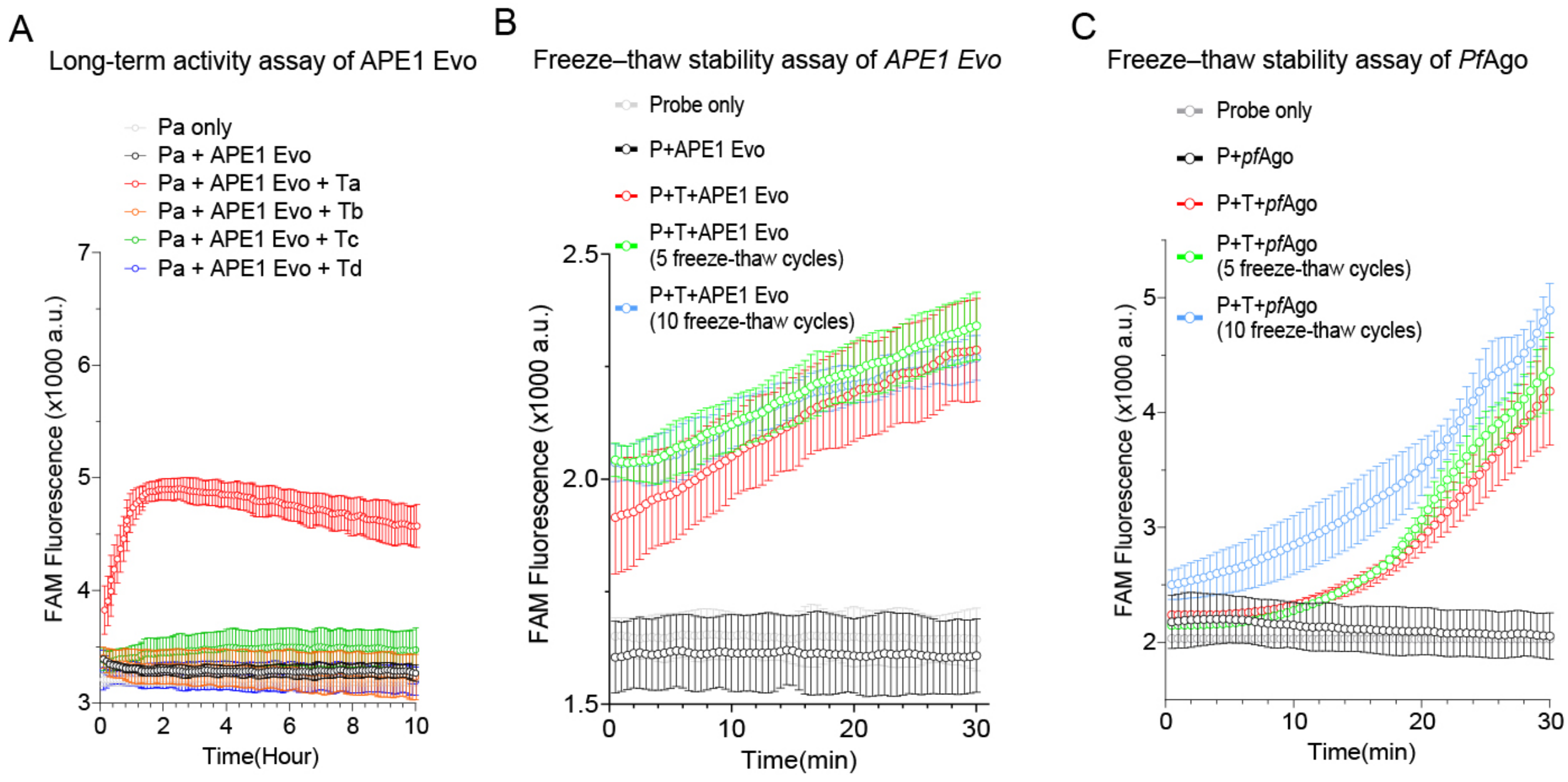

Supplementary Figure S8. Evo APE1 retains activity and specificity during prolonged reactions and after repeated freeze–thaw cycles. (**A**) Long-term activity assay of Evo APE1. Real-time fluorescence signals were monitored for up to 10 h using different target conditions. Evo APE1 maintained target-dependent probe cleavage over the prolonged reaction period, while non-target controls showed minimal fluorescence activation. (**B)** Freeze–thaw stability assay of Evo APE1. Evo APE1 retained probe-cleavage activity after 5 or 10 freeze–thaw cycles, with target-dependent fluorescence signals comparable to untreated protein. (**C**) Freeze–thaw stability assay of PfAgo. PfAgo retained target-dependent probe-cleavage activity after repeated freeze–thaw cycles, indicating that the assay components preserve detection efficiency and specificity after freeze–thaw treatment. Data are shown as mean ± SD.

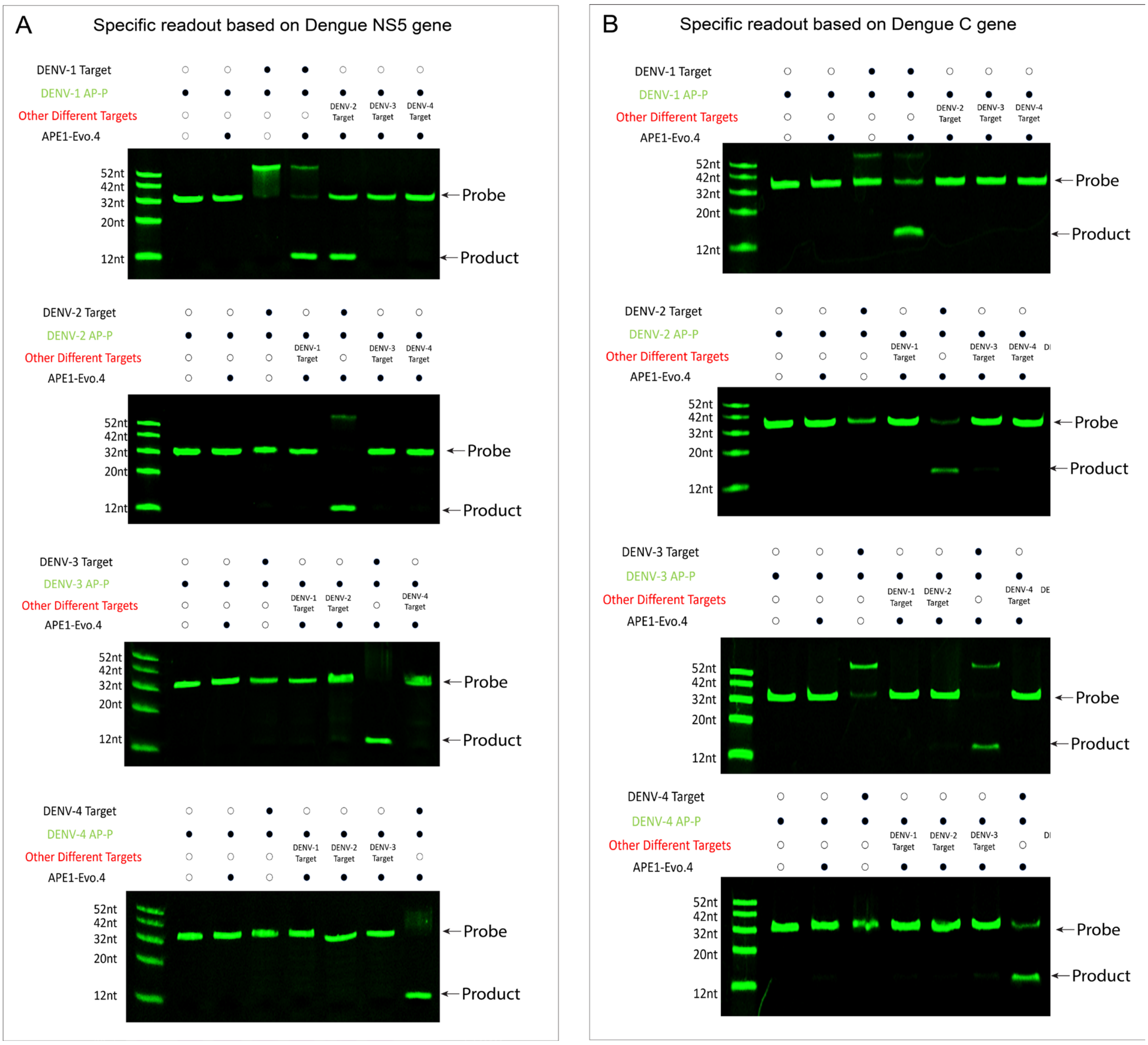

Supplementary Figure S9. Serotype-specific dengue readout using synthetic RNA mimics. (A) Gel-based specificity testing of NS5 gene-derived serotype-specific sensor probes. For each DENV serotype (DENV-1 to DENV-4), the corresponding AP-containing probe was challenged with its cognate synthetic RNA mimic as well as non-cognate RNA mimics representing the other serotypes. Robust product formation is observed only in the matched probe–target pair, whereas mismatched combinations yield minimal or no cleavage, indicating stringent serotype discrimination at the probe activation step. **(B)** Parallel specificity testing using C gene-derived serotype-specific probes. As in (A), each C-based sensor probe is selectively activated by its corresponding synthetic RNA mimic, with negligible cross-reactivity across other serotypes. Together, these results demonstrate that APE1-Evo.4-driven sensing preserves high serotype specificity at the level of rationally designed AP probes. Notably, the assays in this figure use **synthetic** RNA fragment mimics to validate probe logic and orthogonality at an early stage; pseudovirus-based validation of serotype-specific and pan-DENV detection is presented in the main text.

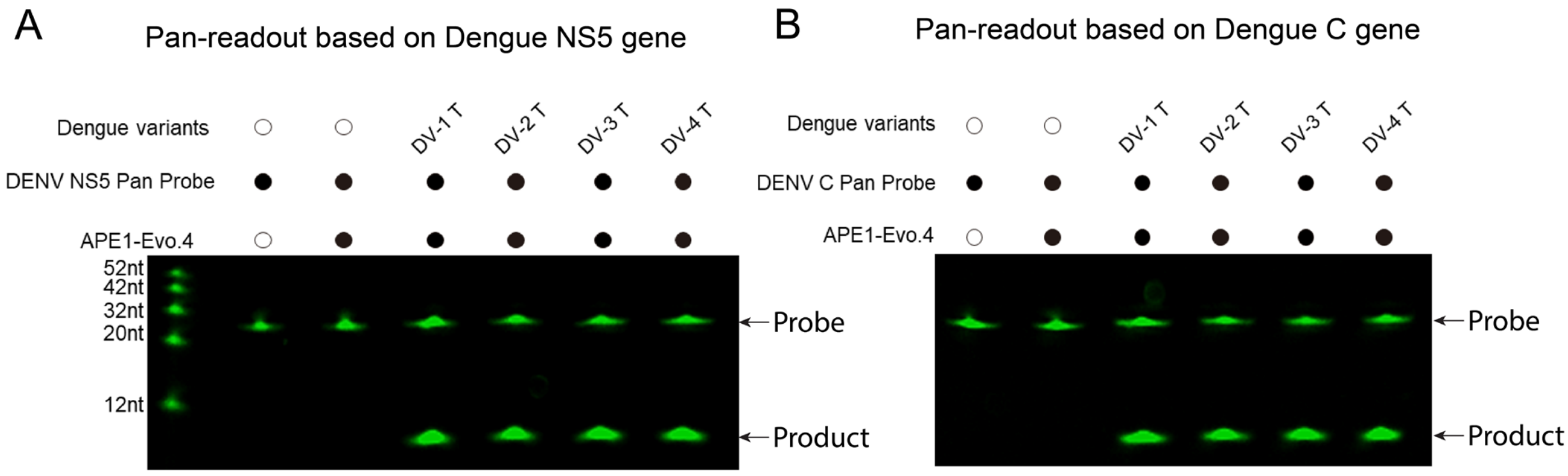

Supplementary Figure S10. Pan-DENV readout using NS5- and C-gene pan probes. **(A)** Pan-readout design and validation based on the dengue NS5 gene. A single NS5 pan probe containing an AP site was tested against RNA mimics corresponding to DENV-1 to DENV-4. In the presence of APE1-Evo.4, robust cleavage products were observed for all four serotypes, whereas no cleavage was detected in control conditions lacking target or enzyme, supporting broad pan-DENV compatibility of the NS5 pan probe. **(B)** Pan-readout design and validation based on the dengue C gene. Analogous assays using a C-gene pan probe showed efficient cleavage across all four DENV serotype RNA mimics with APE1-Evo.4. Together with the serotype-specific readout in Supplementary Figure S6, these results demonstrate that the APE1-Evo-enabled sensing scheme can be configured either for strict serotype discrimination or for unified pan-DENV detection at the probe-design level.

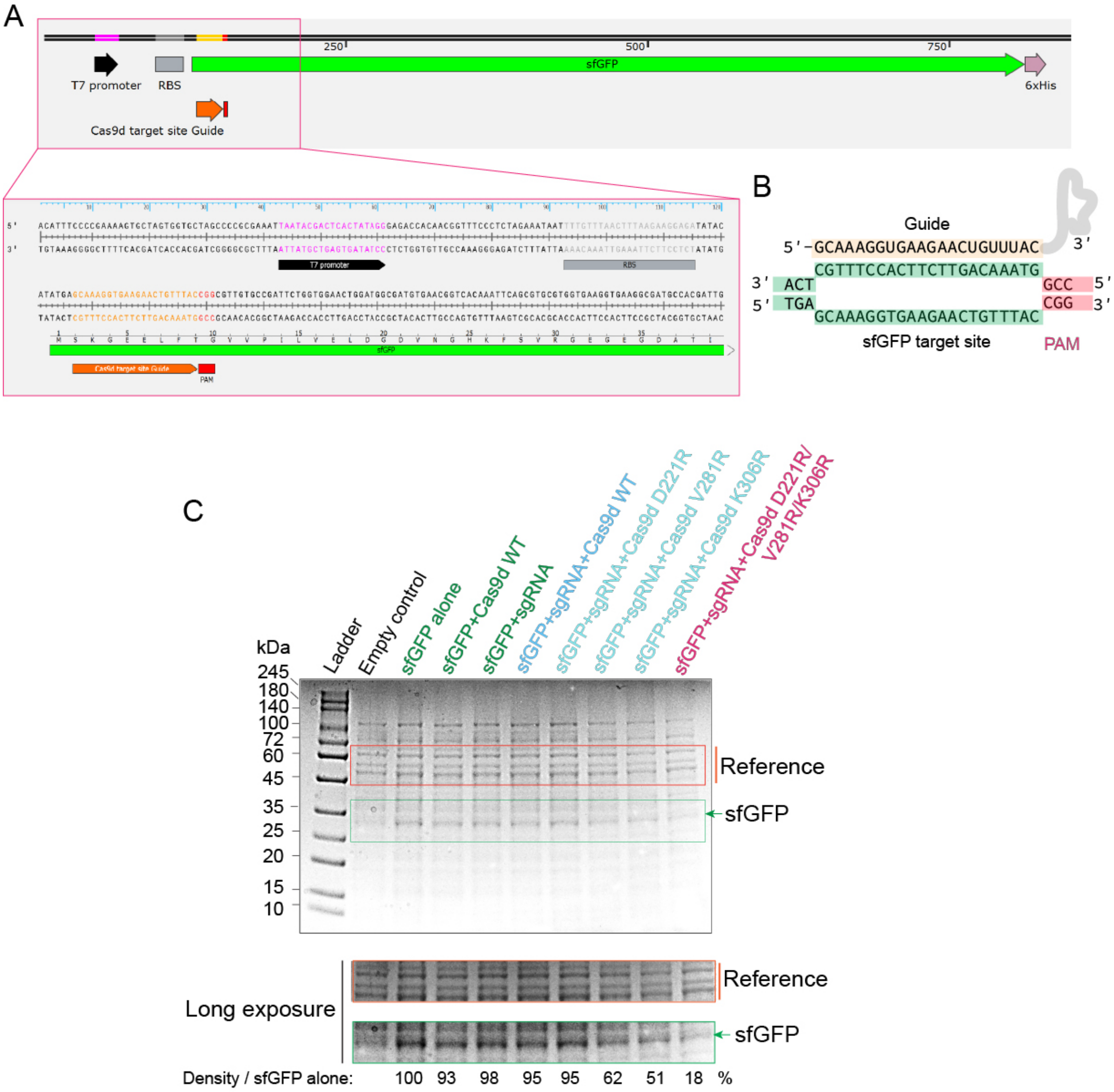

Supplementary Figure S11. Design and validation of the sfGFP reporter assay for Cas9d activity. (A) Schematic of the sfGFP reporter plasmid containing the T7 promoter, RBS, sfGFP coding sequence, and the Cas9d target site. The enlarged region shows the target sequence and guide-binding site. (B) Design of the Cas9d guide RNA targeting the sfGFP reporter, with the target strand, non-target strand, and PAM indicated. (C) SDS–PAGE analysis of sfGFP expression after Cas9d targeting. Reduced sfGFP band intensity indicates stronger Cas9d-mediated disruption of sfGFP expression. The engineered Cas9d variants, especially the D221R/V281R/K306R combination, showed stronger reduction of sfGFP expression than WT Cas9d. A reference protein band was used for normalization.

Supplementary Table S1. Samples information

No.	Age	Types	Main Symptoms
1	3 years	FA	Fever, cough, sore throat
2	< 1 years	FA	Fever, cough, sore throat
3	5 years	FA	Fever, cough, sore throat
4	4 years	FA	Fever, cough, sore throat
5	5 years	FA	Fever, cough, sore throat
6	< 1 years	FA	Fever, cough, sore throat
7	6 years	FA	Fever, cough, sore throat
8	4 years	FA	Fever, cough, sore throat
9	12 years	FA	Fever, cough, sore throat
10	11 years	FA	Fever, cough, sore throat
11	10 years	FA	Fever, cough, sore throat
12	13 years	FA	Fever, cough, sore throat
13	9 years	FA	Fever, cough, sore throat
14	4 years	FA	Fever, cough, sore throat
15	2 years	FA	Fever, cough, sore throat
16	8 years	FA	Fever, cough, sore throat
17	2 years	FA	Fever, cough, sore throat
18	7 years	FB	Fever, cough, sore throat
19	4 years	FB	Fever, cough, sore throat
20	5 years	FB	Fever, cough, sore throat
21	10 years	FB	Fever, cough, sore throat
22	11 years	FB	Fever, cough, sore throat
23	13 years	Normal	/
24	2 years	Normal	/
25	16 years	Normal	/
26	3 years	Normal	/
27	< 1 years	Normal	/
28	13 years	Normal	/
29	4 years	Normal	/
30	6 years	Normal	/
31	3 years	Normal	/
32	8 years	Normal	/
33	9 years	Normal	/
34	2 years	Normal	/
35	5 years	Normal	/
36	4 years	Normal	/
37	2 years	Normal	/
38	3 years	Normal	/
39	10 years	Normal	/
40	15 years	Normal	/

Supplementary Table S2. Comparison of NAPTUNE-V2.0 with representative CRISPR-based diagnostic platforms

Platform	Representative LOD	Approx. copies/uL	Target amplification
SHERLOCK	2.1 aM	~1.25 copies/uL	Yes
DETECTR	~1 aM	~0.6 copies/uL	Yes
NAPTUNE-V2.0	<500 aM	<300 copies/uL	No

Legends for Datasets S1 to S2 (Other supporting materials)

Supplementary Dataset S1. ARGENT multi-scale local hotspot scoring and ConSurf-adjusted ranking for APE1.

This Excel file provides the residue-level outputs of the ARGENT Arg-scanning pipeline for APE1. For each position within residues 43–318, we report structural descriptors, multi-scale local-hotspot scores computed at two neighborhood windows (w8 and w18), and the integrated and ConSurf-adjusted rankings used to prioritize experimental candidates. Columns include residue index and identity (**resnum, resname**), minimum distance to DNA phosphate (**dmin**), solvent exposure (**exposure**), raw Arg-substitution affinity proxy (**affinity_raw**), and the normalized local hotspot scores derived from the two window sizes (**local_norm_w8, local_norm_w18**). The multi-scale mean hotspot score (**hotspot_mean**) summarizes the local context across both windows. Evolutionary constraint is captured by residue-wise ConSurf identity/grade (**resname_consrf, consurf_grade**) and a normalized conservation factor (**consurf_norm**). The combined hotspot score after applying conservation-based weighting or filtering is reported as **hotspot_adjusted**, followed by downstream rule-based adjustment (**hotspot_adjusted_c7+0.01**) and final prioritization (**rank_c7**). This dataset underpins the selection of APE1 Arg-scan candidates by explicitly illustrating how multi-scale local context and evolutionary permissiveness jointly shape the final shortlist.

Supplementary Dataset S2. Distance–exposure and two-window local hotspot scores for APE1 Arg-scanning.

This dataset summarizes the distance-to-phosphate and surface-exposure features used in the early stage of the ARGENT pipeline, together with the two-scale local environment scores derived from window-based neighborhood analysis. For each residue in APE1, we report residue index and identity (**Residue_number, Residue_name**), the minimum distance to the nearest DNA phosphate (**X_dminP_A**), and the residue surface exposure (**Y_exposure**). Based on these geometric descriptors, we calculate a local hotspot score within two neighborhood windows (**window 8** and **window 18**), recorded as **Size_localHotspotScore**. A lightly shifted version (**Size_localHotspotScore + 0.01**) is included to facilitate downstream ranking and visualization consistency across datasets. The column **Average_Size_localHotspotScore + 0.01** aggregates the two window scores, providing a compact, multi-scale estimate of local Arg-scan favorability. This table serves as the primary quantitative basis for the distance–exposure maps and for identifying residues embedded in geometry-permissive DNA-contact microenvironments prior to applying evolutionary (ConSurf) constraints.

Supplementary Dataset S3. Uncropped gel images.
Uncropped fluorescence gel scans corresponding to the cropped images shown in the indicated main and supplementary figures. Lanes are presented in the same order as in the assembled figures. Molecular size markers are shown on the left. Exposure and contrast were adjusted uniformly across each gel.

Supplementary Data S4. Preliminary data.
This dataset compiles preliminary AI-computed outputs and experimentally acquired raw measurements supporting this study. It includes all primary values underlying the quantitative analyses reported in the main text and supplementary figures, including ARGENT residue-level scoring outputs, window-based local hotspot metrics (w8 and w18), ConSurf-related annotations, distance–exposure parameters, and the corresponding biochemical and assay-derived raw readouts used for statistical summaries. These files provide the source data for plotting, ranking, and comparative analyses across APE1 variants, combinatorial designs, and downstream detection modules.

Reference

1. Emamjomeh A, Choobineh D, Hajieghrari B, MahdiNezhad N, Khodavirdipour A. DNA-protein interaction: identification, prediction and data analysis. Mol Biol Rep. 2019;46(3):3571-96. Epub 2019/03/28. doi: 10.1007/s11033-019-04763-1. PubMed PMID: 30915687.
2. Babaei S, Hulsman M, Reinders M, de Ridder J. Detecting recurrent gene mutation in interaction network context using multi-scale graph diffusion. BMC Bioinformatics. 2013;14:29. Epub 2013/01/25. doi: 10.1186/1471-2105-14-29. PubMed PMID: 23343428; PMCID: PMC3626877.
3. Ashkenazy H, Erez E, Martz E, Pupko T, Ben-Tal N. ConSurf 2010: calculating evolutionary conservation in sequence and structure of proteins and nucleic acids. Nucleic Acids Res. 2010;38(Web Server issue):W529-33. Epub 2010/05/19. doi: 10.1093/nar/gkq399. PubMed PMID: 20478830; PMCID: PMC2896094.
