# Supplementary figures and images for: Computation and Structure‐Guided Arginine Scanning Engineers a Hyperactive AP Endonuclease for Multiplex Viral RNA Sensing
Source: Adv Sci (Weinh). 2026 Jul 24:e76769. Online ahead of print. doi: 10.1002/advs.76769 (PMC13398136; doi:10.1002/advs.76769)

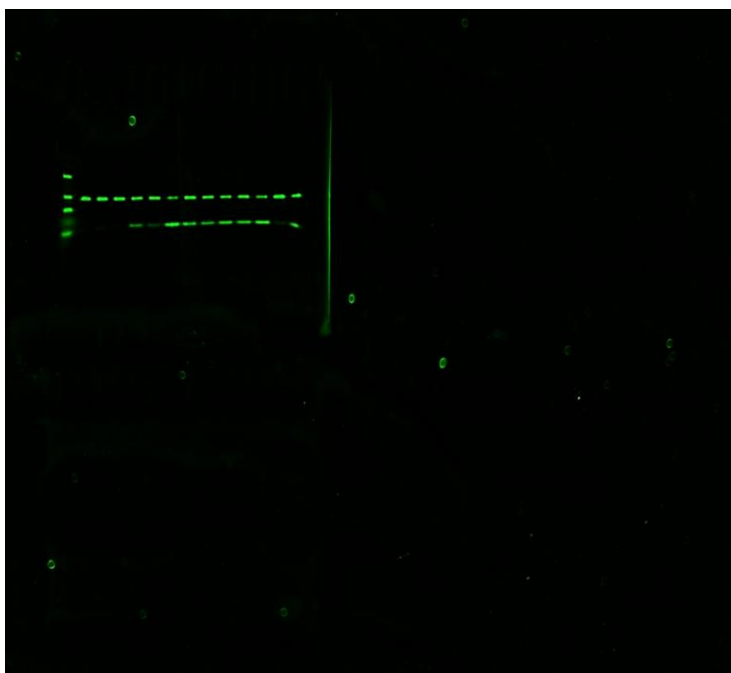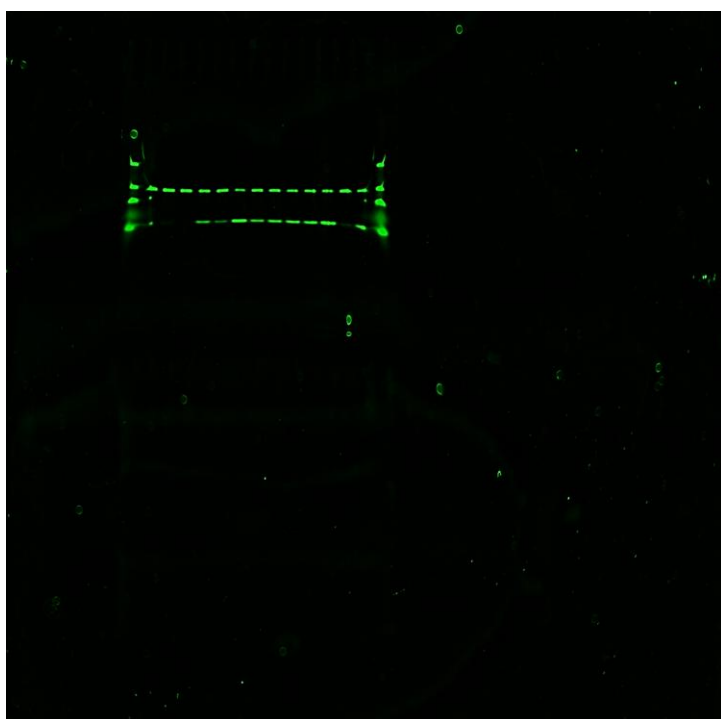

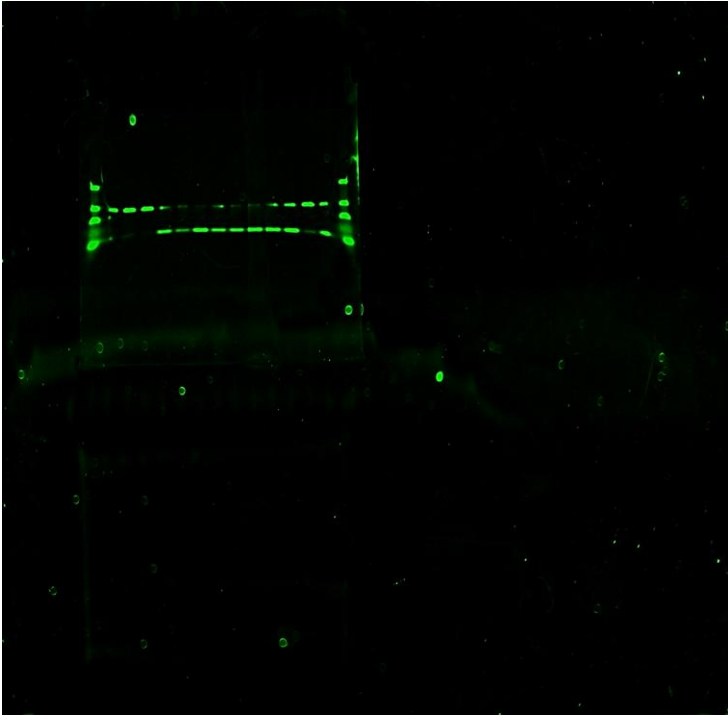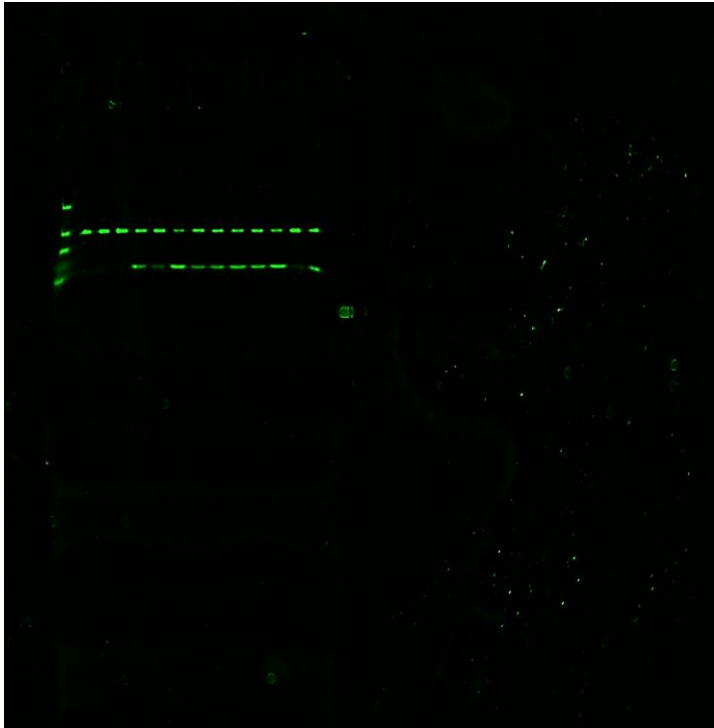

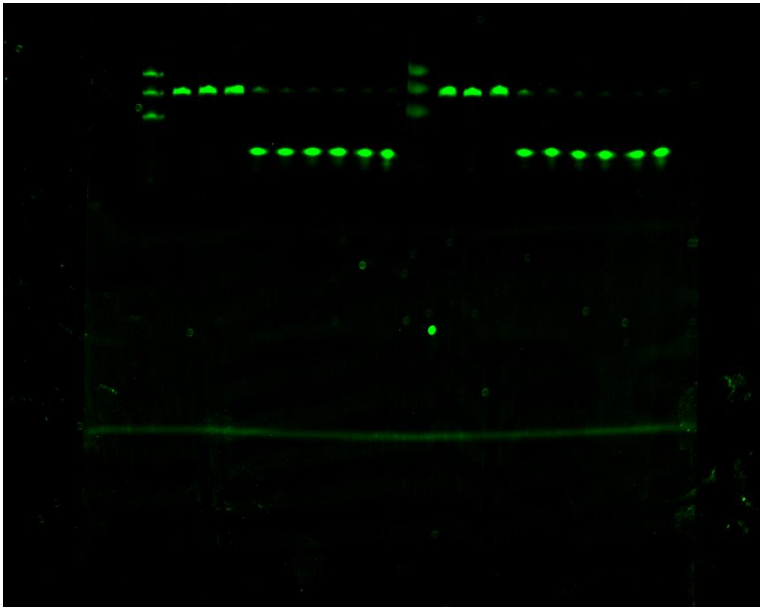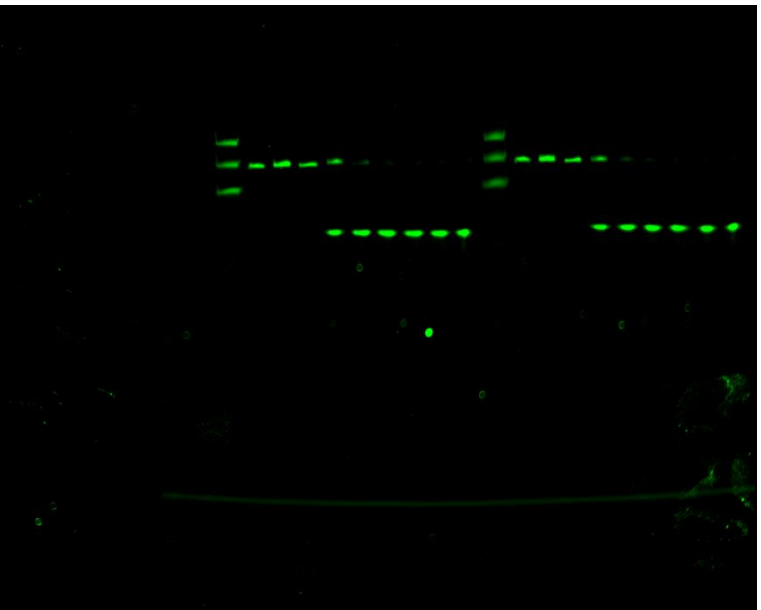

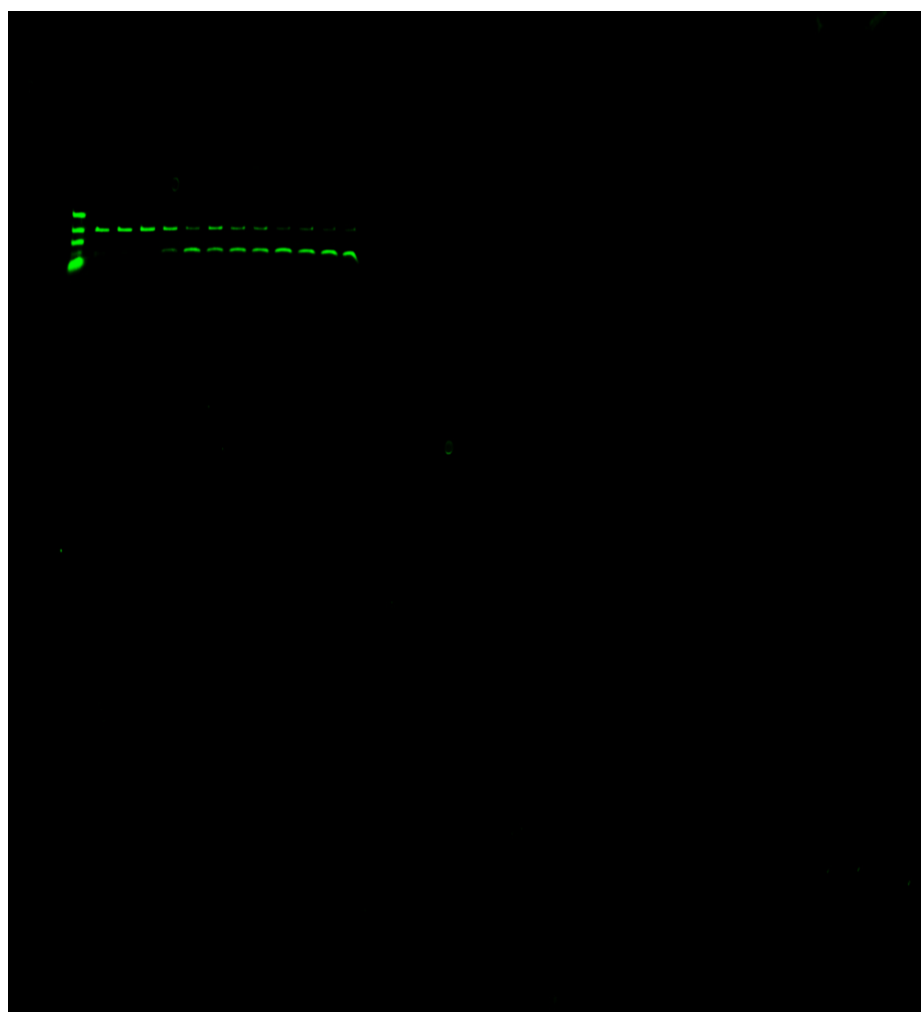

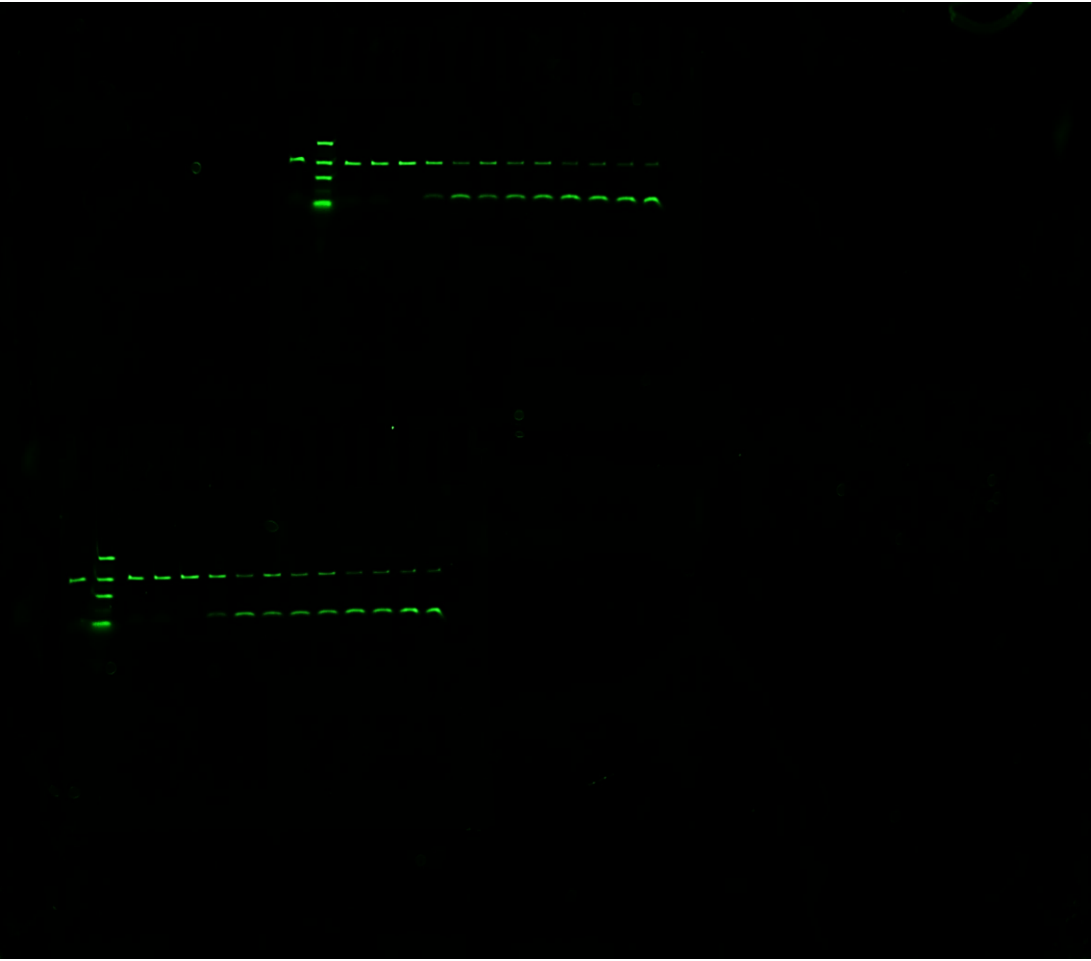

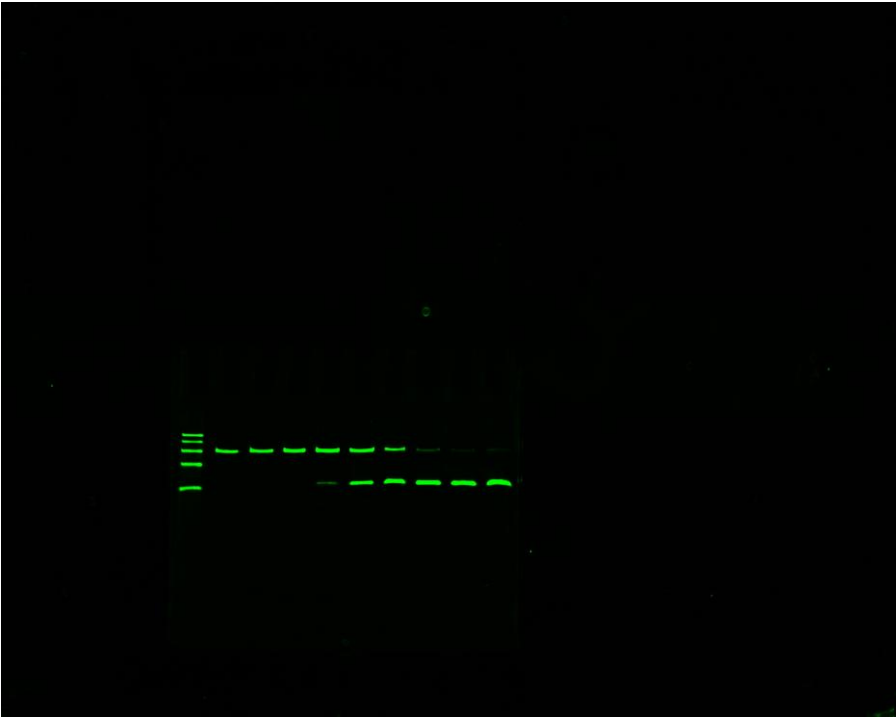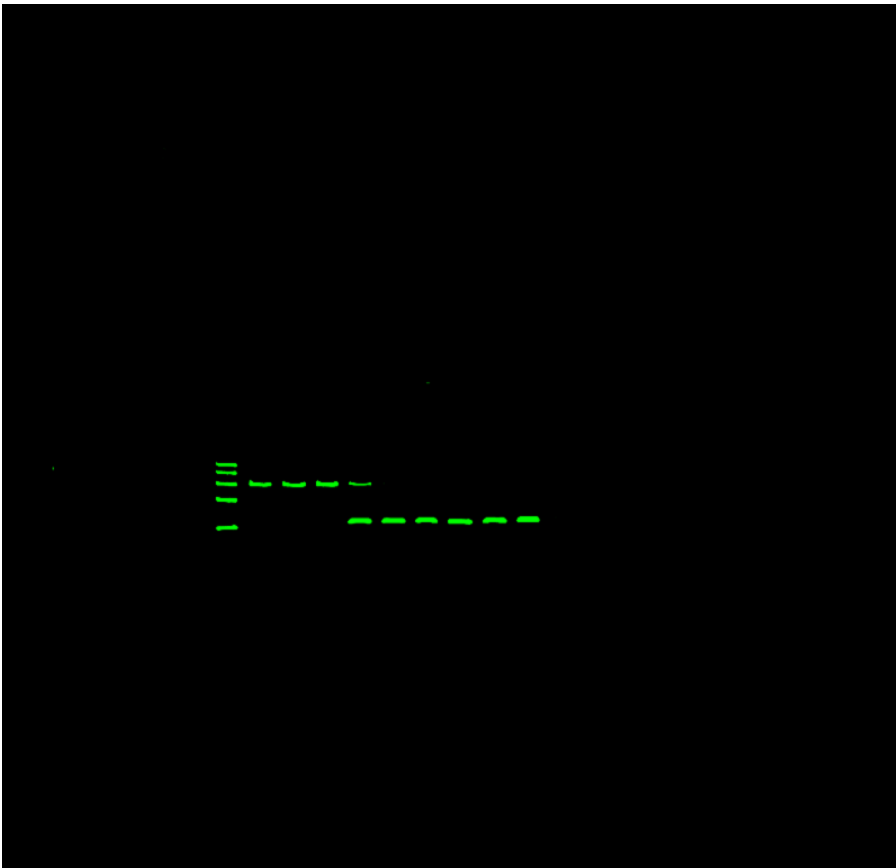

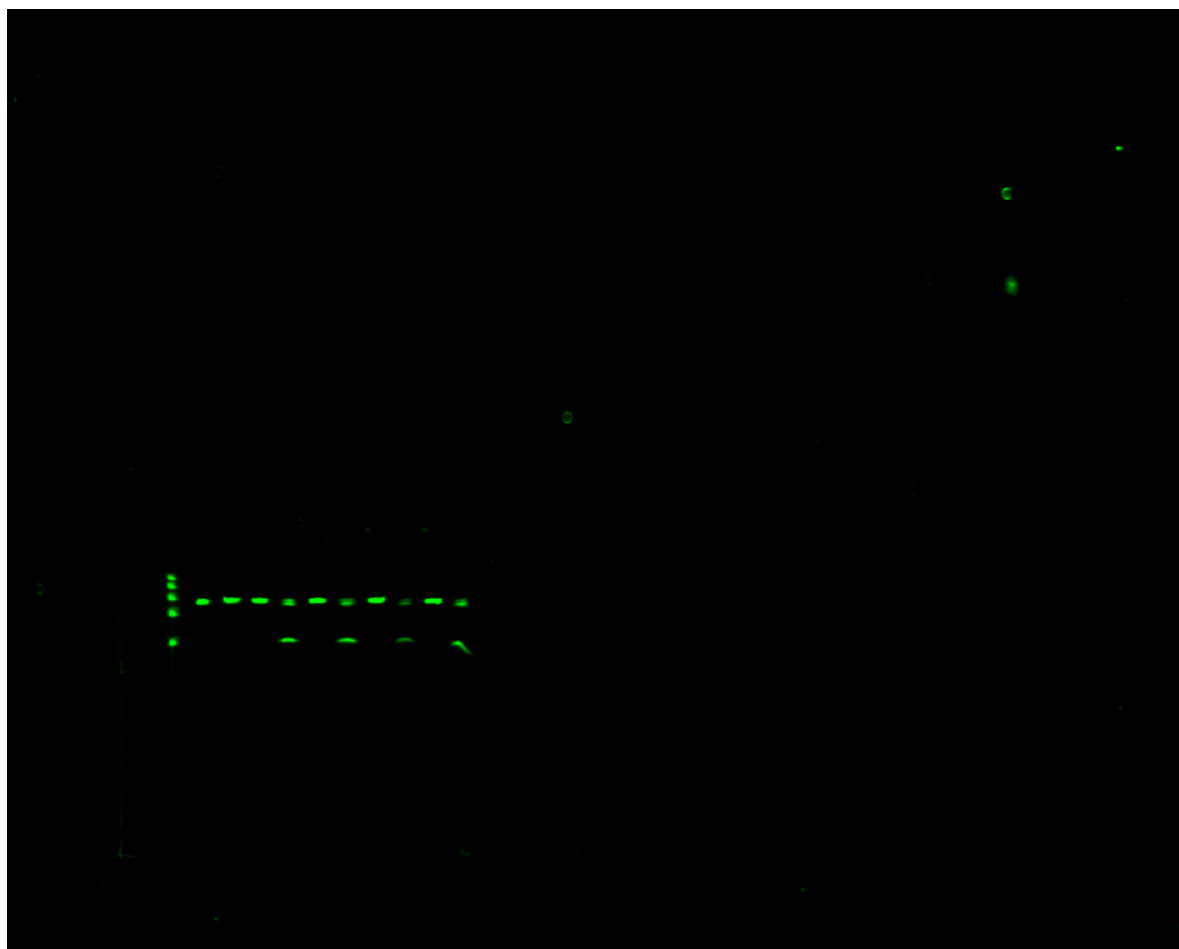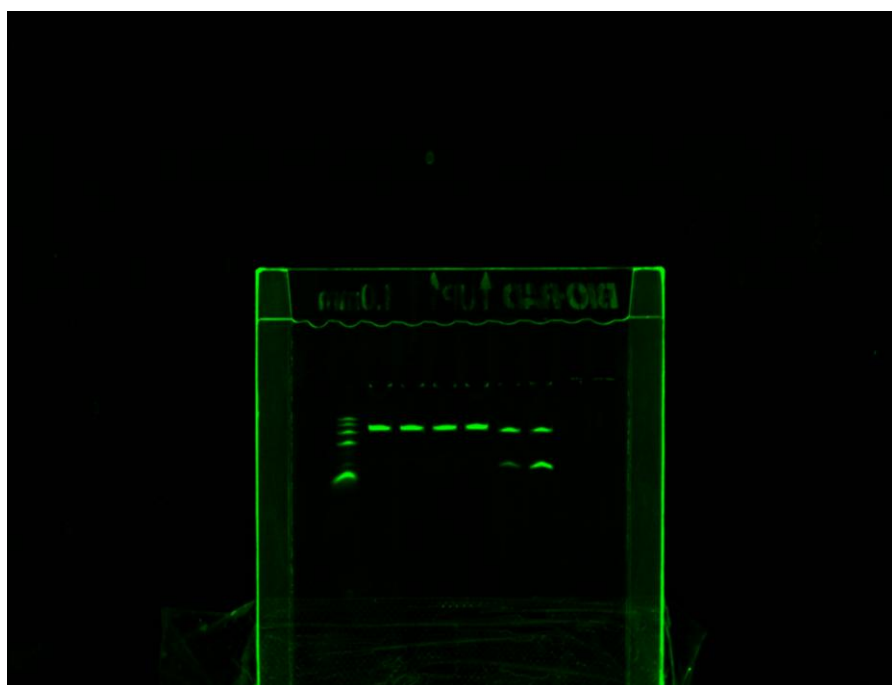

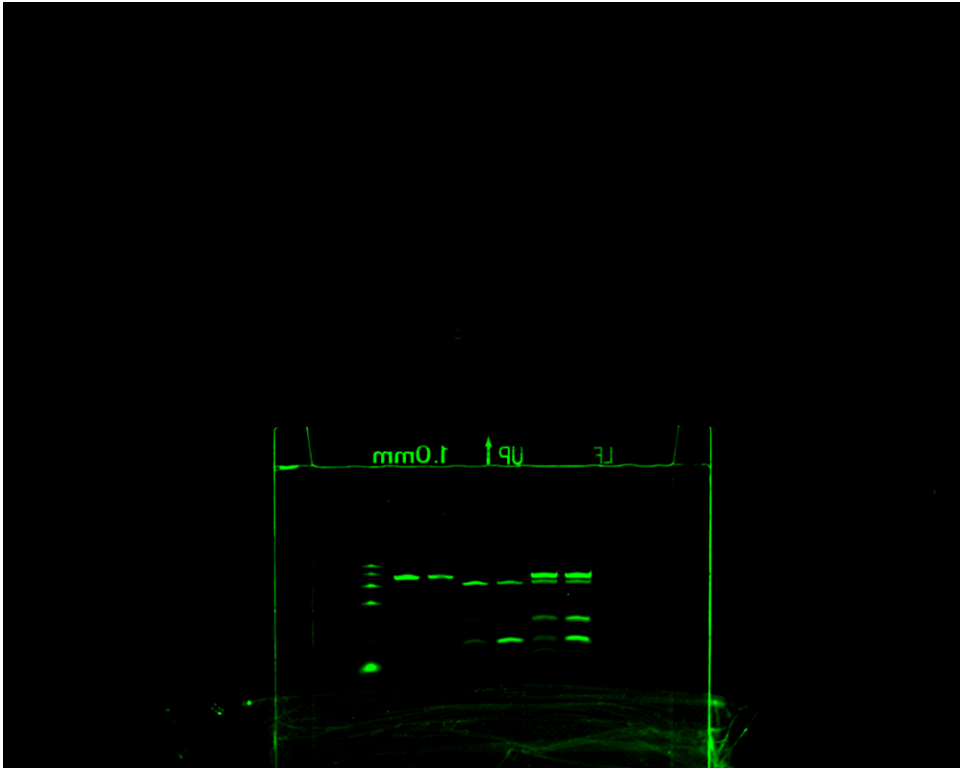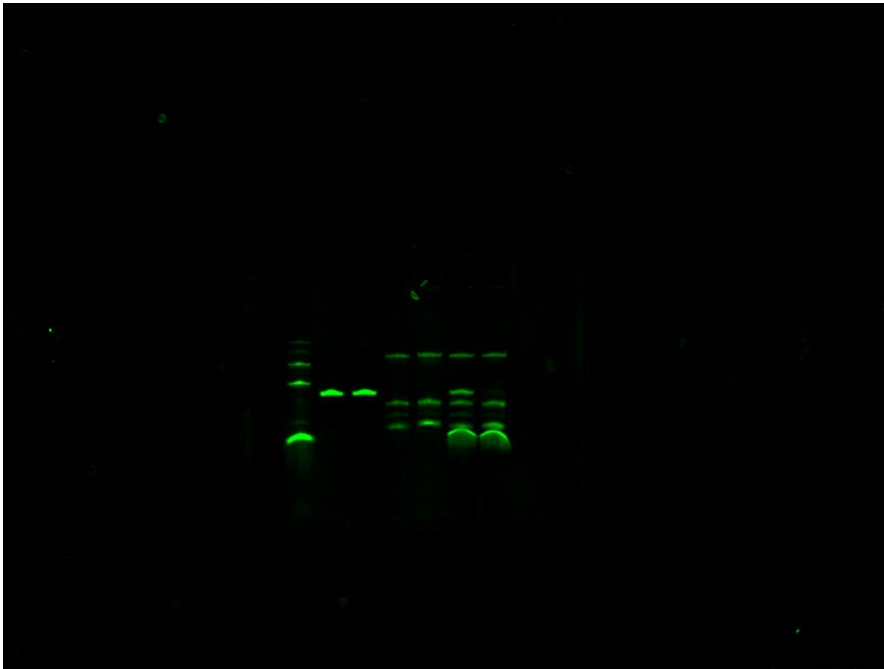

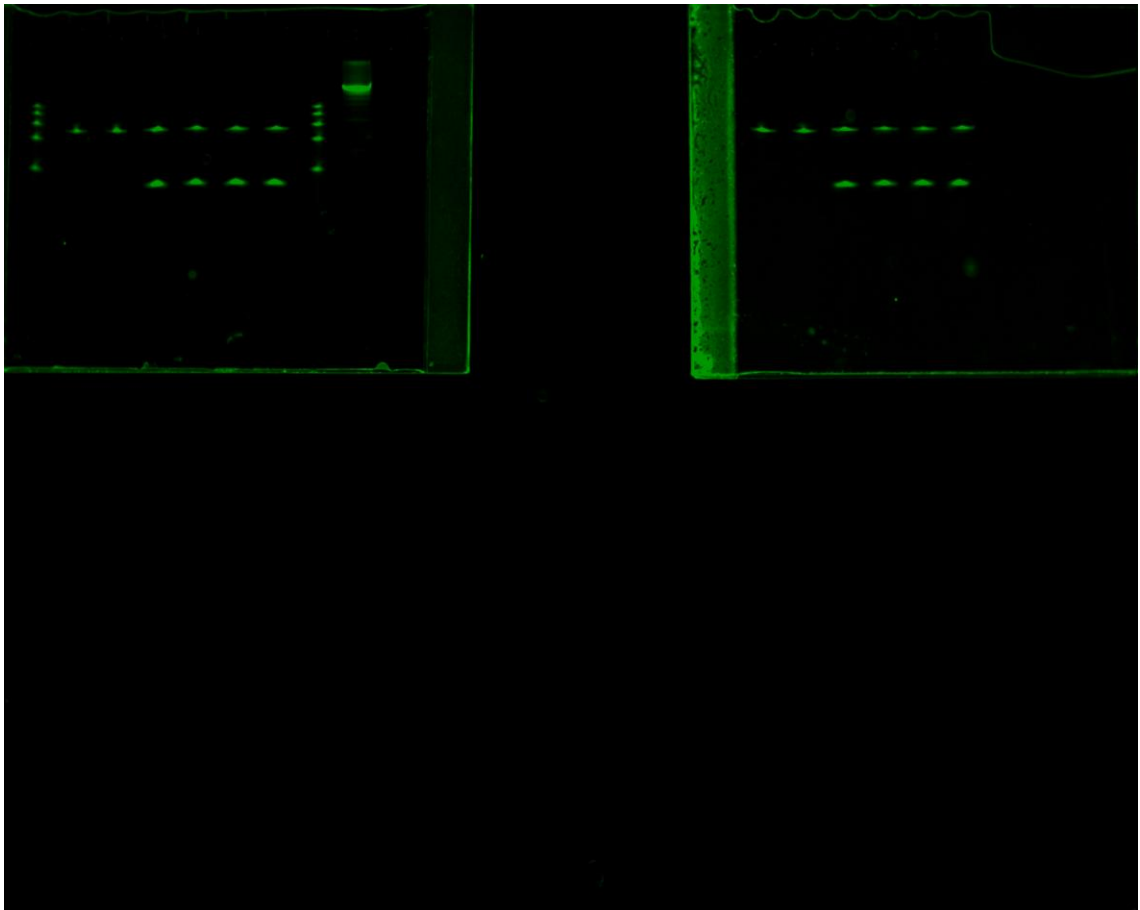

Supplement: Supplementary file 4 — Supporting File 4: advs76769‐sup‐0004‐Data.zip. [file ADVS-9999-e76769-s002.zip › advs76769-sup-0005-uncropped gels.pdf]
